# Ultrasound activated herbal bio-heterojunctions for self-catalytic regulation and bacterial cuproptosis-like death in the treatment of implant infection

**DOI:** 10.1038/s41392-025-02388-4

**Published:** 2025-09-19

**Authors:** Yan Yue, Shuoyuan Li, Qiang Su, Xufeng Wan, Qiaochu Li, Zhuang Zhang, Hong Xu, Fuyuan Zheng, Yangming Zhang, Le Tong, Jian Cao, Long Zhao, Xiaoting Chen, Qi Li, Yi Zeng, Haoyang Wang, Yi Deng, Zongke Zhou, Duan Wang

**Affiliations:** 1 Orthopedic Research Institute and Department of Orthopedics, West China Hospital, Sichuan University, Chengdu, 610041 China; 2 Department of Orthopedics, the First Affiliated Hospital, Chongqing Medical University, Chongqing, 400016 China; 3 Department of Emergency Medicine, West China Hospital, Sichuan University, Chengdu, 610041 China; 4 Animal Experimental Center, West China Hospital, Sichuan University, Chengdu, 610041 China; 5 School of Chemical Engineering, Sichuan University, Chengdu, 610065 China

**Keywords:** Biomaterials, Nanobiotechnology

## Abstract

Conventional antibiotic strategies often fail to consistently suppress escaping planktonic bacteria and even induce antibiotic resistance, allowing implant-associated infections (IAIs) to persist. In this study, we demonstrated an antibiotic-free coating engineered with an herbal bioheterojunction featuring a shell-in-shell structure where Cu_2_O forms the core, strontium (Sr) is loaded in the inner shell, and curcumin (Cur) is nucleated in situ at the outer heterointerface (Cu_2_O-Sr/Cur). Ultrasound-triggered reactive oxygen species (ROS) generation by the outer heterostructure (Cu_2_O/Cur), coupled with Cu(I)-induced cuproptosis-like bacterial death, achieved antibacterial rates of 99.56% against *S. aureus* and 99.43% against *E. coli*. When ultrasonication ceases, the released Cu(I) undergoes disproportionation reactions to form Cu(II), which can chelate with Cur to form Cu-Cur metal complexes. These complexes exhibit enhanced antioxidative properties through self-catalytic regulation, the scavenging of ROS, and the activation of anti-inflammatory M2 macrophage phenotype. Moreover, strontium release from the inner shell simultaneously suppressed osteoclast activity and promoted osteogenesis, resulting in trabecular number and thickness increases of 129.03% and 56.71%, respectively, compared with those in control group. Therefore, our work establishes a sequential treatment strategy for the antibacterial properties and osteointegration ability of IAIs.

## Introduction

Despite advances in biomaterials and surgical techniques, implant-associated infections (IAIs) continue to jeopardize the success of bone defect repair, often culminating in implant failure and adverse patient outcomes.^1,2^ The systematic use of antibiotics or implant replacement surgery may be an effective clinical option to address this issue.^3^ However, limited drug targeting and bacterial drug resistance may compromise antibacterial efficacy. Additionally, both the abundant release of endotoxins following bacterial death triggers excessive inflammation,^4^ and the induction of osteoclasts by the bacterial microenvironment restricts the repair of bones.^5^ Consequently, the development of nonantibiotic coatings that can provide effective antimicrobial, anti-inflammatory, and osseointegration therapies is highly important for combating IAIs.

Sonodynamic therapy (SDT) represents an emerging antibacterial strategy leverages ultrasound to induce sonosensitizer activation, which enables localized generation of reactive oxygen species (ROS).^6–8^ ROS exhibit broad-spectrum bactericidal effects by inflicting oxidative stress on vital biomolecules, including proteins, membrane lipids, and nucleic acids.^9–11^ Despite its therapeutic potential, SDT efficacy is fundamentally limited by the transient nature and poor diffusivity of ROS, exacerbated by local hypoxia.^12–14^ Consequently, various approaches have been explored to optimize SDT efficacy, including enhancing the bacterial targeting of sonosensitizers,^15,16^ alleviating hypoxia,^17^ and integrating metabolic therapies.^18^ Meanwhile, excessive accumulation of ROS may exacerbate inflammation and induce cellular dysfunction, thereby impeding wound healing.^19^ Therefore, rational regulation of redox homeostasis is equally crucial. Elucidating the mechanisms underlying bacterial death is pivotal to the rational design of antimicrobial materials. Many studies have demonstrated that ROS kill bacteria through multiple pathways. Emerging evidence suggests that bacterial death often results from the synergy of several mechanisms, including metal ion-induced metabolic disruption.^20^ Therefore, systematically identifying and elucidating these diverse bacterial death pathways not only facilitates a comprehensive evaluation of the antibacterial performance of materials, but also reveals potential synergistic or compensatory mechanisms, thereby offering theoretical guidance for functional optimization.

Curcumin (Cur), a naturally derived polyphenolic sonosensitizer, has been widely utilized in SDT due to its broad-spectrum antibacterial and antitumor activities, as well as its excellent biosafety profile.^21^ However, free Cur faces significant clinical limitations due to its inadequate aqueous solubility, poor stability in physiological environments, and restricted bioavailability.^22,23^ Notably, the β-diketone group within the Cur molecule possesses a high degree of conjugation, enabling it to coordinate stably with various metal ions (e.g., Fe(II), Cu(II), Zn(II), Cu(I)), thus enhancing its bioavailability.^24,25^ In addition, Cur–CuS complexes have shown excellent sonosensitizing efficacy against *Staphylococcus aureus* infections.^26^ Despite the limitations of free Cur, its strong metal-coordination ability makes it highly promising for the development of composite systems with enhanced ultrasound responsiveness. To this end, bioheterojunctions (bio-HJs) which are constructed by combining two semiconductors with distinct bandgaps, have emerged as effective nanoreactors for enhancing SDT. Upon ultrasound irradiation, the interfacial electron transfer between components within the bio-HJs markedly promotes the generation of ROS.^9,27^ Given Cur inherent properties and its strong affinity for metal ions, it represents an ideal candidate for interface engineering in bio-HJ design, thereby further increasing ROS production and improving sonodynamic efficacy. In addition, Cur excellent antioxidant and anti-inflammatory properties have garnered increasing interest.^28,29^ It can exert antioxidant effects by eliminating ROS through electron transfer or hydrogen-donation mechanisms^30^ and modulate inflammation by targeting relevant signaling molecules, thereby downregulating pro-inflammatory cytokines such as TNF-α, IL-1, and IL-6.^31^ Recent studies have demonstrated that Cur-based functional materials can alleviate oxidative stress and inflammatory responses, facilitate granulation tissue formation and angiogenesis, and thereby accelerate wound healing.^32^

**Fig. 1:**
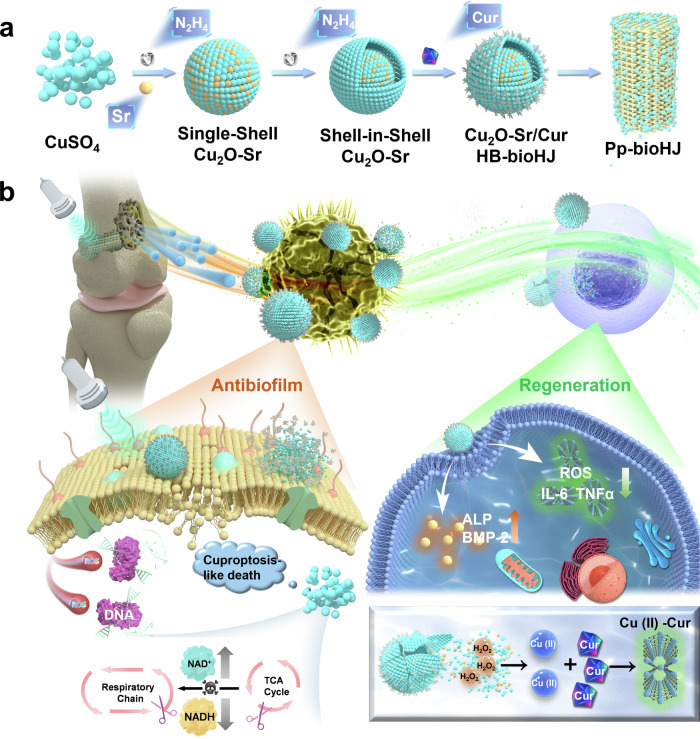
Schematic of the synthesis and IAI Treatment. **a** Schematic of the synthetic of Pp-bioHJs. **b** Schematic depicting the antibacterial strategy of Pp-bioHJs for IAI therapy. The treatment enhances bacterial killing by ultrasound-triggered ROS generation from HB-bioHJs and intracellular Cu(I) overload–induced cuproptosis-like death. The bone scaffold exhibited anti-inflammation, immunoregulation, and osteogenic via Cu(II)-Cur-activated self-catalysis regulation and Sr-mediated treatment in an infected bone implant model

Cuprous oxide (Cu_2_O) nanoparticles, as representative p-type semiconductors, exhibit distinctive optical, electronic, and catalytic properties and have been extensively explored for their antibacterial applications.^33,34^ Cu_2_O has diverse structural morphologies; notably, its shell-in-shell structure^35^ may enable sequential antibacterial action and tissue regeneration by leveraging distinct functionalities of each shell layer. Excess intracellular copper disturbs mitochondrial homeostasis by promoting the misfolding and accumulation of lipoylated enzymes, particularly dihydrolipoamide S-acetyltransferase (DLAT), and disrupting iron–sulfur cluster–dependent metabolic pathways in the tricarboxylic acid cycle.^36–38^ This process induces profound proteotoxic stress that results in tumor cell death, which offers new perspectives on copper-mediated bacterial death.^39^ Therefore, SDT in conjunction with metabolic disruption therapy for bacterial copper overload can achieve highly effective and sustained antibacterial action. In addition, the released Cu(I) can undergo disproportionation reactions with hydrogen peroxide in an acidic environment to produce Cu(II),^40^ which subsequently engages in a self-catalysis-induced chelation reaction to form Cu(II)-Cur. Cu(II)-Cur complexes can enhance antioxidant efficacy, induce M2 macrophage polarization, and alleviate inflammation.^41^ Based on these mechanisms, we designed and fabricated a multifunctional shell-in-shell copper-based coating. The outer shell incorporates a Cur/Cu_2_O heterojunction to facilitate efficient sonodynamic antibacterial activity and disrupt bacterial metabolism, whereas the inner shell is doped with strontium to promote bone tissue regeneration. This multilayered architecture exerts sequential and synergistic effects across antibacterial, antioxidative, and osteogenic processes, holding great promise for overcoming current bottlenecks in the treatment of IAIs (Fig. 1).

## Results

### Synthesis and characterization

**Fig. 2:**
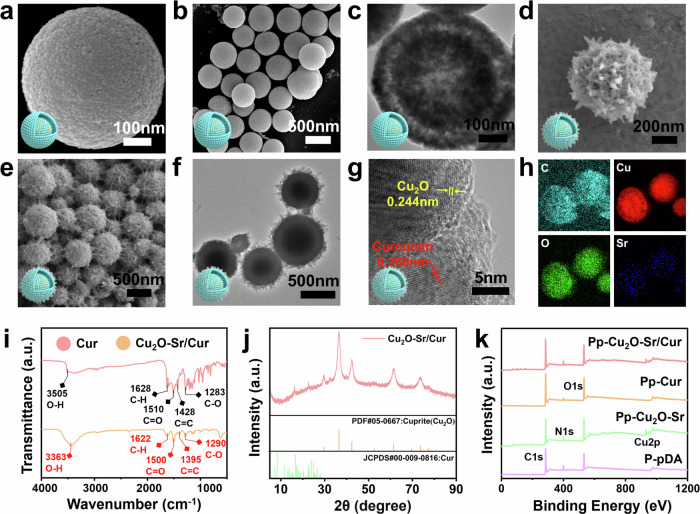
Characterization of nanoparticles. **a**, **b**, **d**, **e** SEM images of shell-in-shell Cu_2_O-Sr (**a**, **b**) and Cu_2_O-Sr/Cur (**d**, **e**). **c**, **f** TEM images of shell-in-shell Cu_2_O-Sr (**c**) and Cu_2_O-Sr/Cur (**f**). **g** HRTEM image of Cu_2_O-Sr/Cur. **h** EDS images showing the elemental composition of Cu_2_O-Sr/Cur. **i** FTIR spectra of Cur and Cu_2_O-Sr/Cur. **j** XRD patterns of Cu_2_O-Sr/Cur. **k** XPS spectra comparing surface chemical states across various samples

Cu_2_O-Sr/Cur was prepared via a one-pot hydrothermal synthesis, with a schematic diagram of the product presented in Supplementary Fig. 1. Briefly, polyvinyl pyrrolidone powder was added to CuSO_4_·5H_2_O and SrCl_2_ solutions, which were reduced by N_2_H_4_·H_2_O to yield single-shell Cu_2_O-Sr (Supplementary Fig. 2a, b). N_2_H_4_·H_2_O was added once again to form an additional nanoshell around the structure of the Cu_2_O-Sr single-shell nanoshell. Cur was subsequently introduced alongside the shell-in-shell Cu_2_O-Sr nanoshell into a DMF solution, followed by thorough stirring and vacuum drying to obtain the desired Cu_2_O-Sr/Cur composite. Without the addition of Cur, the shell-in-shell Cu_2_O-Sr nanoshell possessed a microspherical structure with a rough surface and an average size of 500 nm (Fig. 2a, b). Transmission electron microscopy (TEM) revealed shell-in-shell nanostructures (Fig. 2c). Scanning electron microscopy (SEM) revealed a uniform distribution of Cu-Cur nanodots on the surface of the double-layered Cu_2_O nanospheres (Fig. 2d, e), and the TEM image of the Cu_2_O-Sr/Cur microspheres confirmed the same result (Fig. 2f). The high-resolution TEM (HRTEM) image revealed a compact interface between the Cur nanodots and the Cu_2_O-Sr nanospheres (Fig. 2g). High-resolution lattice fringes measuring 0.244 nm corresponded to the (111) planes of Cu_2_O, while the 0.260 nm spacing matched that of Cur crystals. The Fourier transform infrared spectroscopy (FTIR) analysis of the Cu_2_O-Sr/Cur composites displayed characteristic Cur absorption bands that exhibited a e redshift compared to pure Cur powder (Fig. 2i). The observed redshift likely results from hydrogen bonding or metal–ligand coordination between Cur and the Cu_2_O substrate, which weakens the functional group bond energy and decreases their vibrational frequency. The uniform distributions of C, Cu, O and Sr on the spherical structure, as confirmed by EDS (Fig. 2h, Supplementary Fig. 2c), further validated the successful chelation of Cur. EDS analysis of Cu_2_O-Sr/Cur further revealed that Sr is predominantly localized within the inner shell. The Sr release experiment further confirmed that the coating materials on the scaffold maintained good adhesion stability under US conditions. The rapid accumulation of Sr between days 7 and 14 was attributed to the decomposition of the inner Sr-containing layer, triggered by the acid-induced degradation of the Cu_2_O shell structure. (Supplementary Fig. 2d). The XRD pattern of Cu_2_O-Sr/Cur revealed characteristic peaks of Cu_2_O and Cur, which was consistent with the SAED results and confirmed the success of the material synthesis (Fig. 2j). While the characteristic peaks of Cu_2_O are clearly observed, the signals from Cur appear relatively weak. This can be attributed to the low content and uniform distribution of Cur in the composite.

Polydopamine (pDA) enables strong adhesion via metal coordination and π–π interactions and was employed to immobilize Cu_2_O-Sr/Cur onto the PEKK scaffold.^42,43^ SEM and X-ray photoelectron spectroscopy (XPS) confirmed the successful construction of PEKK-pDA-Cu_2_O-Sr/Cur (Pp-CSC), which was identified by two clear peaks for C 1 s and O 1 s (Supplementary Fig. 2e, Fig. 2K). Supplementary Fig. 2f displays the Cu 2p XPS spectrum, featuring characteristic peaks near 932 and 952 eV, indicative of Cu(I) and Cu(II) species. To evaluate the response to ultrasonic treatment, we examined the stability of the Cu_2_O-Sr/Cur layer deposited on the PEKK surface (Supplementary Fig. 3). In Process A, the scaffolds released both Cu and Cu_2_O-Sr/Cur particles, whereas in Process B, only Cu was released. ICP analysis revealed that the Cu concentrations obtained via both procedures were similar and did not significantly differ from those obtained without the US control group, indicating that the Cu_2_O-Sr/Cur on the PEKK substrate remained stable due to the strong binding properties of pDA.

### Sono-electric and sono-catalytic performance

**Fig. 3:**
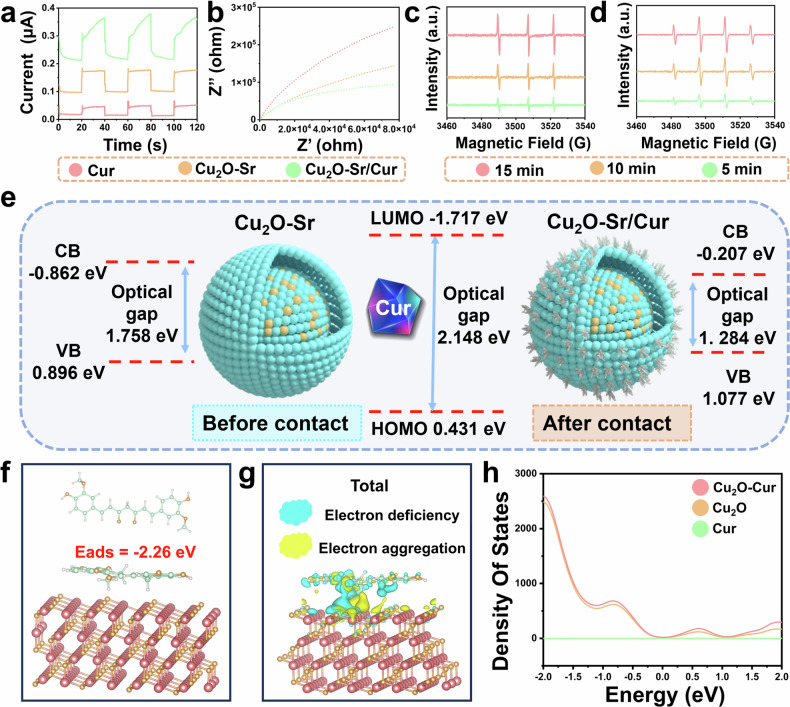
SDT-mediated sonocatalytic capability and DFT calculations of interfacial Engineering. **a** Sonocurrent response of different synthesized materials (200 μg mL^−1^) under US. **b** EIS of different synthesized materials (200 μg mL^−1^) under US. **c**, **d**^1^O_2_ (**c**) and •OH (**d**) obtained from the ESR of Cu_2_O-Sr/Cur (200 μg mL^−1^) for various times under US. **e** Energy band structure of Cu_2_O and Cur pre- and post-interaction. **f** Cu_2_O–Cur adsorption configuration. **g** Differential charge density map at the Cu_2_O–Cur interface (blue: electron loss; yellow: electron gain). **h** DOS spectra of Cu_2_O–Cur interface

As shown in Fig. 3a, after US irradiation, Cu_2_O-Sr/Cur exhibited a significant sonocurrent density, whereas the other groups exhibited only minor fluctuations. These results indicate that the herbal bioheterojunctions (HB-bioHJs) formed by Cu_2_O-Sr/Cur improved electron‒hole separation and facilitated efficient charge transfer at the interface. In addition, electrochemical impedance spectroscopy (EIS) revealed a decreased interface resistance in Cu_2_O-Sr/Cur, indicating that the transfer of the sonocarrier could decrease the charge transfer barrier and facilitate charge transfer (Fig. 3b).

To assess the sonocatalytic enhancement of Cu_2_O-Sr/Cur HB-bioHJs, singlet oxygen (^1^O_2_) and hydroxyl radicals (•OH) generated under US irradiation were quantified via 1,3-diphenylisobenzofuran (DPBF) and methylene blue (MB) probes. Prolonged ultrasonic irradiation and higher Cu_2_O-Sr/Cur concentrations led to a marked reduction in DPBF absorbance, which indicated effective ^1^O_2_ generation (Supplementary Fig. 4a, b). A pronounced decline in MB content was detected following Cu_2_O-Sr/Cur-mediated ultrasonic activation, with both concentration- and time-dependent degradation profiles, which further confirms the efficient generation of •OH. (Supplementary Fig. 4d, e). This performance was notably superior to that of the samples treated with Cu_2_O-Sr (Supplementary Fig. 4c, f). Considering the interference of Cur on the absorbance after dissolution, electron spin resonance (ESR) spectroscopy was applied to evaluate and contrast the ROS production efficiency between Cur and Cu_2_O-Sr/Cur. The results revealed that Cu_2_O-Sr/Cur generated significantly higher levels of ROS than Cur did under identical conditions (Fig. 3c, d, Supplementary Fig. 5a–d). These findings underscore the superior sonodynamic performance of Cu_2_O-Sr/Cur, highlighting its promising potential as a sonosensitizer for antibacterial applications.

### Catalytic mechanism and density function theory calculations

The ultraviolet-visible diffuse reflection spectroscopy (UV‒vis DRS) results for the synthesized materials are presented in Supplementary Fig. 6a. The optical gaps of Cur, Cu_2_O-Sr, and Cu_2_O-Sr/Cur were calculated as 2.148 eV, 1.758 eV, and 1.284 eV, respectively (Supplementary Fig. 6b, c). The valence bands (VBs) of Cu_2_O-Sr and Cu_2_O-Sr/Cur, along with the highest unoccupied molecular orbital (HOMO) of Cur derived from the XPS valence band spectrum, were determined to be 0.896 eV, 1.077 eV and 0.431 eV, respectively (Supplementary Fig. 6d, e). When forming a tight interface, Cu_2_O-Sr/Cur exhibited a low optical gap (Fig. 3e). Therefore, US could activate Cu_2_O-Sr/Cur, increase the utilization of electrons, and further improve its redox capability, resulting in the production of more ROS.

Calculations via Density Functional Theory (DFT) focused on the (111) surfaces of Cu_2_O and Cur were performed to to simulate interfacial carrier transport and elucidate their electronic interactions. As shown in Fig. 3f, the crystal structures of Cu_2_O and Cur were modeled, revealing an adsorption energy of −2.26 eV, which suggests a thermodynamically favorable interaction at the interface. The differential charge density (DCD) after bonding revealed a pronounced electron enrichment on the Cu_2_O surface and marked decrease on the Cur side, supporting the presence of an internal electric field (Fig. 3g). In addition, we measured the distance between the atoms. In the clear charge transfer regions (marked by circles in Supplementary Fig. 7), after the formation of a tight interface between Cur and Cu_2_O, the bond length and bond angle of Cu•••O changed dramatically compared with the corresponding values before contact (Supplementary Table 1). To verify the above speculation, we measured the distance between related atoms (Supplementary Table 2). On the basis of the reported van der Waals radii of H (1.20 Å), O (1.52 Å), and Cu (1.40 Å) atoms, the total van der Waals radii of H•••O and O•••Cu were calculated to be 2.72 and 2.92 Å, respectively. The bond lengths of H•••O and O•••Cu were shorter than the sum of their van der Waals radii, indicating van der Waals interactions. Figure 3h illustrates the density of states (DOS) characteristics at the intimate Cu_2_O–Cur interface. The analysis indicates that electronic states near the Fermi level (EF = 0 eV) are predominantly contributed by Cu_2_O. The observed enhancement in DOS intensity relative to pristine Cu_2_O suggests improved electrical conductivity resulting from interaction with Cur.

### Antimicrobial activity in vitro

**Fig. 4:**
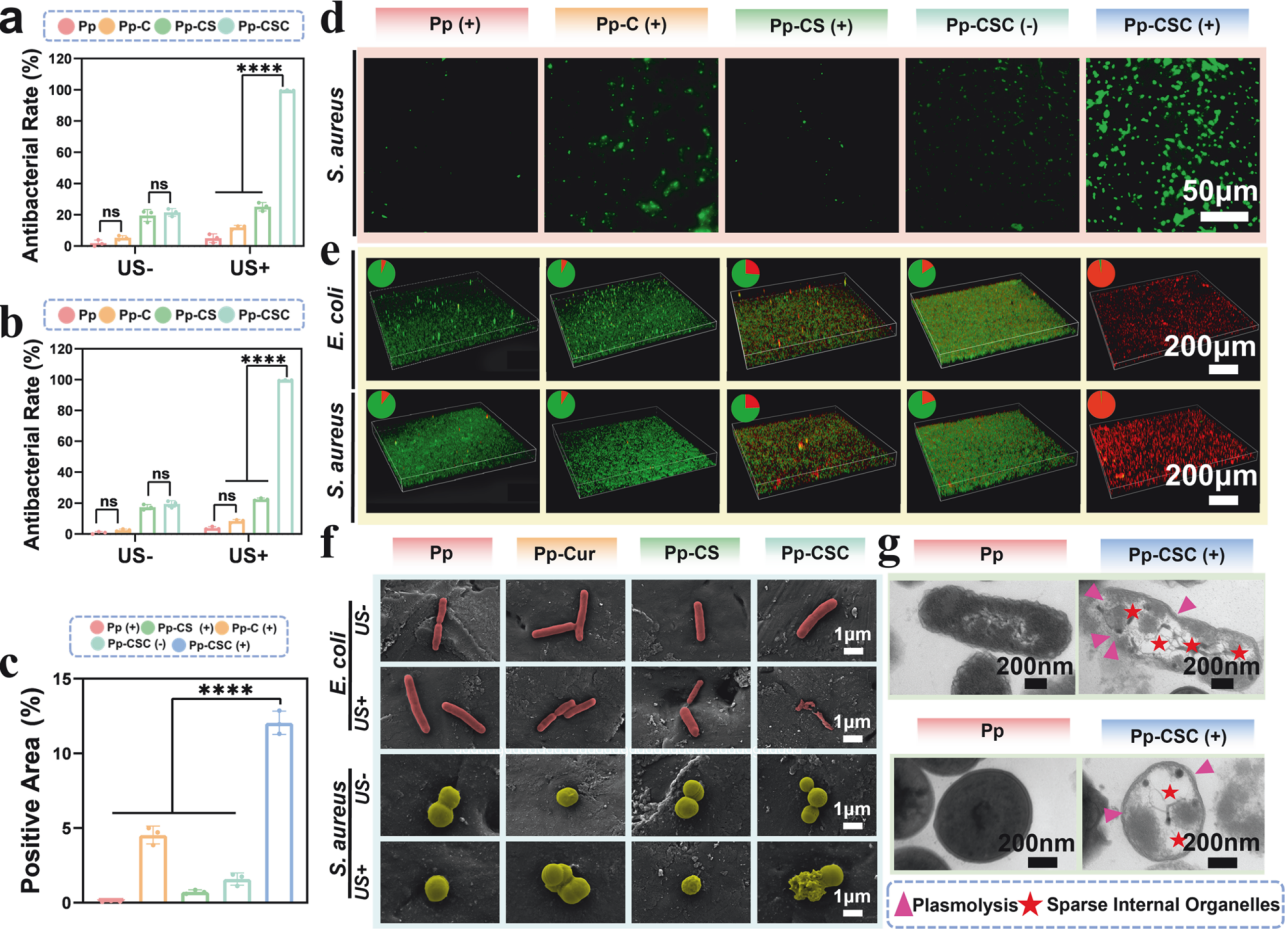
Antibacterial performance in vitro. **a**, **b** Quantification of *S. aureus* (**a**) and *E. coli* (**b**) following various treatments. **c** Quantification of the fluorescence area via DCFH. **d** The fluorescence image of intracellular ROS via DCFH-DA (green) of *S. aureus* after treatment with different scaffolds. **e** Live/dead CLSM of biofilms on scaffolds ±US (**f**) SEM of *S. aureus* and *E. coli* incubated with different scaffolds. **g** TEM of of *S. aureus* and *E. coli*. Violet arrows: plasmolysis; red stars: reduced organelles. Statistical significance among biologically independent samples (*n* = 3) was determined via ANOVA followed by Tukey’s multiple comparisons tests. The data are presented as means ± SDs. Significant differences between groups are indicated as ^*^p < 0.05, ^**^p < 0.01, ^***^p < 0.001, ^****^p < 0.0001, and ns: not significance

Cur, Cu_2_O-Sr, and Cu_2_O-Sr/Cur were immobilized onto PEKK scaffolds via pDA and designated Pp-C, Pp-CS, and Pp-CSC, respectively. Under US stimulation, the viability of *Staphylococcus aureus* (*S. aureus*) and *Escherichia coli* (*E. coli*) decreased negligibly following treatment with PEKK-pDA (Pp). However, Pp-CSC achieved 99.56% antibacterial efficiency against *S. aureus* and 99.43% antibacterial efficiency against *E. coli* under US, suggesting its potential as an antibiotic alternative. Without US, both Pp-CS and Pp-CSC exhibited certain antibacterial activities (Fig. 4a, b, Supplementary Fig. 8). Additionally, LIVE/DEAD fluorescence confirmed the same results mentioned above. (Fig. 4e). To verify the antibacterial effect triggered by SDT, ROS levels were assessed via the fluorescent probe 2′,7′-dichlorofluorescein diacetate (DCFH-DA). Upon US activation, the Pp-CSC group exhibited a nearly fourfold increase in fluorescence compared with baseline, significantly surpassing all other groups, suggesting its superior ROS generating capability (Fig. 4c, d).

To investigate the mechanism underlying the antimicrobial activity of Pp-CSC, we used SEM to observe bacterial morphological changes following different treatments. Notably, Pp-CSC combined with US induced severe surface damage and structural collapse in bacteria (Fig. 4f). Bio-TEM was further used to assess bacterial ultrastructure. In the Pp group, bacterial envelopes showed no signs of disruption, indicating a compact intracellular structure. Conversely, bacteria in the Pp-CSC (US + ) group presented compromised walls and cytoplasmic membranes, accompanied by cytoplasmic leakage of both *S. aureus* and *E. coli* (Fig. 4g). These observations revealed that Pp-CSC-mediated SDT exhibited significant sonocatalytic performance for the eradication of pathogenic microorganisms. After immersion in simulated body fluid (SBF), Pp and Pp-CSC scaffolds were retrieved on days 1, 3, 5, and 7 for coincubation with bacteria, followed by antibacterial evaluation under US stimulation. Although the antibacterial efficacy of Pp-CSC slightly decreased, it still maintained a bactericidal rate of approximately 97.6% against *S. aureus* and *E. coli* (Supplementary Figs. 9 and 10). Additionally, when stored under ambient conditions without treatment, Pp-CSC maintained high antibacterial activity over 1, 3, and 5 weeks (Supplementary Fig. 11). These results collectively demonstrate the excellent antibacterial durability and environmental stability of the Pp-CSC scaffold.

### Underlying antibacterial mechanism

**Fig. 5:**
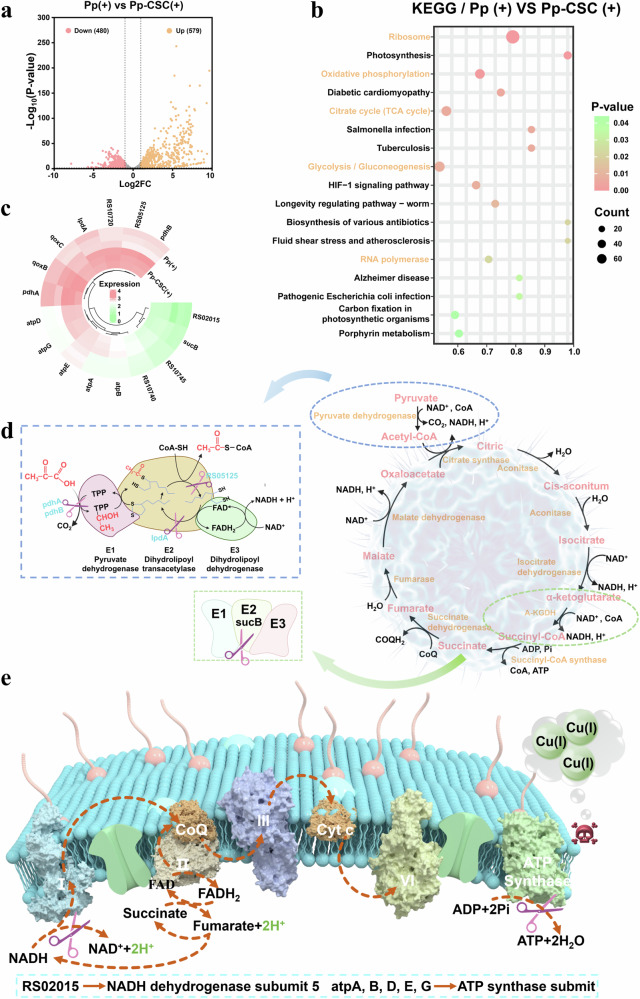
Bacterial transcriptome analysis. **a** Volcano plots illustrating differentially expressed genes. The red and orange dots indicate DEGs whose expression was downregulated and upregulated, respectively. **b** KEGG enrichment analysis of the Pp+US and Pp-CSC + US treatments. **c** Cluster analysis of the effects of genes involved in bacteria related to the TCA cycle and oxidative phosphorylation on expression patterns (**d**) Illustration of the interference mechanism of Pp-CSC (US + ) with the TCA cyles of *S. aureus*. **e** Illustration of the interference mechanism of Pp-CSC (US + ) with the oxidative phosphorylation of *S. aureus*

To comprehensively explore the antimicrobial mechanism of Pp-CSC, transcriptome analysis was conducted on *S. aureus* strains treated with Pp (US + ) or Pp-CSC (US + ). As shown in Fig. 5a, 579 genes showed increased expression while 480 exhibited decreased expression in the Pp-CSC (US + ) group compared with the Pp (US + ) group. Relevant biochemical, metabolic, and signal transduction pathways were identified through Kyoto Encyclopedia of Genes and Genomes (KEGG) enrichment analysis (Fig. 5b). KEGG analysis revealed that Pp-CSC (US + ) primarily affected the following bacteria-related pathways: ribosome, oxidative phosphorylation, the citrate cycle, glycolysis/gluconeogenesis, and RNA polymerase. The observed changes in ribosomal and RNA polymerase-related genes may be attributed to the increased membrane permeability induced by the ROS generated from Pp-CSC under ultrasonic conditions, allowing the compounds to penetrate bacteria, disrupt ribosomes, and inhibit RNA polymerization (Supplementary Fig. 12).

KEGG enrichment revealed that Pp-CSC (US + ) significantly disrupted the citrate cycle (TCA cycle) pathway, suggesting a cuproptosis-like death mechanism. The TCA cycle begins with the entry of acetyl-CoA, which is produced from pyruvate by the pyruvate dehydrogenase complex. This complex has three domains: pyruvate dehydrogenase (E1), dihydrolipoyl transacetylase (E2), and dihydrolipoyl dehydrogenase (E3).^44^ The upregulation of pdhA and pdhB affected the expression of E1, resulting in a decrease in the ability of pyruvate to remove carboxyl groups. The upregulation of RS05125 and lpdA limited the activity of dihydrolipoamide transacetylase and dihydrolipoamide dehydrogenase, disrupting the lipoyllysine cycle and consequently reducing E2 activity. Moreover, we validated these results by examining the effect of Pp-CSC (US + ) on bacterial pyruvate dehydrogenase activity. The enzyme activity of the Pp (US + ) group was three times greater than that of the Pp-CSC (US + ) group (Supplementary Fig. 13a). In addition, the upregulation of sucB gene expression could affect the activity of dihydrolipoamide succinyl transferase, resulting in a decrease in the activity of the α-ketoglutarate dehydrogenase complex, which can affect the TCA cycle (Figs. 5c, 5d). Moreover, genes involved in oxidative phosphorylation (RS02015, atpA, atpB, atpD, atpE, atpG, RS10720, RS10745, and RS10740) were significantly altered (Fig. 5c). The upregulation of RS02015 impaired NADH dehydrogenase (Complex I) activity, whereas the increased expression of genes such as atpA and atpB reduced the activity of ATP synthase (Complex V) activity (Fig. 5e, Supplementary Fig. 13b). Consequently, electron flow and ATP synthesis were hindered, decreasing bacterial ATP levels (Supplementary Fig. 13c).

To elucidate the synergistic antibacterial mechanism driven by ROS-induced bacteriolysis and bacterial cuproptosis‑like death, a series of mechanistic investigations were conducted. Initially, *S. aureus* or *E. coli* were incubated with the US-treated leachate of Pp-CSC for 24 h. The antibacterial rate remained below 50%, indicating that Cu(I) release alone was insufficient to achieve effective bactericidal activity. In addition, bacteria were treated with Pp-CSC under US in the presence of tetrathiomolybdate (TTM), a known copper chelator. Under these conditions, the antibacterial rate increased to 65%, indicating that ROS play a predominant role in bacterial inactivation (Supplementary Fig. 14). Protein leakage assays revealed that Pp-CSC + US caused the most severe cytoplasmic leakage, reflecting extensive damage to bacterial membranes (Supplementary Fig. 15a). In addition, bacterial DNA quantification showed minimal intact DNA in the Pp-CSC (US + ) group, suggesting extensive DNA damage driven by the ROS storm. (Supplementary Fig. 15b). These findings confirm that ROS-triggered bacteriolysis and bacterial cuproptosis‑like death inhibition act in concert to elicit robust synergistic antibacterial effects.

### Cytocompatibility, osteogenic differentiation and osteoclast inhibition

In addition to effectively inactivating bacteria, a good sono-nanoreactor should also minimize harm to normal tissues and promote the healing of bone defects. Therefore, we assessed the viability and proliferation of MC3T3-E1 preosteoblasts. The Pp-CS scaffold exhibited a certain degree of cytotoxicity, which may be attributed to the presence of Cu(I). In contrast, the Pp-CSC scaffold demonstrated favorable cytocompatibility, primarily attributed to the bioactive properties of Cur, which promotes cell proliferation and attenuates oxidative stress (Supplementary Fig. 16a). The comparison between Pp-CS and Pp-CS+Cur further confirmed the significant positive effect of Cur on enhancing cell viability (Supplementary Fig. 16b). Additionally, cellular fluorescence labeling was used to identify the intracellular structures. The results indicated that Pp, Pp-C, Pp-CS, and Pp-CSC presented extended green cytoskeletal structures, confirming that our coating design maintained excellent cytocompatibility (Supplementary Fig. 16c). To further assess cytotoxicity, live/dead staining was performed, and the fluorescence staining results aligned with the abovementioned cytotoxicity data (Supplementary Fig. 16d).

**Fig. 6:**
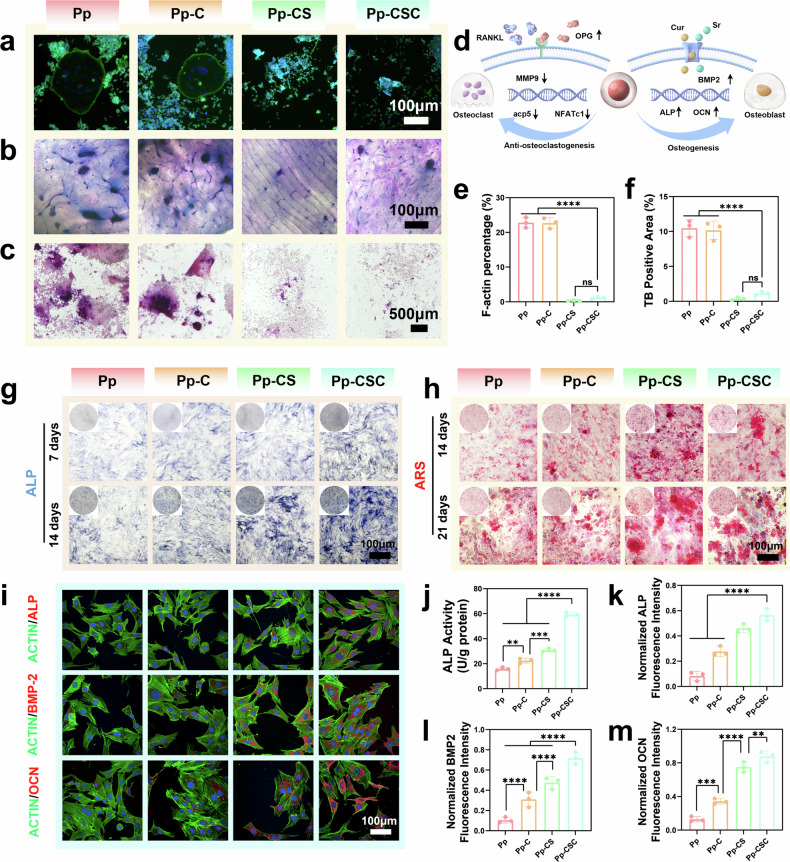
In vitro inhibition of osteoclastogenesis and promotion of osteogenesis. **a** F-actin ring staining of osteoclasts. **b** Bovine bone sections stained with toluidine blue. **c** Osteoclasts labeled with TRAP. **d** Schematic illustration of osteoclast differentiation and intervention. **e** Quantitation of F-actin rings. **f** Quantitation of toluidine blue staining. **g** ALP staining of MC3T3 cells subjected to various treatments at 7 and 14 days. **h** ARS staining of MC3T3 cells subjected to various treatments at 14 and 21 days. **i** Fluorescence microscopy of ALP, OCN and BMP2. **j** Quantification of ALP activity after 14 days. **k**–**m** Semiquantification of immunofluorescence staining for, ALP, BMP2 and OCN. Statistical significance among biologically independent samples (*n* = 3) was determined via ANOVA followed by Tukey’s multiple comparisons tests. The data are presented as the means ± SDs. Significant differences between groups are indicated as ^*^p < 0.05, ^**^p < 0.01, ^***^p < 0.001, ^****^p < 0.0001, and ns: not significant

F-actin staining and bovine bone slices were used to assess the impact of Pp-CSC on osteoclastogenic differentiation. F-actin staining revealed no observable osteoclast formation with no distinct pseudopodia or folded areas on the actin loop in the Pp-CSC and Pp-CS groups (Fig. 6a, e). RAW264.7 cells were cultured on bovine bone slices and stained with toluidine blue, and the stained areas in the Pp-CSC and Pp-CS groups were notably smaller than those in the other groups (Fig. 6b, f). Moreover, osteoclastic enzymatic activity was significantly reduced in both Pp-CSC and Pp-CS, as indicated by the decreased area of TRAP-positive multinucleated osteoclasts (Fig. 6c). These findings demonstrate that Sr incorporated in Pp-CSCs and Pp-CSs effectively suppresses osteoclast differentiation, which is in agreement with prior research.^45,46^

To determine the effect of Sr on the suppression of osteoclastogenesis, PCR analysis of osteoclast-related transcription factors was conducted following the coculture of RAW 264.7 cells with various materials. The results indicated that acp5, NFATc1, and MMP9 were downregulated, whereas OPG was upregulated (Supplementary Fig. 17a–d). This outcome may result from the competitive binding of upregulated OPG to RANK, which inhibits osteoclast differentiation and bone resorption by suppressing downstream transcription factors (Fig. 6d). In terms of osteogenic differentiation, alkaline phosphatase (ALP) and alizarin red S (ARS) assays indicated that Pp-CSC outperformed the other scaffolds in promoting osteogenesis. (Fig. 6g, h, j, Supplementary Fig. 18). Furthermore, Pp-CSC increased the expression levels of osteogenic markers, including ALP, osteocalcin (OCN), and bone morphogenetic protein 2 (BMP2) (Fig. 6i, k, l, m).

### Evaluation of the antioxidative mechanism and anti-inflammatory efficacy

**Fig. 7:**
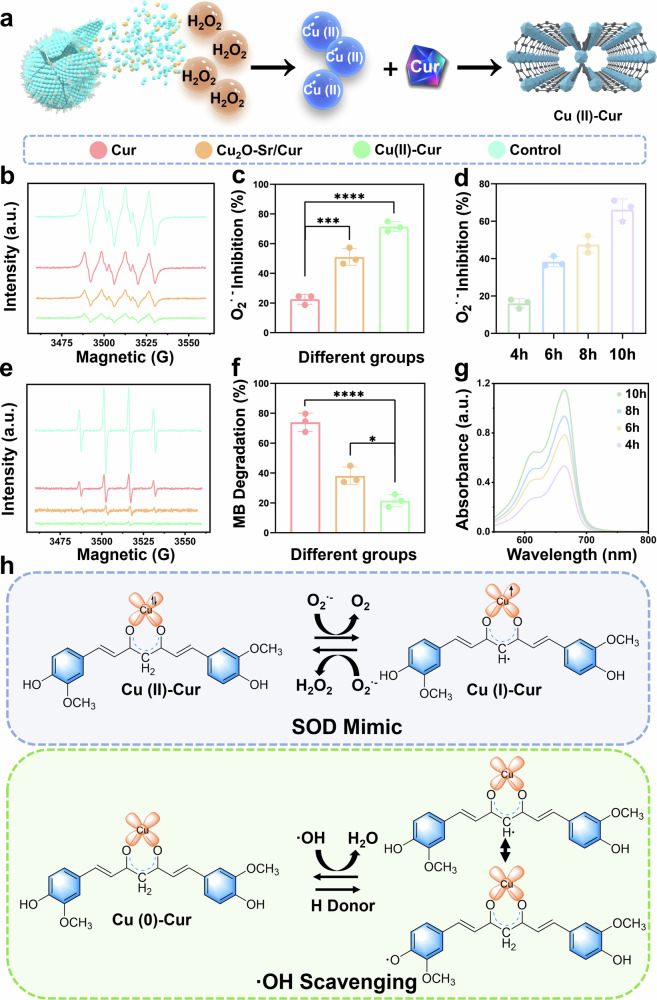
Antioxidation performance and mechanism of Cu_2_O-Sr/Cur. **a** Schematic diagram of the generation of Cu(II)-Cur to improve antioxidation. **b** ESR spectra of the XO + X reaction system after various treatments. **c** O_2_•^-^ inhibition rates of various treatments. **d** Inhibition rate of O_2_•^-^ by Cu_2_O-Sr/Cur after reaction with H_2_O_2_ for different durations. **e** ESR spectra of the Fenton reaction system after various treatments. **f** MB degradation efficiency across treatment groups. **g** UV‒Vis absorption spectra of MB in a Fenton reaction system of Cu_2_O-Sr/Cur + H_2_O_2_ at different times. **h** Chemical mechanism of O_2_•^-^ and •OH scavenging. Statistical significance among biologically independent samples (*n* = 3) was determined via ANOVA followed by Tukey’s multiple comparisons tests. The data are presented as the means ± SDs. Significant differences between groups are indicated as ^*^p < 0.05, ^**^p < 0.01, ^***^p < 0.001, ^****^p < 0.0001, and ns: not significant

In the Cu_2_O-Sr/Cur nanocarrier, Cu_2_O undergoes disproportionation in H_2_O_2_-rich environment, forming Cu(II) and Cu(0), which then combine with Cur to form Cu(II)-Cur (Fig. 7a). The ratios of Cu(I) to Cu(II) in the Cu_2_O-Sr/Cur and Cu_2_O-Sr/Cur + H_2_O_2_ samples were measured via XPS (Supplementary Fig. 19a, b). The peak at 933 ~ 934 eV in the Cu2p3/2 spectra was attributed to Cu(II), along with distinctive Cu(II) shakeup satellite features (938–945 eV). A stronger signal at 932–933 eV was indicative of Cu(I) or Cu(0) species. Notably, the integrated area ratio of Cu(I) to Cu(II) decreased by 14.5% upon exposure to hydrogen peroxide, suggesting that partial oxidation of Cu(I) to Cu(II) occurred on the surface during reaction with hydrogen peroxide. UV‒Vis absorption spectra of the nanostructure in PBS with H_2_O_2_ revealed a new absorption peak (520 nm) with a gradual increase in absorption intensity (Supplementary Fig. 20a), which was consistent with the UV‒Vis spectrum of Cu(II)-Cur (Supplementary Fig. 20b), indicating the gradual formation of Cu(II)-Cur. ESR spectroscopy was employed to examine the O_2_•^-^ scavenging effects after the addition of Cur, Cu_2_O-Sr/Cur + H_2_O_2_, or Cu(II)-Cur. The characteristic signal of O_2_•^-^ (1:1:1:1) was markedly diminished following the introduction of Cu_2_O-Sr/Cur, indicating its effective O_2_•^-^ removal ability. Furthermore, the O_2_•^-^ scavenging ability of Cu_2_O-Sr/Cur was superior to that of Cur (Fig. 7b), which aligns with the results obtained from a superoxide dismutase (SOD) activity assay (Fig. 7c). The O_2_•^-^ scavenging performance of Cu_2_O-Sr/Cur was shown to increase over the reaction time with H_2_O_2_ (Fig. 7d), a phenomenon attributed to the formation of Cu(II)-Cur. Additionally, the ESR spectroscopy results, along with the results of methylene blue (MB) degradation experiments, confirmed that both Cu(II)-Cur and Cu_2_O-Sr/Cur can effectively scavenge •OH (Fig. 7e, f). The UV–Vis absorption spectra further revealed a time-dependent enhancement in the •OH scavenging capacity of Cu_2_O-Sr/Cur during reaction with H_2_O_2_ (Fig. 7g).

Cyclic voltammetry (CV) was performed to investigate the mechanism by which the Cu(II)-Cur complex neutralizes ROS. The CV curve of the Cu(II)-Cur complex exhibited extra oxidation peaks relative to Cur alone, arising from the catalytic function of the Cu that modifies the redox properties of the system (Supplementary Fig. 21a, b). Moreover, the observed cathodic shift in the oxidation peak alongside an anodic shift in the reduction peak reflected enhanced binding affinity between Cu(II) and Cur. On one hand, the Cur can modulate Cu(II) catalytic function, endowing the complex with superoxide dismutase–mimetic activity that efficiently eliminates O_2_•^-^. On the other hand, Cu coordination can perturb the electronic structure of Cur, enhance hydrogen atom transfer and thus facilitate •OH scavenging. (Fig. 7h). Thus, the Cu_2_O-Sr/Cur-mediated self-catalytic regulation strategy enables the production of highly antioxidative Cu(II)-Cur, which supports the antioxidant management of IAIs after sterilization. To evaluate the effects of Cu_2_O-Sr/Cur on macrophage phenotype transition, immunofluorescence staining was employed. Both the Pp-Cu(II)-Cur and The Pp-CSC group exhibited a marked elevation in M2-like macrophages (CD206⁺) alongside a notable decrease in M1-like macrophages (iNO⁺). (Supplementary Fig. 22). These findings suggest that the Cu(II)-Cur generated by Cu_2_O-Sr/Cur promotes macrophage polarization toward the anti-inflammatory and tissue-reparative M2 phenotype, while concurrently suppressing M1-associated inflammatory activation.

### In Vivo Antibacterial Analysis

**Fig. 8:**
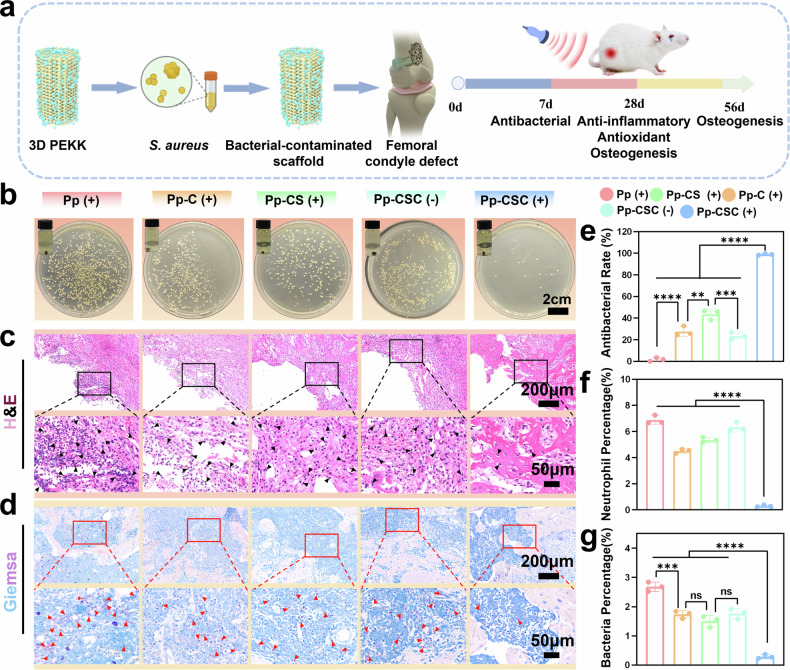
In vivo antibacterial assay (**a**) Diagram of animal experiments involving infected PEKK scaffolds. **b** Images of bacterial colonies and culture turbidity. **c**, **d** H&E (**c**) and Giemsa (**d**) staining images of infected bone tissues around the implants. Black arrows: neutrophils; red arrows: bacteria. **e** Quantification of bacterial viability rates. **f** Semiquantification analysis of neutrophil infiltration in peri-implant infected bone. **g** Semiquantification analysis of bacterial presence in peri-implant infected bone. Statistical significance among biologically independent samples (*n* = 3) was determined via ANOVA followed by Tukey’s multiple comparisons tests. The data are presented as the means ± SDs. Significant differences between groups are indicated as ^*^p < 0.05, ^**^p < 0.01, ^***^p < 0.001, ^****^p < 0.0001, and ns: not significant

A femoral condyle bone infection model was established in SD rats to evaluate the antibiofilm capabilities of the engineered implants (Fig. 8a, Supplementary Fig. 23). After infection induction, US stimulation (1 MHz, 50% duty cycle, 1.5 W cm^-2^) was applied at the implant site. Seven days after surgery, the rats were euthanized, and their femurs harvested for microbiological culture and tissue examination. Visual examination revealed significant secretion and pus around the implants in the Pp (US + ), Pp-C (US + ), Pp-CS (US + ), and Pp-CSC (US-) groups, whereas no secretion or pus was observed in the Pp-CSC (US + ) group, indicating effective sterilization (Supplementary Fig. 24). Colony-forming unit (CFU) analysis of LB agar plates revealed the following results: Pp (US + ) > Pp-C (US + ) ≈ Pp-CSC (US-) > Pp-CS (US + ) > Pp-CSC (US + ), suggesting that US-activated HB-bioHJs significantly expedited elimination of infection in vivo (Fig. 8b, e). Additionally, the OD value of the diluted bacterial solution in the Pp-CSC (US + ) group was the lowest, further demonstrating its superior antibacterial activity (Supplementary Fig. 25). We further validated the antibacterial effect of the materials under US using DiR-labeled *Staphylococcus aureus*. The results revealed that the bacterial fluorescence signal in the Pp-CSC (US + ) group weakened and disappeared within 10 min, demonstrating the superior in vivo antibacterial efficacy of SDT (Supplementary Fig. 26). Hematoxylin and eosin (H&E) and Giemsa staining of the peri-implant bone tissues were performed to assess the infection status posttreatment. H&E staining revealed that the Pp-CSC (US + ) group had the fewest inflammatory cells around the scaffold, whereas the other groups presented significant inflammatory cell infiltration in adjacent bone areas (Fig. 8c, f). In addition, Giemsa staining revealed minimal bacterial presence in the Pp-CSC (US + ) group (Fig. 8d, g). Overall, the results proved that Pp-CSC could effectively combat bacteria under US stimulation and relieve bacteria-related inflammation.

### Bone regeneration efficiency and biosafety In Vivo

**Fig. 9:**
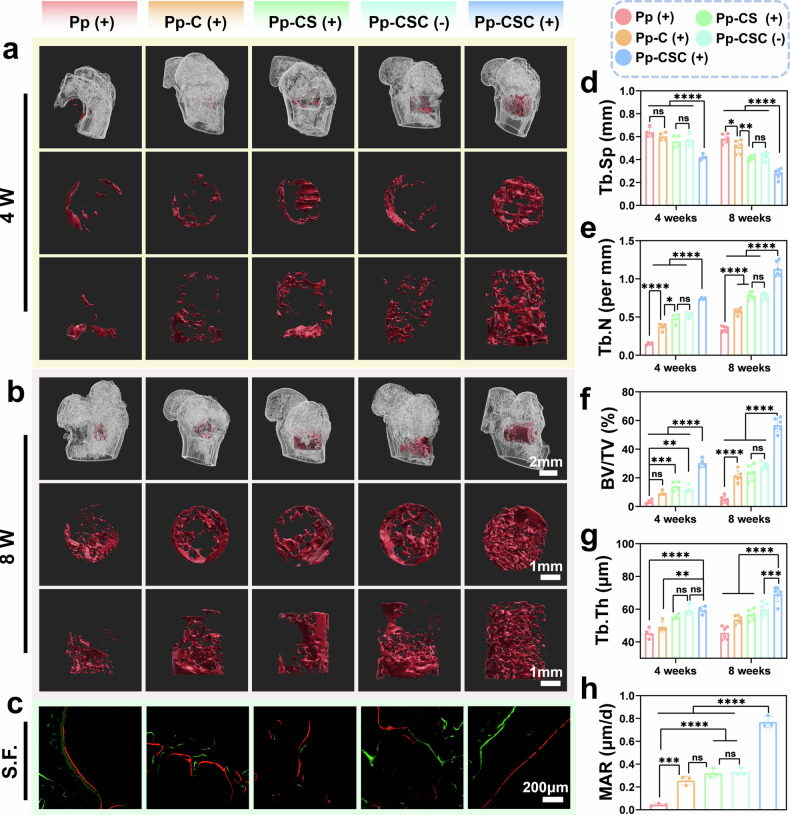
In vivo bone regeneration (**a**, **b**) 3D micro-CT reconstructions of new bone at weeks 4 (**a**) and 8 (**b**) via IMARIS. **b**, **c** New bone labeled by calcitonin (green) and alizarin red (red). **d**, **e**, **f**, **g** Quantification of Tb.Sp (**d**), Tb.N (**e**), BV/TV (**f**), and Tb.Th (**g**). **h** Quantification of the MAR. d–g n = 4 biologically independent samples (4 W), *n* = 6 biologically independent samples (8 W); **h** *n* = 3 biologically independent samples. The significance of biologically independent samples was calculated via ANOVA followed by Tukey’s multiple comparisons test. The data are presented as means ± SDs. Significant differences between groups are indicated as ^*^p < 0.05, ^**^p < 0.01, ^***^p < 0.001, ^****^p < 0.0001, and ns: not significance

Microcomputed tomography (micro-CT) scans were performed on femurs containing implants at 4th and 8th weeks post-implantation to assess new bone formation at the implant interface across treatment groups. IMARIS software was utilized to generate three-dimensional reconstructions of the femoral condyle and bone defect regions, and the results revealed the largest volume of new bone surrounding the scaffold in the Pp-CSC (US + ) group, indicating the superior osteogenic capacity of the engineered implant in vivo (Fig. 9a, b). The other groups exhibited notable peridefect bone loss, primarily due to inflammation and bacterial-induced osteolysis. At the 8th week, new bone in the Pp-CSC (US + ) group effectively filled the scaffold pores. On the basis of micro-CT data, a noticeable reduction in trabecular separation (Tb. Sp) was observed in the Pp-CSC (US + ) group (Fig. 9d). Additionally, other parameters, such as bone volume/total volume (BV/TV), trabecular thickness (Tb. Th), and trabecular number (Tb. N), were highest in the Pp-CSC (US + ) group (Fig. 9e, f, g). To further validate the necessity of the shell-in–shell structure and elucidate the in vivo function of Sr, micro-CT analysis was conducted at week 4 to assess the osteogenic performance of the single-shell Cu_2_O-Sr/Cur (Pp-Single CSC (+) group), shell-in–shell Cu_2_O-Sr/Cur, and Sr-free Cu_2_O/Cur (Pp-Cu_2_O/Cur (+) groups) scaffolds. Compared with the Pp-Single CSC (+) and Pp-Cu_2_O/Cur (+) groups, the Pp-CSC (+) group presented significantly improved bone regeneration, confirming the essential role of the shell-in–shell structure and emphasizing the osteogenic benefits of sustained Sr release from the inner layer (Supplementary Fig. 27). Calcitonin and alizarin red were applied in situ for sequential fluorescent labeling to quantify new bone formation rates. Pp-CSC (US + ) exhibited the largest gap between the green and red fluorescence deposition lines (Fig. 9c). Compared with the other groups, the quantification of the mineral apposition rate (MAR) revealed faster bone deposition in the Pp-CSC (US + ) group (Fig. 9h).

**Fig. 10:**
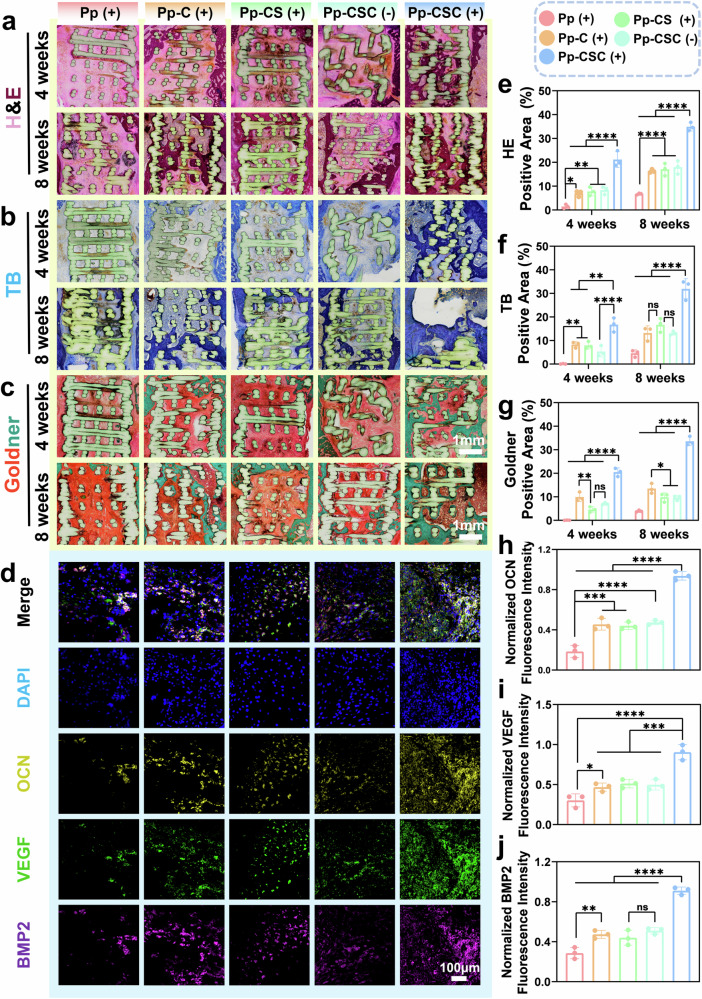
The microstructure and mechanism of new bone in growth. **a**–**c** H&E (**a**), TB (**b**) and Goldner (**c**) staining of the defect area at weeks 4 and 8 after implantation. **d** Immunofluorescence staining of OCN (yellow), VEGF (green) and BMP2 (purple). **e**, **f**, **g** Quantification of (**e**) H&E, (**f**) TB and (**g**) Goldner staining. **h**, **i**, **j** Normalized fluorescence intensity of OCN (**h**), VEGF (**i**) and BMP2 (**j**). Statistical significance among biologically independent samples was calculated via ANOVA followed by Tukey’s multiple comparisons test. The data are presented as means ± SDs. Significant differences between groups are indicated as ^*^p < 0.05, ^**^p < 0.01, ^***^p < 0.001, ^****^p < 0.0001, and ns: not significance

At 4th and 8th weeks post-implantation, nondecalcified scaffold-bone tissues were processed for H&E, toluidine blue, and Goldner’s trichrome staining. (Fig. 10a–c). The quantitative assessment revealed that among these groups, the Pp-CSC (US + ) group presented the greatest amount of new bone tissue (Fig. 10e–g). These staining results indicated that new bone primarily formed around the scaffolds and gradually penetrated inside the porous scaffolds. Immunofluorescence with tyramide signal amplification (TSA) for VEGF, BMP-2, and OCN was performed to evaluate bone and blood vessel ingrowth at the defect site (Fig. 10d). As shown in Fig. 10h–j, OCN, VEGF and BMP-2 were substantially upregulated in the Pp-CSC (US + ) group compared with the other groups. This finding indicated enhanced osteogenesis and vascularization, demonstrating its excellent ability to promote bone formation. In addition, tartrate-resistant acid phosphatase (TRAP) staining of the tissue surrounding the scaffold revealed the fewest neutrophils in the Pp-CSC (US + ) group, further confirming the effective inhibition of osteoclast formation by Sr (Supplementary Fig. 28). Immunohistochemical analysis demonstrated a significant reduction in tumor necrosis factor-α (TNF-α), interleukin 1β (IL-1β), and interleukin 6 (IL-6) expression in the Pp-CSC (US + ) group, indicating effective bacterial clearance and attenuated inflammatory response that promoted enhanced bone healing in vivo. (Supplementary Fig. 29). Routine blood tests and H&E staining (heart, liver, spleen, lung, and kidney) revealed favorable indicators of Pp-CSCs, confirming their satisfactory biosafety and minimal systemic toxicity. (Supplementary Figs. 30 and 31). Skin temperature changes before and after US exposure were monitored via an infrared thermometer (Supplementary Fig. 32), and the variation was insufficient to cause any damage.

## Discussion

This study presents a novel ultrasound-responsive herbal bioheterojunction (HB-bioHJ) coating with an ingeniously designed shell-in-shell architecture, rationally engineered to orchestrate a spatiotemporal sequence of antibacterial activity, immunomodulation, and osteogenesis. This multifunctional therapeutic paradigm is substantiated by comprehensive physicochemical analyses, in-depth mechanistic investigations, and rigorous in vivo assessments. Physicochemical characterizations validated the successful fabrication of the shell-in-shell nanoarchitecture, where curcumin (Cur) was loaded onto the outer shell for antibacterial functionality, and strontium ions were enriched in the inner shell to promote osteogenesis. This deliberate spatial segregation of functional components provides a robust foundation for spatiotemporally controlled, stage-specific therapeutic interventions, enabling the sequential orchestration of antibacterial, immunomodulatory, and osteogenic effects.

At the early phase of infection treatment, the outer shell of the Cu_2_O-Cur heterojunction efficiently generates reactive oxygen species (ROS) in response to ultrasound (US) stimulation, serving as the primary antibacterial effector. The precisely engineered interface between Cu_2_O and Cur markedly facilitates electron–hole separation, thereby boosting ROS production beyond the capacities of either component alone. These ROS exert multifaceted antibacterial effects by directly compromising bacterial membranes and nucleic acids, while simultaneously increasing membrane permeability to facilitate the enhanced penetration of therapeutic agents. Notably, transcriptomic analyses demonstrated that ROS produced by Pp-CSC under ultrasound exposure disrupted critical biological processes including ribosomal function, RNA polymerase activity, and various metabolic pathways. Collectively, these findings underscore the potent bactericidal efficacy of ROS in breaching the protective barriers of bacterial biofilms and combating multidrug-resistant strains, highlighting the promising potential of ultrasound activated HB-bioHJ systems in addressing refractory implant-associated infections.

Although the ROS generated by the material upon ultrasonic activation can efficiently eradicate the majority of bacterial, a minor fraction may persist within microenvironments,^47^ thereby retaining the capacity for recolonization and infection relapse. Therefore, implementing a sustained antibacterial strategy post-ultrasound cessation is imperative to guarantee continuous bacterial inactivation and the successful management of IAI. Recent advances in tumor biology have provided valuable insights into this issue. Cu(I) ions specifically target lipoylated enzymes, including dihydrolipoamide S-acetyltransferase (DLAT), disrupting the tricarboxylic acid (TCA) cycle, inducing proteotoxic stress, and ultimately causing cellular metabolic collapse. Our experimental data further confirmed that Cu(I) can induce a cuproptosis-like death in bacteria. The release of Cu(I) impairs the function of the pyruvate dehydrogenase complex and disrupts oxidative phosphorylation, resulting in significant ATP depletion and bacterial energy crisis. The combined effects of ROS-mediated bacterial lysis and metabolic disruption through cuproptosis-like pathways establish a robust synergistic antibacterial mechanism, achieving remarkable bactericidal efficiencies of up to 99.56% against *Staphylococcus aureus* and 99.43% against *Escherichia coli*. This dual-modality approach represents a significant advancement over conventional SDT, which is often limited by the short diffusion distance and ephemeral lifespan of ROS within biological environments. In our platform, the ultrasound-activated heterojunction not only enhances local ROS generation but also facilitates prolonged intracellular disruption through precise metal ion regulation, effectively overcoming the inherent limitations associated with SDT monotherapy.

Following the effective bacterial clearance, the HB-bioHJ coating continues to exhibit therapeutic functionality. Within the acidic and H_2_O_2_-enriched microenvironment, Cu(I) ions undergo disproportionation reactions to yield Cu(II), which subsequently coordinates with Cur to form stable Cu(II)-Cur metal–polyphenol complexes. These metal–polyphenol complexes effectively mimic the catalytic activities of antioxidant enzymes, including superoxide dismutase and hydroxyl radical scavengers, thereby exhibiting potent antioxidative and anti-inflammatory properties. This self-catalytic regulatory mechanism not only mitigates ROS-induced oxidative tissue damage but also actively promotes the resolution of inflammation‒an often-overlooked therapeutic aspect in conventional antimicrobial coatings. In addition, the Cu(II)-Cur complexes have been demonstrated to modulate the local immune microenvironment by promoting macrophage polarization toward the anti-inflammatory M2 phenotype, concurrently suppressing the pro-inflammatory M1 activation. This immunomodulatory effect is consistent with recent findings,^48^ indicating that M2-dominant macrophage populations play a crucial role in orchestrating angiogenesis and osteogenesis throughout the bone repair process. By actively remodeling the immune milieu following infection, the coating facilitates a more seamless transition from the antibacterial phase to the regenerative phase, thereby promoting enhanced tissue repair and regeneration.

Importantly, the inner shell enriched with Sr contributes to long-term osteointegration. Sr release inhibits osteoclastogenesis via the RANKL/OPG axis while concurrently promoting osteoblast differentiation through the upregulation of osteogenic transcription factors such as BMP-2 and osteocalcin (OCN). Our in vivo experimental results further validated that this dual mechanism significantly increased trabecular bone thickness and number, facilitating successful new bone formation and integration with the scaffold. Compared with single-shell or Sr-free counterparts, the shell-in-shell architecture enables precise spatiotemporal regulation of Sr ion release, thus achieving temporal coordination of early-stage infection control and late-stage bone regeneration.

The multifunctionality of the HB-bioHJ coating clearly distinguishes it from existing nanoplatforms. Most SDT-based antibacterial systems focus solely on microbicidal efficacy while neglecting the equally important processes of immune regulation.^49,50^ In contrast, our design embraces an all-in-one strategy that integrates self-adaptive, stimuli-responsive features with natural polyphenol coordination chemistry. This approach leverages the intrinsic physicochemical properties of the materials rather than relying on external additives, thereby enhancing biosafety, translational feasibility, and structural stability within complex physiological milieus. Despite these promising results, several limitations and challenges remain to be addressed. First, although the coating exhibited excellent antibacterial and osteogenic efficacy in small animal models, its long-term safety profile and functional performance in large animal models remain to be thoroughly investigated. Second, while the current study focused on PEKK scaffolds, the adhesion, mechanical compatibility, and durability of the coating on other clinically relevant substrates, such as titanium alloys or ceramics, warrant further comprehensive evaluation. Third, ultrasound activation parameters may require optimization tailored to different anatomical sites, particularly for deep tissue implants, where acoustic attenuation and safety thresholds must be rigorously controlled. In summary, this study introduces an ultrasound-activated coating that combines Cu_2_O-mediated cuproptosis-like bacterial killing, Cu_2_O-Cur-assisted sonodynamic effects, Sr-mediated osteoregulation, and Cu-Cur complex-driven antioxidative immune modulation. The sequential therapeutic model addresses key challenges in treating implant-associated infections by not only eradicating bacteria but also orchestrating the tissue microenvironment toward regeneration. Given its modular design, stimuli responsiveness, and multiple therapeutic pathways, the HB-bioHJ platform holds strong promise for broader clinical applications in orthopedic and dental implants and represents a breakthrough in the progressive evolution of biomaterials.

## Materials and methods

### Materials

Unless otherwise indicated, all chemicals purchased from commercial sources were used without additional purification. Cur (C_21_H_20_O_6_), CuSO_4_·5H_2_O, SrCl_2_, C_4_H_6_CuO_4_·H_2_O were obtained from Yuanye (Shanghai, China). Unless specified otherwise. All other chemicals were sourced from Baoxin Biotechnology (Chengdu, China), unless otherwise noted.

### Synthesis of shell in shell Cu_2_O-Sr

Dissolve 75 mg of CuSO_4_·5H_2_O and 7 mg of SrCl_2_ in 30 mL deionized water, add 300 mg of PVP, and stir at 300 rpm until dissolved. Add 15 μL of 80% hydrazine (N_2_H_4_·H_2_O) and observe the formation of an orange suspension after 15 seconds. Centrifuge, discard the supernatant, and redissolve the precipitate in 75 mg CuSO_4_·5H_2_O, 30 mL water, and 300 mg PVP. Stir until dissolved, then add 15 μL of hydrazine. Following centrifugation, samples were subjected to three washes and subsequently freeze-dried overnight.

### Synthesis of shell in shell Cu_2_O-Sr/Cur

In a mixture of 10 mL DMF and 600 μL deionized water, 15 mg of Cu_2_O-Sr was uniformly dispersed. Ultrasonication and continuous stirring were performed to ensure full dispersion of the material. Separately, Cur (3 mg) was dissolved in 6 mL of DMF and then added dropwise to the Cu_2_O-Sr suspension. The resulting mixture was stirred for an additional 30 minutes. The reaction proceeded under argon at 120 °C in an oil bath for 30 min with stirring, then cooled naturally. Following centrifugation, samples were subjected to three washes and dried under vacuum at 60 °C.

### Preparation of Pp-CSC scaffold

The PEKK scaffolds were produced using fused deposition modeling (FDM) in a 3D printing process. Cylindrical scaffolds (3 × 4 mm for in vivo; 10 × 1 mm for in vitro) were designed using Materialise 3-Matic software. Predefined PEKK scaffolds were fabricated by layer-wise extrusion of medical-grade filaments at 200 °C using a 3D printer. PEKK scaffolds were coated with polydopamine (PEKK-pDA, Pp) by stirring in 3 mg mL^−1^ DA-HCl solution (Tris-HCl, 10 mM, pH=8.5) for 24 h. Solutions with different material concentrations were prepared as required, and PEKK-pDA scaffolds were immersed in them for 24 hours to obtain concentration-adjusted material-loaded scaffolds. Based on the weight difference before and after nanoparticle loading and ICP, each PEKK scaffold was estimated to retain approximately 30.7 μg of Cu₂O-Sr/Cur.

### Characterization

SEM (ZEISS Gemini 300, Germany, EHT = 3.00 kV, WD = 7.60 mm) was used to observe the surface morphologies. TEM (HT7800, Hitachi, Japan) was used to obtain high-resolution images and lattice fringe information at 80 kV. Surface elemental composition, chemical states, and valence band structures were characterized by XPS (K-Alpha, Thermo Fisher Scientific) using Al Kα radiation (hv=1486.6 eV). Functional groups were identified by recording FTIR spectra across wavelengths between 500 and 4000 cm^−1^, with 4 cm^−1^ spectral resolution Crystal structures were examined by XRD using an Ultima IV diffractometer (Rigaku, Japan) with Cu Kα radiation (40 kV, 110 mA).

### Detection of ROS in vitro

Samples were mixed with DPBF (Aladdin) or MB (Aladdin) solutions and subjected to US treatment to assess singlet oxygen (^1^O_2_) and •OH radicals. UV-Vis spectroscopy (UV-5200, METASH) recorded DPBF (300–500 nm) and MB (500–700 nm) absorption changes pre- and post-US treatment. ESR spectroscopy (JES-FA200, JEOL) was employed to identify ROS species. Singlet oxygen (^1^O_2_) was trapped using TEMP (50 mM), and hydroxyl radicals (•OH) were detected with DMPO (0.1 mM).

### Density function theory calculation

Vienna Ab Initio Simulation Package (VASP) was used for all DFT simulations, employing the GGA-PBE exchange-correlation functional. Valence electrons were represented by plane waves with a 450 eV cutoff. The Brillouin zone was sampled with a centered 2×2×1 mesh. A 20 Å vacuum prevented slab interactions. Geometry optimizations continued until energy and force thresholds of 1×10^-5 ^eV and 0.03 eV Å^−1^ were satisfied.

The adsorption energy was considered as follows:

$${{\rm{E}}}_{{\rm{ads}}}={{\rm{E}}}_{* {\rm{Cur}}-}{{\rm{E}}}_{{\rm{Cu}}2{\rm{O}}-}{{\rm{E}}}_{{\rm{Cur}}}$$

While E_Cu2O_ and E_Cur_ is the energy of Cu_2_O and Cur, and E_*Cur_ is the energy of Cur adsorbed at Cu_2_O.

### Sono electrochemical measurements

Electrochemical analyses were conducted on a CHI660E system with a three-electrode system. The electrolyte was 50 mL of 0.5 M Na_2_SO_4_ solution. The mixture consisted of 4 mg sample combined with 500 μL deionized water, 500 μL ethanol, and 80 μL Nafion solution. A 150 μL aliquot was drop-cast onto FTO glass, dried, forming a 1×1 cm film. Acoustic measurements were conducted using an ultrasonic device.

### Antibacterial assessment in vitro

*Staphylococcus aureus* (ATCC 25923) and *Escherichia coli* (ATCC 25922) were obtained from Chengdu Haoyi Biotechnology. The bacteria were propagated in LB medium, sterilized by autoclaving, prepared with 10 g tryptone, 10 g NaCl, and 5 g yeast extract per liter.

#### Spread-plate method

Different scaffolds were co-cultured with bacterial suspensions (1 × 10^7^ CFU/mL) in 1 mL sterile tubes. The ultrasound (US) group was stimulated for 10 minutes, while the non-ultrasound group received no treatment (1 MHz frequency, 1.5 W cm^-2^ intensity, 50% duty cycle). Following treatment, bacterial were plated on agar plates and incubated at 37°C for 24 hours to quantify colony-forming units (CFU). Three parallel samples were made for each group.

#### Bacterial morphology

Diluted Staphylococcus aureus and Escherichia coli bacteria solution (10^6^ CFU mL^−1^) were added to the PEKK samples for a total of 8 h, and then US group were exposed to US for 10 min. Following fixation in 4% formaldehyde for 2 hours and subsequent PBS (pH=7.0) rinsing, samples underwent dehydration via a graded ethanol series (30%, 50%, 70%,80%, 90%, 100%) for 10 minutes per step. Samples were then air-dried prior to SEM analysis. (ZEISS Gemini 300, Germany, EHT = 3.00 kV, WD = 8.30 mm). Bacteria subjected to different treatments were collected by low-temperature high-speed centrifugation, embedded, and then sectioned. The sections were treated with uranyl acetate and lead citrate for contrast enhancement., placed on copper grids, and imaged via TEM (JEM-1400FLASH, JEOL, Japan).

#### LIVE/DEAD stain

Scaffolds were co-cultured with 1 mL of bacteria in the logarithmic growth phase in a 37 °C shaking incubator, with the culture medium refreshed every 12 hours. On the fifth day, bacterial viability was assessed via Live/Dead staining (Thermo Fisher, USA), and visualized via confocal laser scanning microscopy (CLSM; N-SIM S, Nikon, Japan) at excitation/emission wavelengths of 488/556 nm.

#### Intracellular ROS detection

Scaffolds were co-cultured with 1 mL of bacteria in the logarithmic growth phase for 4 hours, followed by ROS assay kit (Beyotime, China) and visualization by CLSM.

#### Protein leakage and intact DNA

Genomic DNA damage following various treatments was evaluated by extracting bacterial DNA with a commercial purification kit (Beyotime, China). After centrifugation at 5000 × g for 5 minutes at 4 °C, DNA was isolated from the pellet according to the kit protocol and quantified via UV–vis spectroscopy. In parallel, The BCA method (Beyotime, China) was applied to measure protein content in the supernatant, and optical density was determined using a microplate reader.

### Transcriptome analysis

*S. aureus* (ATCC 25923) was cultured with Pp-Cu_2_O-Sr/Cur and exposed to US stimulation. The experiment was conducted in triplicate for both a control group (Pp (+)-1, Pp (+)-2, Pp (+)-3) and an experimental group subjected to Pp-Cu_2_O-Sr/Cur and US treatment (Pp-CSC ( + )-1, Pp-CSC ( + )-2, Pp-CSC ( + )-3) (CSC: Cu_2_O-Sr/Cur), maintaining identical conditions for all replicates. RNA was isolated in its entirety, and cDNA libraries were prepared following the protocol provided by the manufacturer. Subsequently, sequencing was performed at high throughput using the Illumina HiSeq system (Majorbio, China). Differential gene expression analysis was conducted using DESeq2 (v1.24.0), setting the criteria at |log2FC | ≥ 1 and a p-value < 0.05. Differentially expressed genes were functionally annotated and their pathways analyzed using the KEGG database (http://www.genome.jp/kegg/). KOBAS facilitated pathway enrichment, with Fisher’s exact test applied to determine significance across groups.

### Biocompatibility assessment in vitro

MC3T3-E1 and RAW 264.7 cells (Cell Bank, Chinese Academy of Sciences, China) were cultured in α-MEM or high-glucose DMEM (Gibco), respectively, both supplemented with 10% FBS and 1% penicillin-streptomycin. FBS for RAW 264.7 cells were heat-inactivated at 56 °C for 40 min. All cultures contained 1% penicillin-streptomycin. Cells were incubated at 37 °C with 5% CO₂ for biocompatibility, osteogenesis, and polarization assays.

#### Cell proliferation assay

MC3T3-E1 cells (2×10^5^ cells/mL) were seeded (1 mL per well) onto various samples in 24-well plates. After 3 days, their viability was gauged using a live/dead cell double staining kit (CA1630, Solarbio, China) and then analyzed with a fluorescence microscope (IX73, OLYMPUS, Japan). MC3T3-E1 cells (2×10^4^ cells/mL) were seeded (100 μL per well) into each well of a 96-well plate and co-cultured with various scaffolds. CCK-8 assay (Beyotime, China) was conducted on days 1, 3, and 5 by incubating cells with 10 μL reagent per well at room temperature for 2 h. Absorbance was subsequently recorded using a Multiskan SkyHigh microplate reader (Thermo Fisher Scientific, USA).

#### Cell spreading assay

After seeding MC3T3-E1 cells (2×10⁵ cells/mL) at 1 mL per well on various substrates in 24-well plates and culturing for 3 days, 4% paraformaldehyde solution was applied to the cells to fix them for 2 hours. Permeabilization was carried out using PBS with 0.1% Triton X-100 (Sigma, USA) for 30 min. Phalloidin and DAPI (Solarbio, China) were used to stain the cytoskeleton and nuclei, and fluorescence imaging was conducted using CLSM.

### Osteogenic differentiation

Various samples were seeded with MC3T3-E1 cells at a density of 1×10^5^ cells/mL in 24-well plates (1 mL per well) (1 mL per well). After 1 day of incubation, the existing medium was removed, and new medium supplemented with 50 μg/mL ascorbic acid, 10 mM β-glycerophosphate, and 10 nM dexamethasone was added. (all from Sigma, USA). On the 7th and 14th day, ALP enzyme activity was evaluated employing a BCIP/NBT kit sourced from Beyotime, China, with further quantification on 14th day by an alkaline phosphatase assay kit. Mineralization was visualized by Alizarin Red S staining on days 14 and 21, and the stained matrix was semi-quantified through ImageJ software. MC3T3-E1 cells (2×10^4^ cells/mL) were seeded (1 mL per well) onto various samples in 96-well plates. The culture medium was exchanged with osteogenic medium every other day for a specified period. For the immunofluorescence analysis of Alkaline Phosphatase (ALP), Bone Morphogenetic Protein 2 (BMP2), and osteocalcin (OCN), cells from each group were treated with specific primary antibodies: rabbit anti-ALP (1:200, Abcam, USA), rabbit anti-BMP2 (1:200, Abcam, USA), and rabbit anti-OCN (1:200, Abcam, USA). A donkey-derived anti-rabbit secondary antibody conjugated with Alexa Fluor 594 (1:500, Invitrogen, USA) was employed.The cytoskeleton was labeled using FITC-labeled phalloidin (Solarbio, China), while nuclei were stained with DAPI. Imaging was conducted using a high-content analysis and screening platform (ThermoFisher, USA).

### Osteoclast experiment

#### F-actin ring and TRAP staining

RAW 264.7 cells (4×10^5^ cells/mL) were seeded on scaffolds in 24-well plates and cultured for 24 hours. RANKL (50 ng/mL, SinoBiologica, China) was added to the control groups, while untreated cells served as the control. The medium was changed on Days 3, 5, and 6. At day 7 post-seeding, the cells were gently rinsed with PBS and immobilized by incubation in 4% paraformaldehyde. Subsequent membrane permeabilization was achieved with 0.25% Triton X-100 prior to staining of the F-actin cytoskeletal structures. The F-actin ring was visualized using Actin-Tracker Green, and DAPI was used to stain the nuclei. Fluorescence microscopy was employed to capture images of the stained cells. Cells were cultured as previously described. TRAP working solution (Solarbio, China) was then applied for 1 hour under dark conditions, followed by rinsing with distilled water. Osteoclasts were defined as multinucleated cells with at least three nuclei and were assessed via light microscopy.

#### Toluidine blue staining and SEM of bovine bone slice

Bone slices were placed into 96-well plates, followed by seeding of RAW 264.7 cells (2×10^3^ cells/well) and incubation for 24 h. Cells were grouped and treated as described in the F-actin ring analysis section. The medium was refreshed on days 3, 5, 7, 8, 9, and 10. On day 11, bone slices were rinsed with 0.25 M ammonia solution in an ultrasonic bath, dehydrated through a graded ethanol series, stained with 1% toluidine blue, and rinsed with PBS. Staining was observed by light microscopy. Surface morphology of bovine bone slices was examined via scanning electron microscopy (SEM).

#### Quantitative real-time PCR (qRT-PCR)

Osteoblast-and osteoclast-specific gene expression, including OPG (osteoblasts) and Acp5, MMP-9, NFATc1 (osteoclasts), was evaluated according to the protocol described above. The reverse transcription of RNA into cDNA was carried out utilizing Hifair III 1st Strand cDNA Synthesis SuperMix for qPCR, in accordance with the supplier’s guidelines. The synthesized cDNA served as the template for real-time PCR conducted on a Bio-Rad RT-PCR instrument. Expression analysis was carried out using β-actin for normalization, and the corresponding primer sequences are listed in Supplementary Table 3.

### Antioxidation performance and mechanism

The •OH and •O_2_^-^ inhibition rates of Cur, Cu_2_O-Sr/Cur, and Cu (II)-Cur were measured by MB degradation and SOD assay at pH 6.5. ESR spectroscopy was performed with DMPO/BMPO as the spin trap. Electrochemical analysis of antioxidant mechanisms was conducted using a three-electrode setup. Cyclic voltammetry (CV) was conducted in PBS (pH 6.5) containing Cur, Cu2O-Sr/Cur, and Cu (II)-Cur (120 μM), scanning potentials from +0.8 V to -0.5 V at 50 mV/s. The electrolyte was purged with argon for 10 minutes before measurements.

### Polarization of macrophages

RAW 264.7 cells (2×10^3^ cells/mL) were plated in 96-well plates for 24 hours. Cells were treated with 10 μg mL^−1^ LPS (L4391, Sigma, USA) and 100 μM H_2_O_2_ in the presence of various experimental conditions for another 24-hour period. Primary antibodies targeting CD206 (rabbit, 1:200, Abcam, USA) and iNOS (rat, 1:200, Abcam, USA) were applied to cells, followed by incubation with Alexa Fluor 594 donkey anti-rabbit and Alexa Fluor 488 donkey anti-rat secondary antibodies at 1:500 dilution (Invitrogen, USA). After DAPI counterstaining of nuclei, fluorescence imaging was performed with a high-content screening system (ThermoFisher Scientific, USA).

### In vivo experiments

Animal procedures received ethical approval from the West China Hospital Laboratory Animal Committee and were carried out in strict accordance with institutional guidelines. Two-month-old male Sprague-Dawley rats, weighing between 200 and 220 g, were provided by Beijing Huafukang Bioscience Cojnc. SD rats (*n* = 13 per group) were randomly assigned to 5 groups: Pp (+), Pp-C (+), Pp-CS (+), Pp-CSC, Pp-CSC (+). All groups except Pp-CSC received US treatment. To establish an implant-associated infection model, PEKK (3 mm × 4 mm) were incubated in a *S. aureus* (ATCC 25923, Chengdu Haoyi Biotechnology) suspension (1 × 10^6^ CFU mL^−1^) for 1 day. An orthopedic defect (3 mm × 4 mm) was drilled vertically into the lateral condyle of the rat femur, and the bacteria-laden implants were bilaterally inserted into the defect sites Postoperative US treatment was administered (DJO Chattanooga, Model 2776, USA). At weeks 1, 4, and 8 post-implantation, 3, 4, and 6 rats were sacrificed, respectively. In addition, three rats were allocated to the Pp-Single CSC (+) group and three to Pp-Cu_2_O/Cur (+) group to evaluate its osteogenic potential at week 4. No animals died or were excluded from the analysis during the entire experimental period.

#### Antibacterial activity in vivo

From postoperative days 1 to 6, the rats received ultrasound (US) stimulation under gas anesthesia, using parameters of 1 MHz, 1.0 W cm^−2^, 50% duty cycle for 10 minutes per session. Rats were sacrificed 7 days after surgery, and the extracted implants were immediately placed into PBS-filled tubes. To assess infections related to the implants, the harvested samples were subjected to serial dilution, plated onto LB agar, and incubated for 1 day. Adjacent bone tissue was excised, decalcified, and performed H&E and Giemsa staining.

#### Micro-CT analysis

Quantum GX Micro CT (PerkinElmer, USA) was employed to scan the femoral condyle and evaluate the volume and microstructural characteristics of the newly formed bone surrounding the implant. Scanning was conducted at 80 kV, 45 mA, with a 6 mm × 6 mm scanning area, 4 μm slice thickness, and a total scan time of 4 minutes. Imaris 9.9 was employed to reconstruct CT data into three-dimensional representations. Quantitative bone morphometric analysis was conducted using Skyscan NRecon and CTAN software, focusing on indices such as bone volume (BV), BV/TV ratio, number of trabeculae (Tb.N), trabecular thickness (Tb.Th), and separation distance (Tb.Sp).

#### Sequential fluorescent labelling

At 2 and 6 weeks following surgery, rats received intraperitoneal injections of calcineurin (20 g kg^−1^) and alizarin red (30 g kg^−1^). At week 8, the animals were euthanized, and both the implants and adjacent bones were preserved and sectioned, and examined using a confocal laser scanning microscope (CLSM).

#### Histology and Immunohistochemistry

Prior to decalcification, histological slices were made parallel to the implant’s longitudinal axis around the undecalcified femoral condyle. Bone samples from rats without implants at 8 weeks were processed for immunohistochemistry (IL-6, TNF-α, IL-1β) and immunofluorescence (VEGF, BMP-2, OCN). TRAP staining was used to identify osteoclasts and activated macrophages. Tissue sections were analyzed with the VS200 scanner (Olympus, Japan), and image semi-quantification was carried out via ImageJ software.

### Statistics analysis

Data are expressed as means ± standards deviation (SD), with error bars representing SD. Statistical analyses were conducted using one-way ANOVA followed by Tukey’s post hoc test. Significance levels were defined as ^*^P < 0.05, ^**^P < 0.01, ^***^P < 0.001, and ^****^P < 0.0001.

## Supplementary information

Ultrasound activated herbal bio-heterojunctions for self-catalytic regulation and bacterial cuproptosis-like death in the treatment of implant infection

## Data Availability

Supporting data for this study are included within the Article and Supplementary Information.
