# Supplementary material for: Ultrasound activated herbal bio-heterojunctions for self-catalytic regulation and bacterial cuproptosis-like death in the treatment of implant infection
Source: Signal Transduct Target Ther. 2025 Sep 19;10:303. doi: 10.1038/s41392-025-02388-4 (PMC12446443; doi:10.1038/s41392-025-02388-4)
Supplement: Supplementary file 1 — Ultrasound activated herbal bio-heterojunctions for self-catalytic regulation and bacterial cuproptosis-like death in the treatment of implant infection [file 41392_2025_2388_MOESM1_ESM.docx]

Supplementary Materials for

**Ultrasound activated herbal bio-heterojunctions for self-catalytic regulation and bacterial cuproptosis-like death in the treatment of implant infection**

*Yan Yue ^a,1^, Shuoyuan Li ^a,1^, Qiang Su ^a,1^, Xufeng Wan ^a^, Qiaochu Li ^c^, Hong Xu ^a^, Fuyuan Zheng ^a^, Yangming Zhang ^a^, Le Tong ^d^, Jian Cao ^a^, Long Zhao ^a^, Xiaoting Chen ^e^, Qi Li ^a^, Yi Zeng ^a^, Haoyang Wang ^a^, Yi Deng ^b, *^ Zongke Zhou ^a, *^ Duan Wang ^a, *^*

* Corresponding authors.

E-mail addresses: Wang, D.(wangduan_bone@163.com), Zhou, Z. (zongke@126.com), Deng, Y.(dengyibandeng@scu.edu.cn)

**This PDF file includes：**

Figures. S1 to S32

Tables. S1 to S3

**1. Figures of supporting information**

**
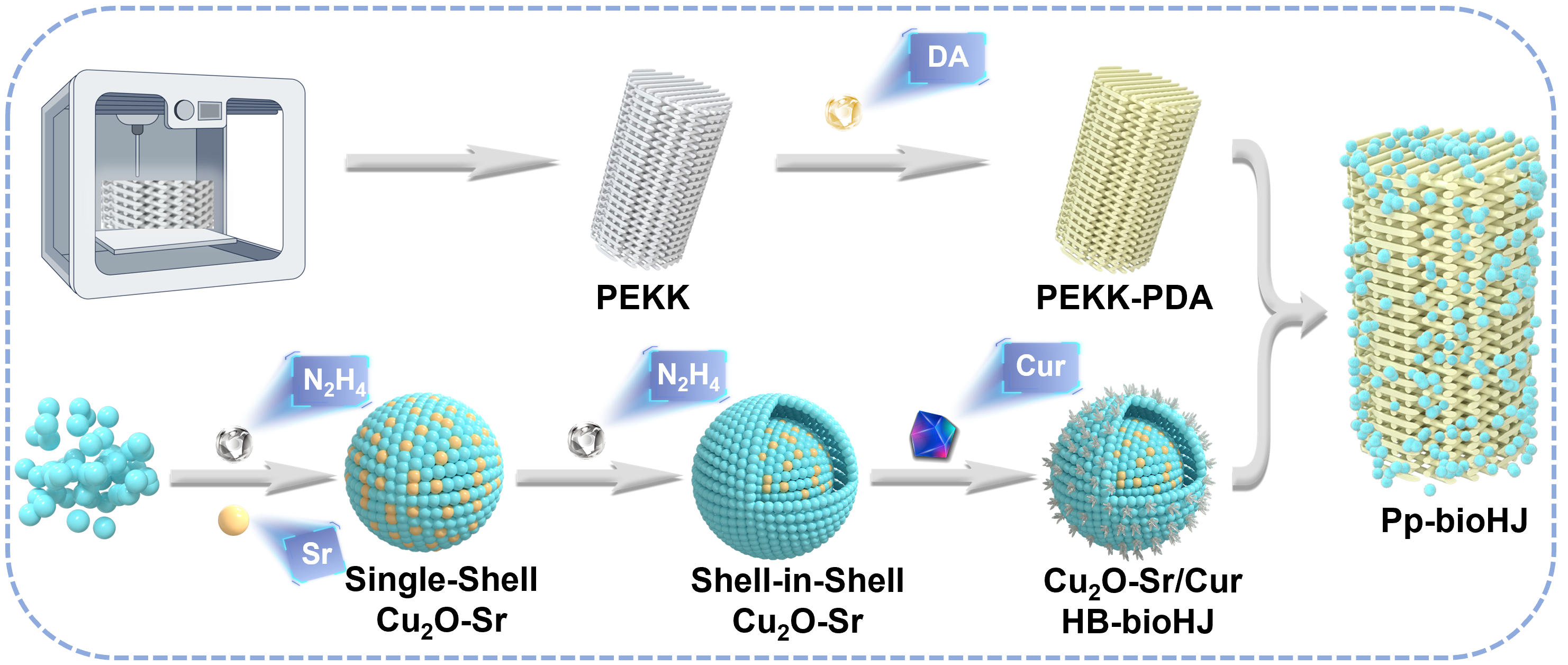
**

**Figure S1.** Schematic illustration of the synthetic process of Pp-bioHJ.


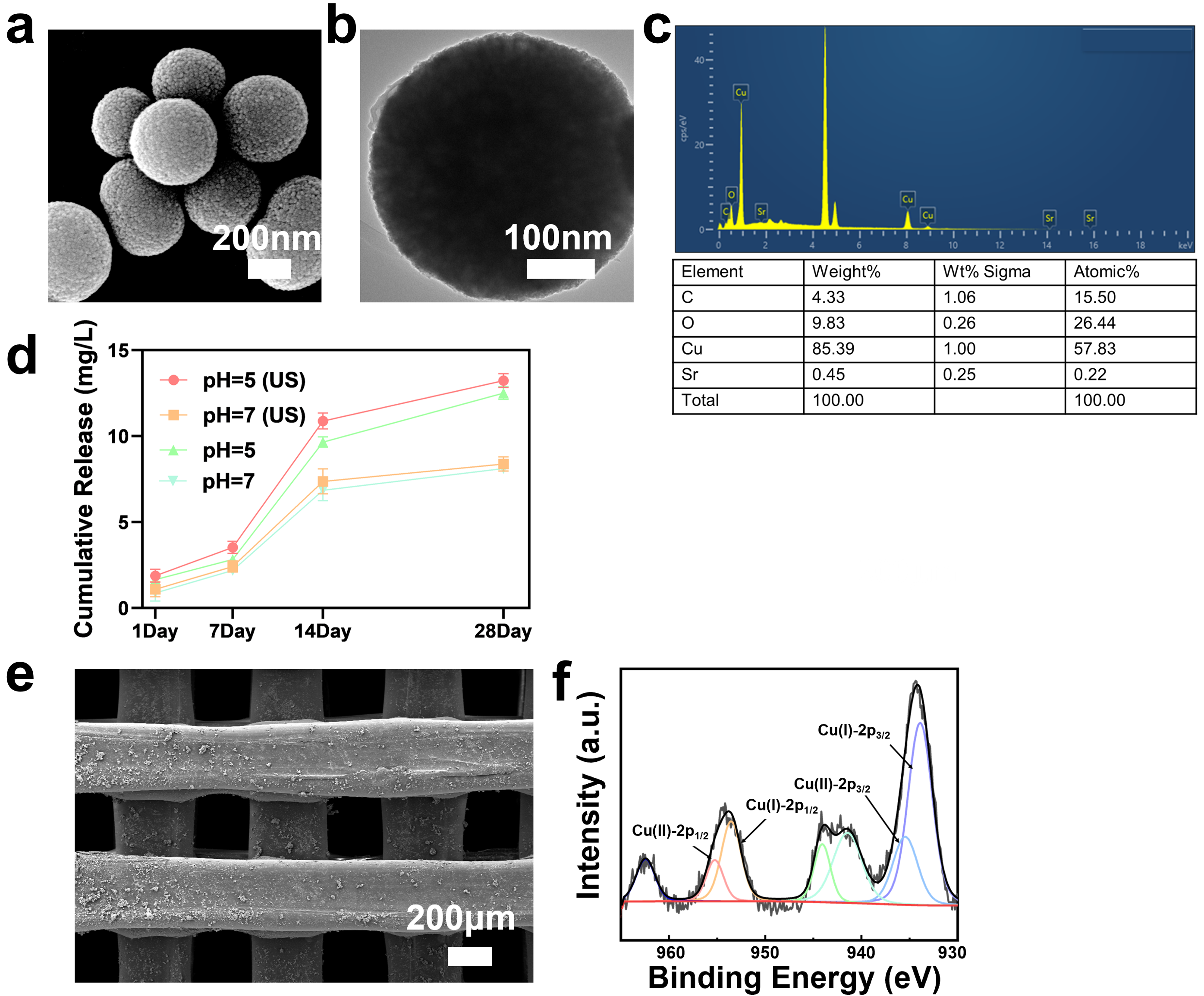


**Figure S2. (a)**SEM image of single-shell Cu_2_O-Sr. **(b)**TEM image of single-shell Cu_2_O-Sr. **(c)** Ratio of the content of different elements in the Cu_2_O-Sr/Cur. **(d)** The release amount of Sr at different time points. **(e)**SEM image of Pp-bioHj. **(f)** Corresponding high-resolution images of Cu 2p in the XPS spectra acquired from Cu_2_O-Sr/Cur.

**
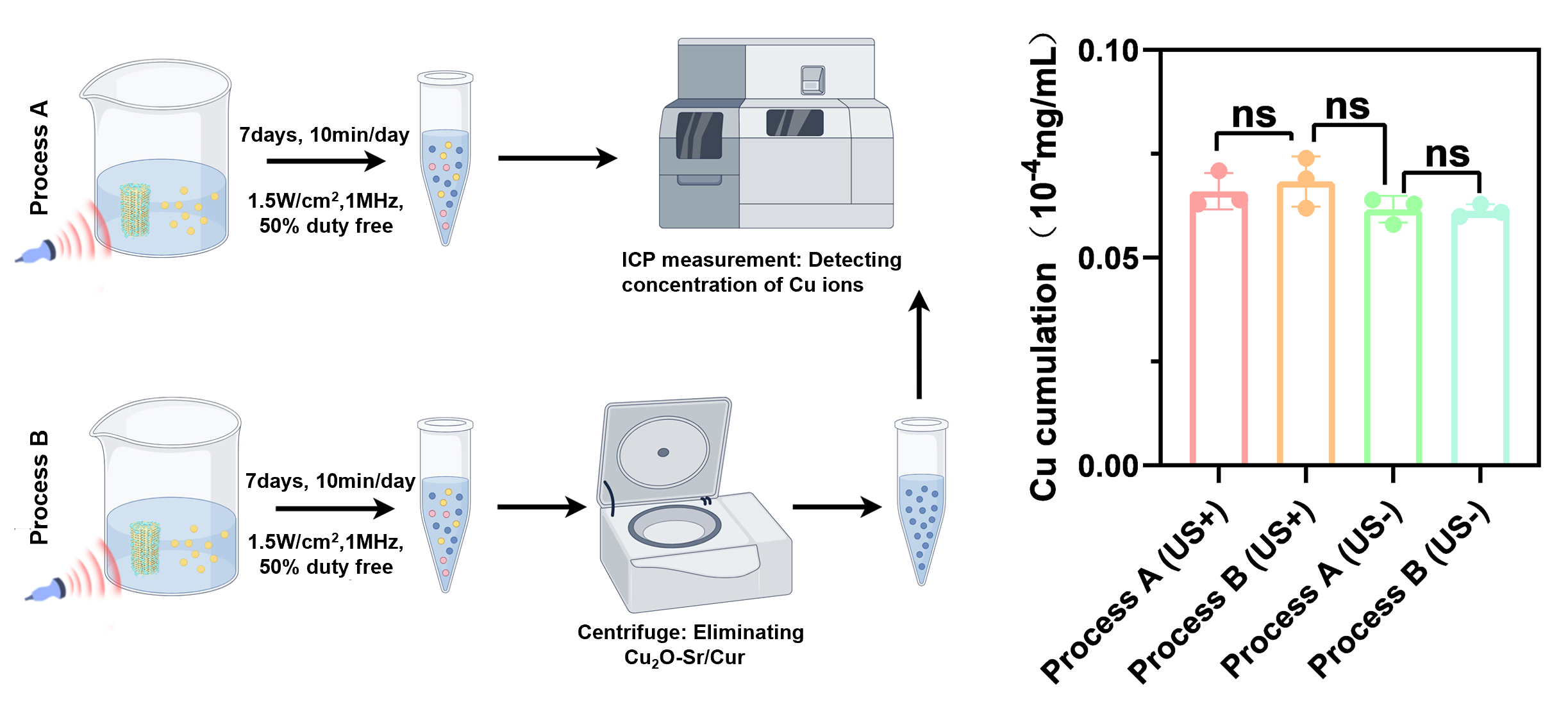
**

**Figure S3.** Cu released from Pp- Cu_2_O-Sr/Cur scaffold with different treatments by ICP. The significance of biologically independent samples (n=3) was calculated by ANOVA followed by Tukey’s multiple comparisons. Data were presented as means ± SDs. (Created by Figdraw.)

**
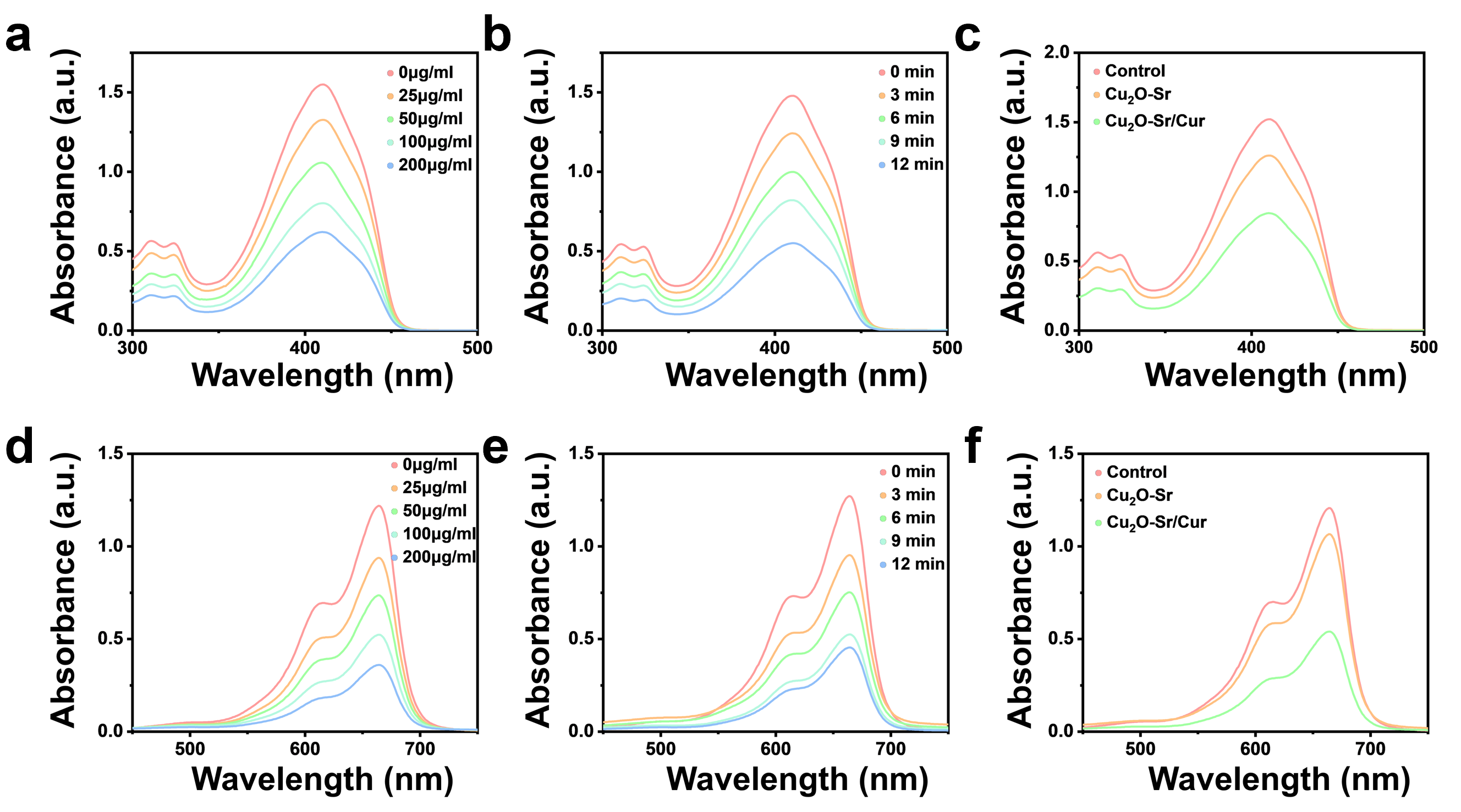
**

**Figure S4. Sonocatalytic Performance. (a)** UV–vis absorption spectra of DPBF for ^1^O_2_ detection of Cu_2_O-Sr/Cur (1.5w cm^-2^, 10min). **(b)** UV–vis absorption spectra of DPBF for ^1^O_2_ detection of Cu_2_O-Sr/Cur (200μg ml^-1^) after different times of ultrasonication. **(c)** UV–vis absorption spectra of DPBF for ^1^O_2_ detection of Cu_2_O-Sr and Cu_2_O-Sr/Cur (1.5w cm^-2^, 10min, 200μg ml^-1^). **(d)** UV–vis absorption spectra of MB for •OH detection of Cu_2_O-Sr/Cur (1.5w cm^-2^, 10min). **(e)** UV–vis absorption spectra of MB for •OH detection of Cu_2_O-Sr/Cur (200μg ml^-1^) after different times of ultrasonication. **(f)** UV–vis absorption spectra of MB for •OH detection of Cu_2_O-Sr andCu_2_O-Sr/Cur (1.5w cm^-2^, 10min, 200μg ml^-1^).

**
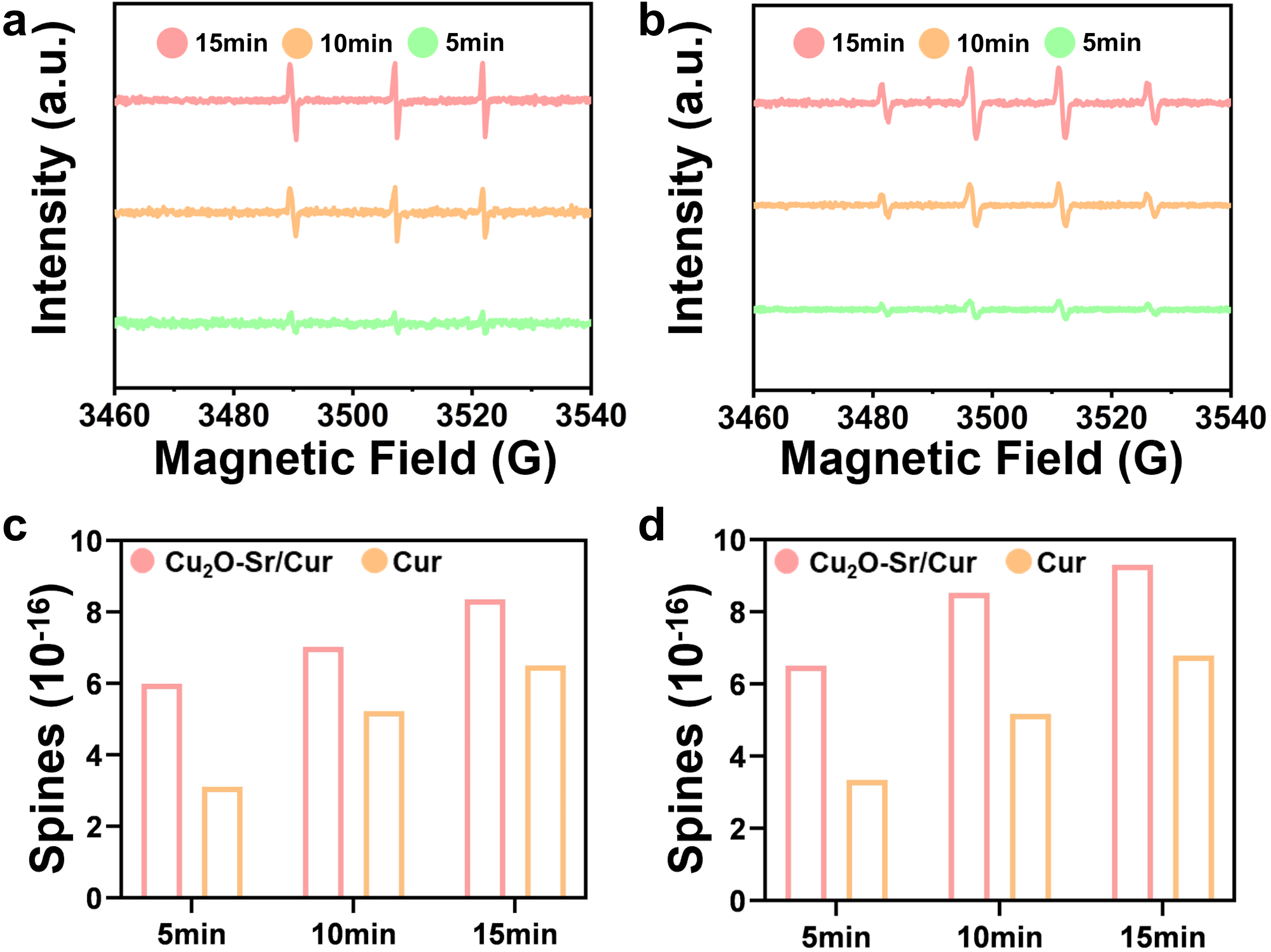
**

**Figure S5.** (**a, b**) ^1^O_2_ (a) and •OH (b) obtained from ESR of Cur (200 μg mL^-1^) for various times under US. (**c**) The ESR quantitative results of ^1^O_2_ generated Cu_2_O-Sr/Cur and Cur. (**d**) The ESR quantitative results of •OH generated Cu_2_O-Sr/Cur and Cur.

**
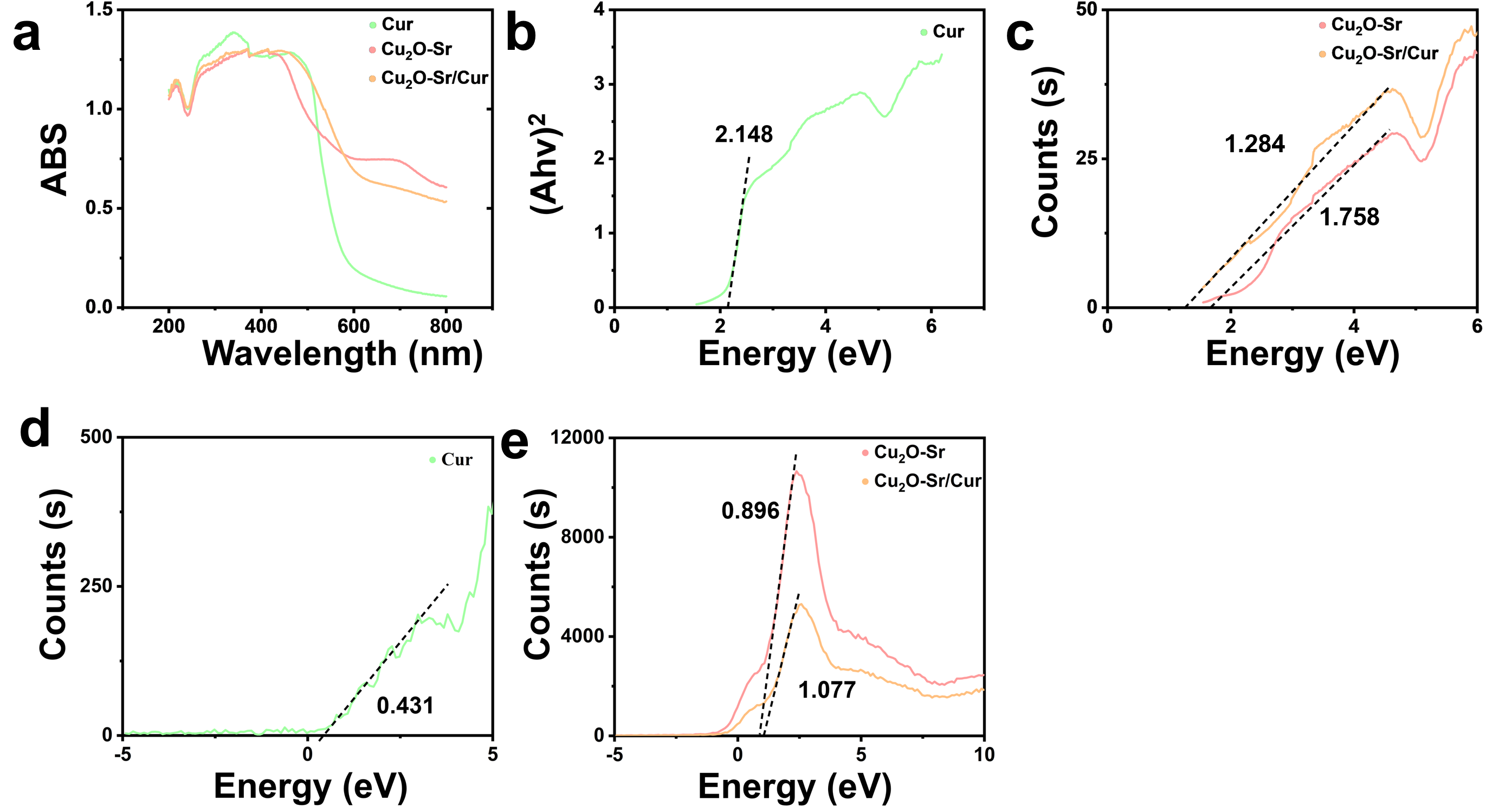
**

**Figure S6.** **(a)** The UV-Vis DRS of Cur and Cu_2_O-Sr, Cu_2_O-Sr/Cur. **(b, c)** The tauc curves of Cur **(b)** and Cu_2_O-Sr, Cu_2_O-Sr/Cur **(c)**. **(d, e)** The valence band of Cur **(d)** and Cu_2_O-Sr, Cu_2_O-Sr/Cur **(e)** calculated from the XPS valence band spectrum.


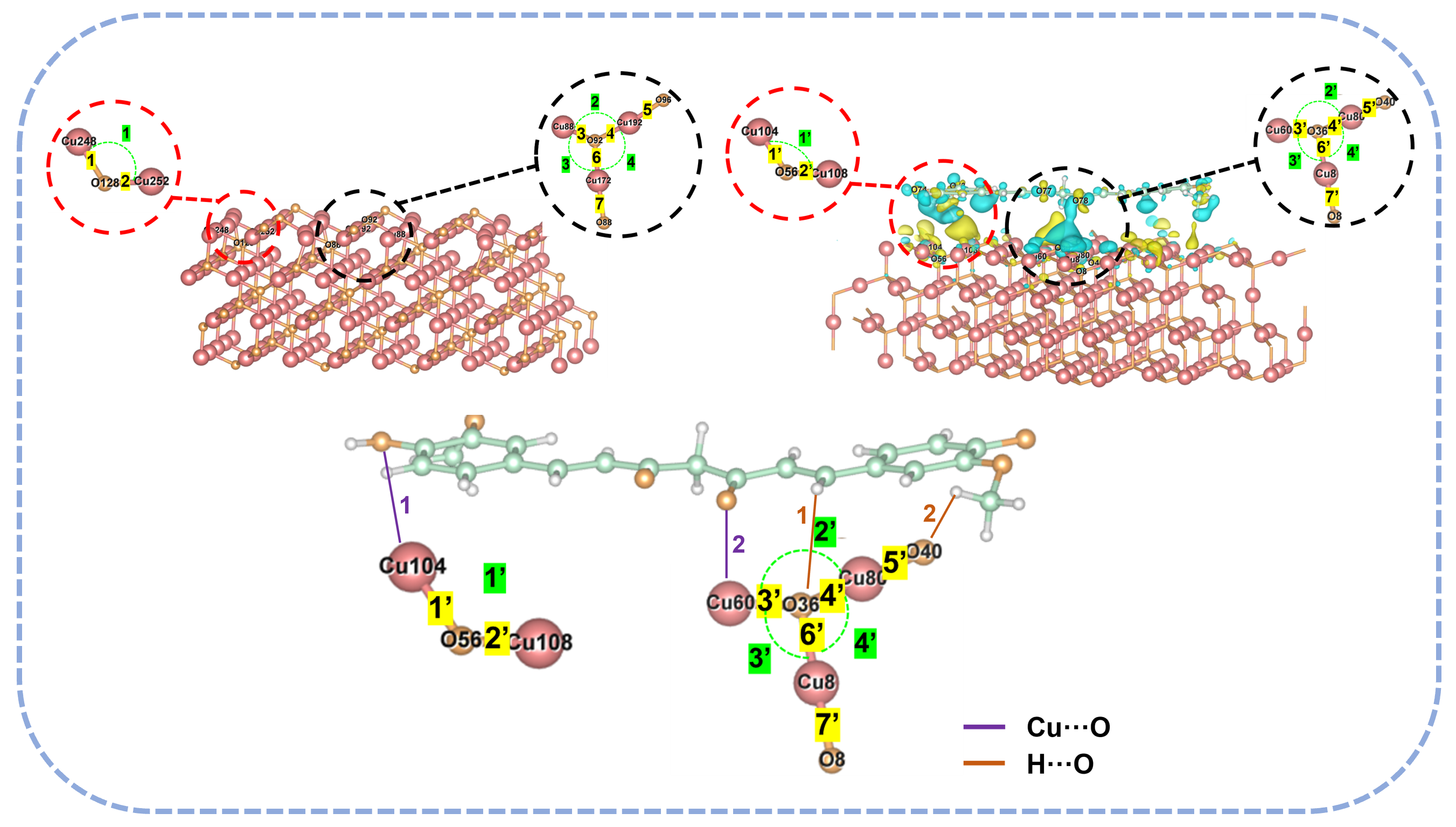


**Figure S7.** The changes in bond lengths and bond angles before and after the formation of the tight interface


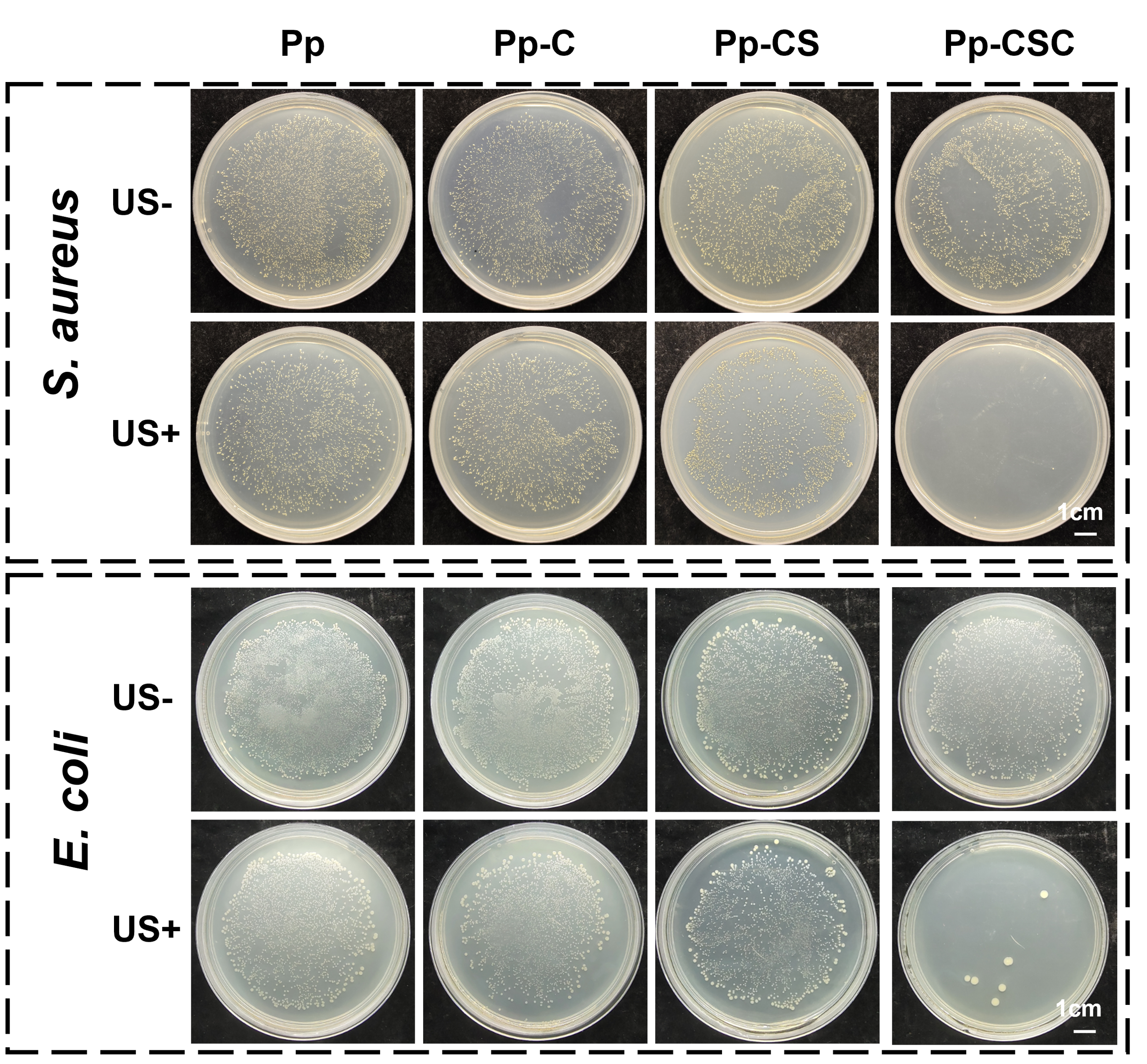


**Figure S8.** Spread plate of *S. aureus* and *E. coli* following various treatments.


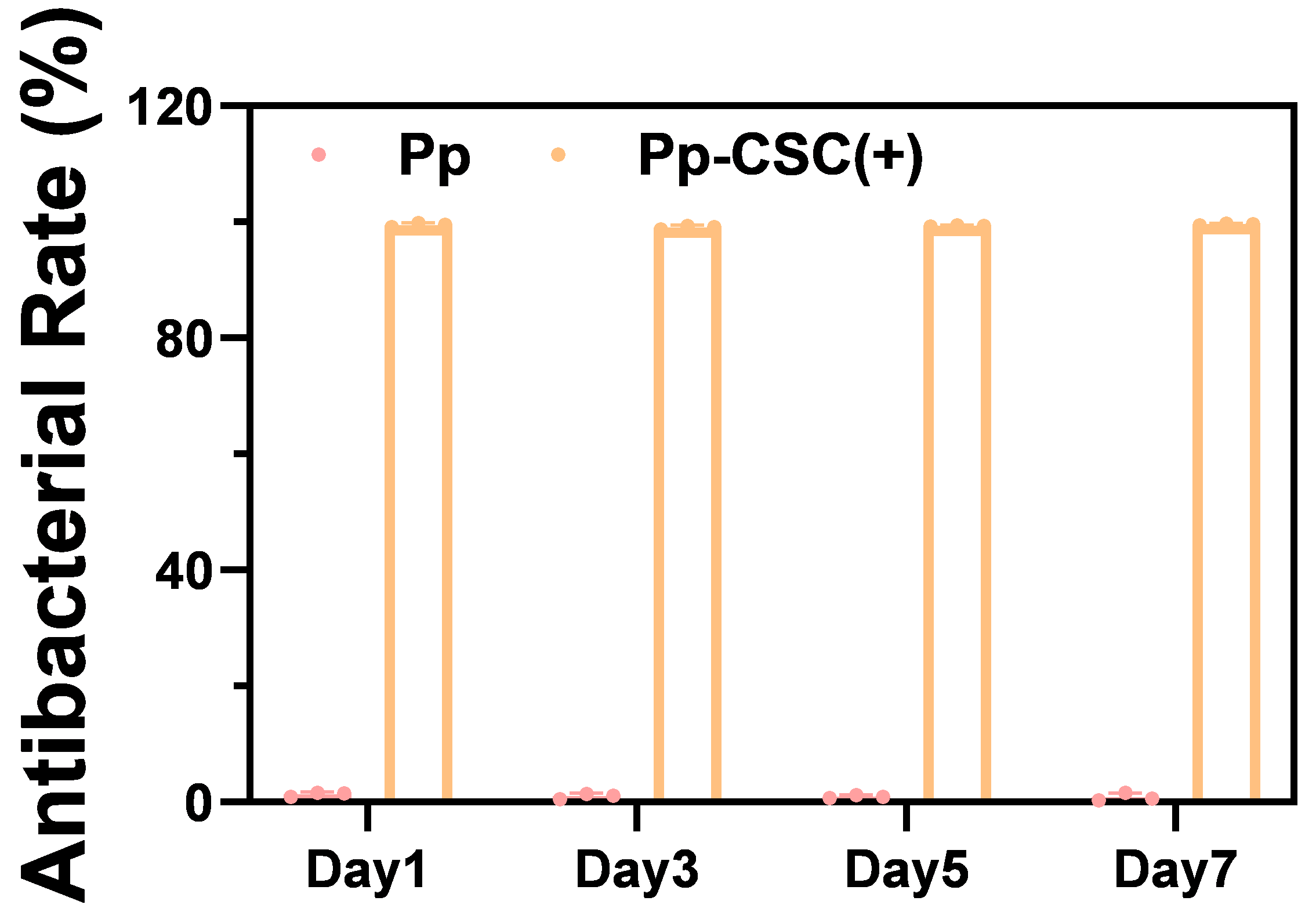


**Figure S9.** The antibacterial stability of the scaffold.
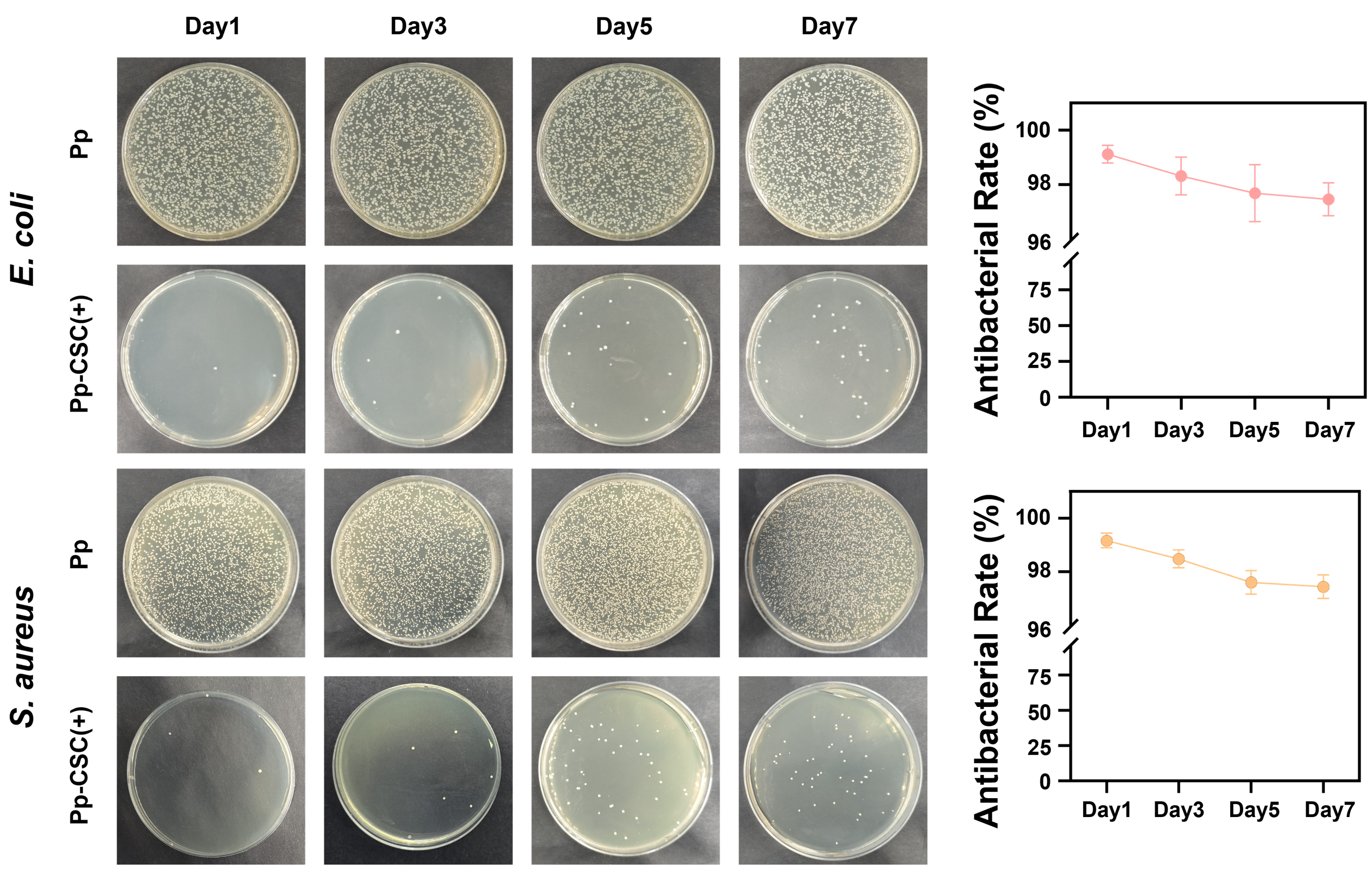


**Figure S10.** Antibacterial durability assessment in simulated body fluid (SBF). Typical images of *S. aureus* and *E. coli* colonies and quantitative measurements of sustained and renewable antibacterial rates.


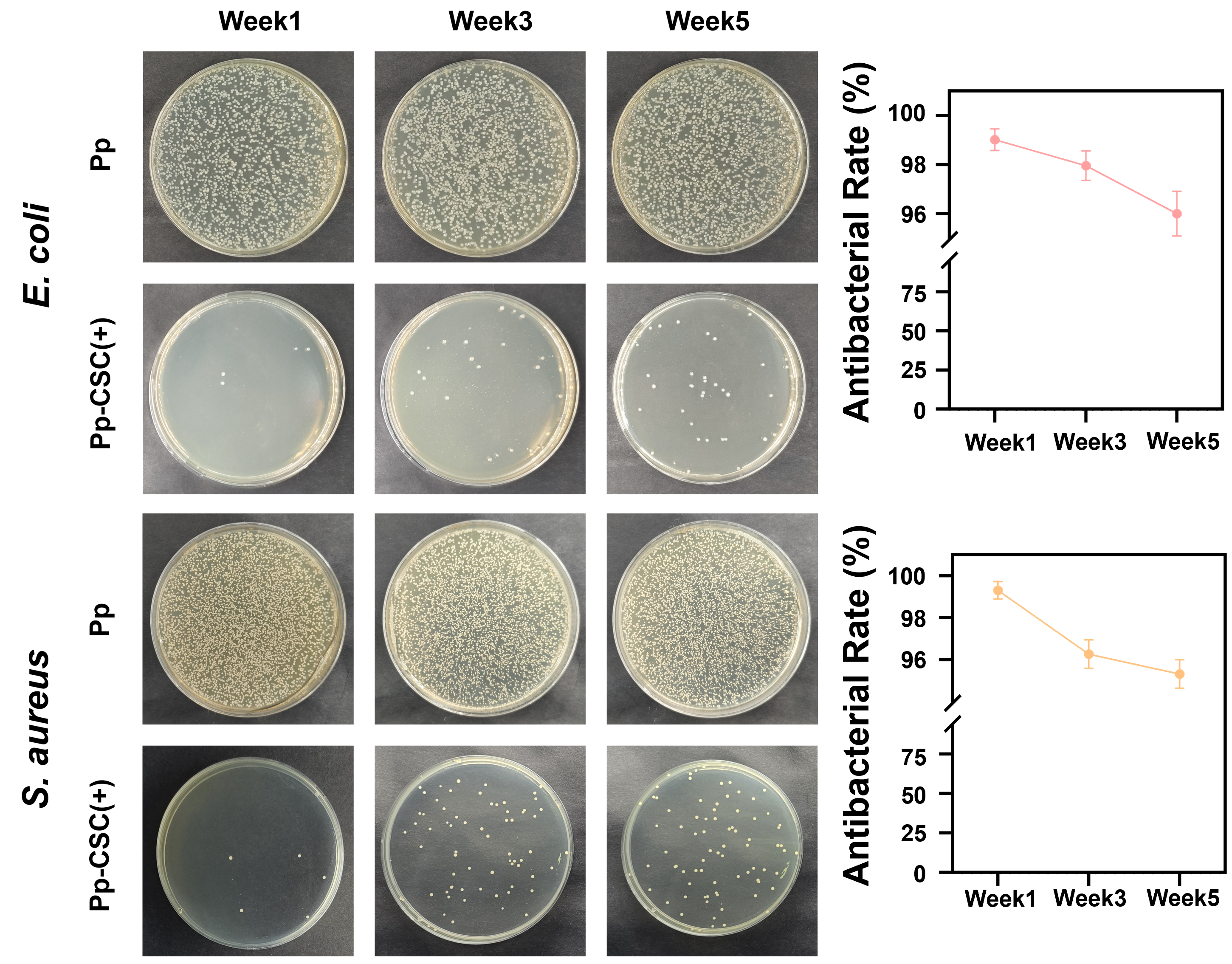


**Figure S11.** Antibacterial durability assessment under ambient conditions without treatment. Typical images of *S. aureus* and *E. coli* colonies and quantitative measurements of sustained and renewable antibacterial rates.


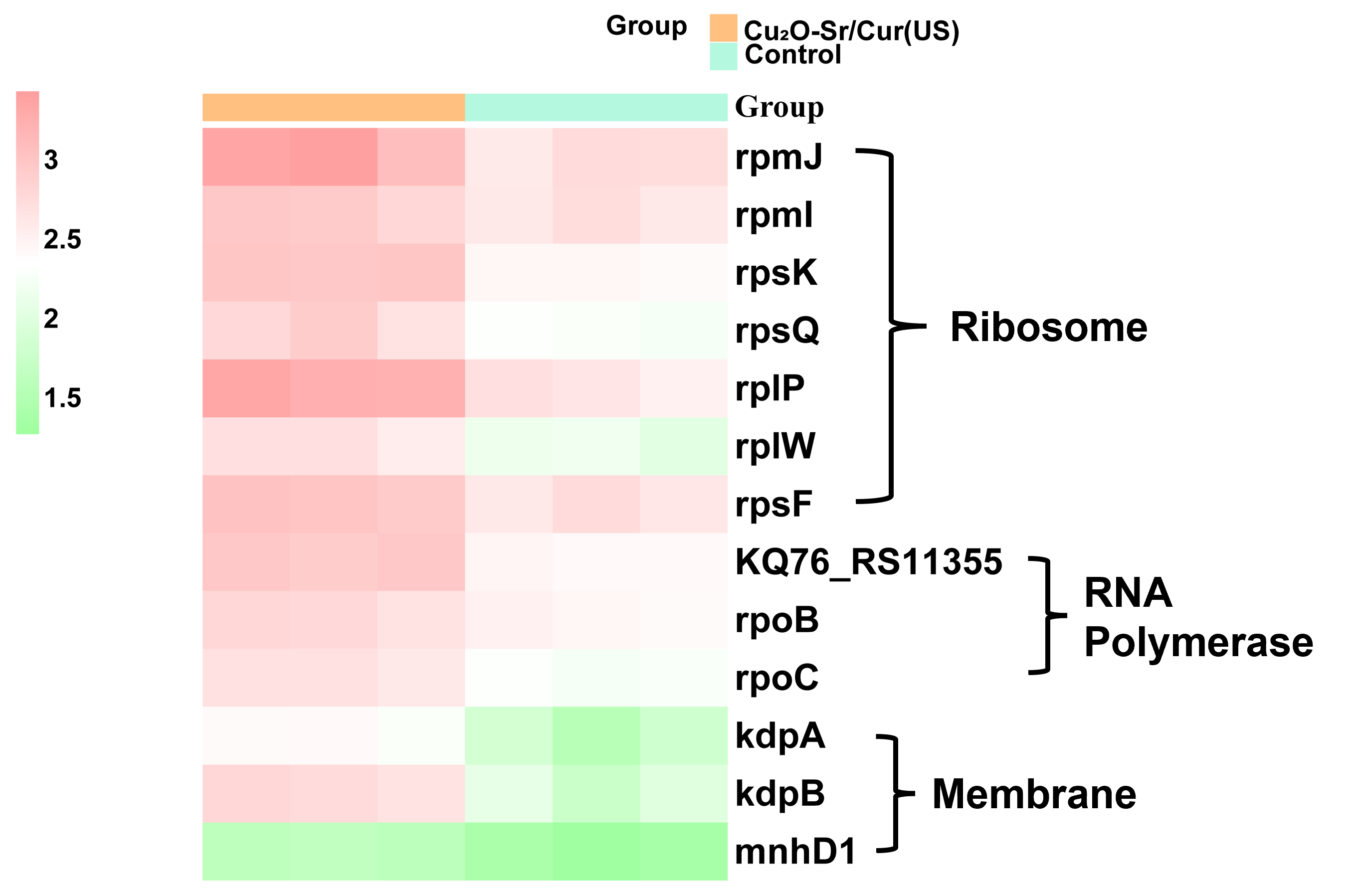


**Figure S12.** Heatmap analysis of differentially expressed genes involved in ribosome, RNA polymerase, membrane.


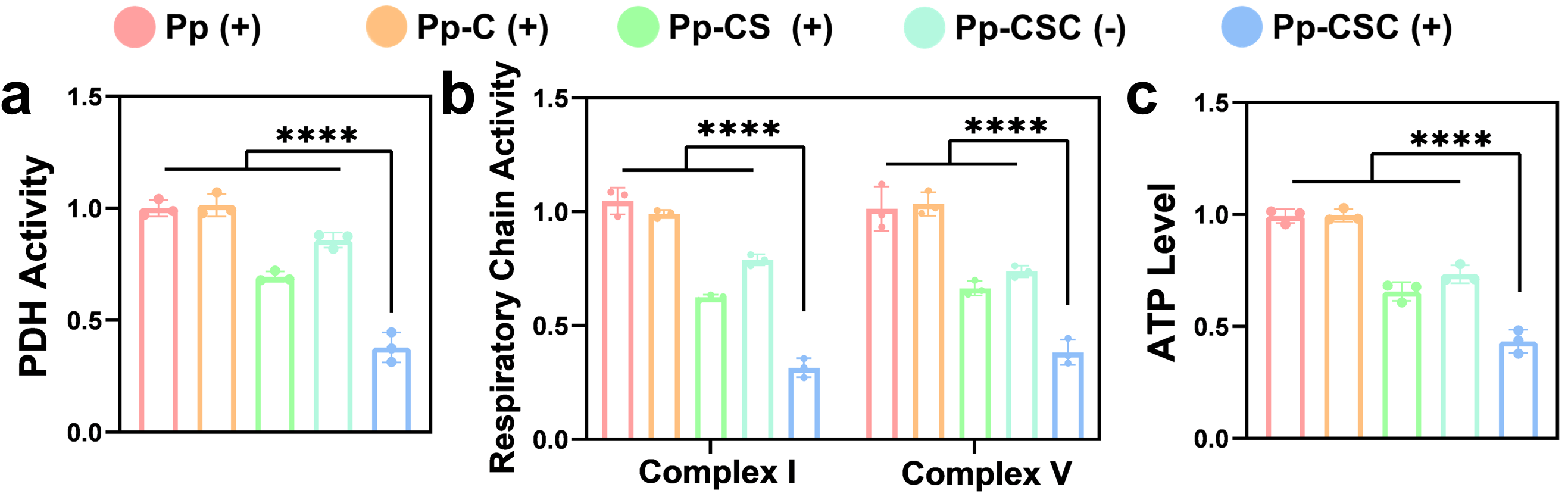


**Figure S13.** Regulation of the *S. aureus* transcriptome by different treatments. **(a)** The activity of the pyruvate dehydrogenase. **(b)** The activity of the different respiratory chain complex activity. **(c)** The production levels of ATP. The significance of biologically independent samples (n=3) was calculated by ANOVA followed by Tukey’s multiple comparisons. Data were presented as means ± SDs. Significant differences between groups were indicated as *^*^p* < 0.05, *^**^p* < 0.01, *^***^p* < 0.001, *^****^p* < 0.0001, and ns: not significance.


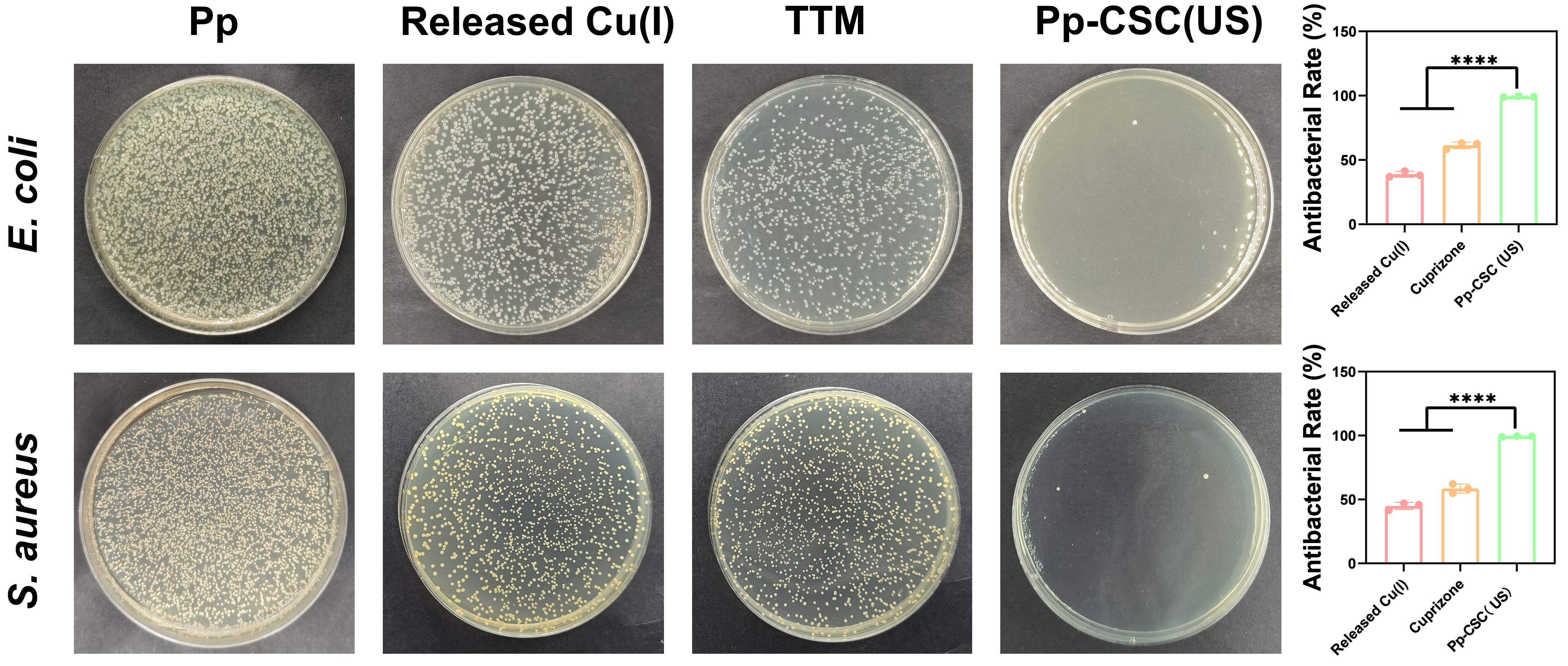


**Figure S14.** Typical images of *S. aureus* and *E. coli* colonies treated by various groups. The significance of biologically independent samples (n=3) was calculated by ANOVA followed by Tukey’s multiple comparisons. Data were presented as means ± SDs. Significant differences between groups were indicated as *^*^p* < 0.05, *^**^p* < 0.01, *^***^p* < 0.001, *^****^p* < 0.0001, and ns: not significance.

**
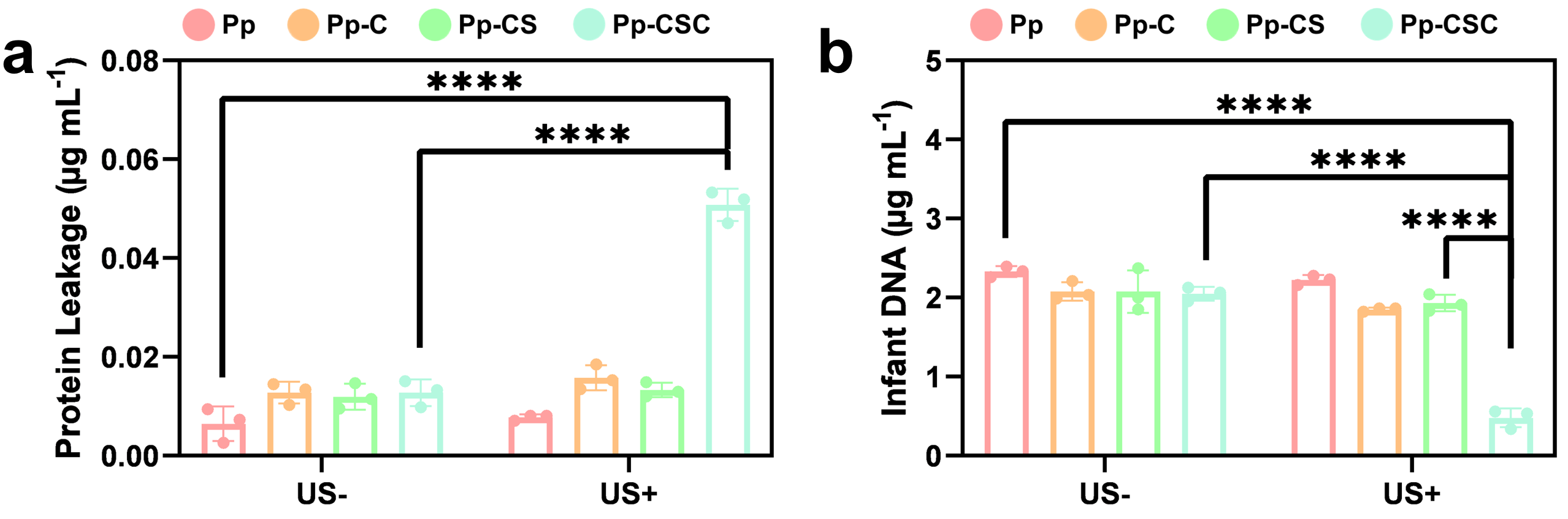
**

**Figure S15.** (**a, b**) Quantitative analysis of protein leakage(**a**) and the intact bacterial DNA (**b**) of *S. aurues* on different scaffolds after different treatments. The significance of biologically independent samples (n=3) was calculated by ANOVA followed by Tukey’s multiple comparisons. Data were presented as means ± SDs. Significant differences between groups were indicated as *^*^p* < 0.05, *^**^p* < 0.01, *^***^p* < 0.001, *^****^p* < 0.0001, and ns: not significance.


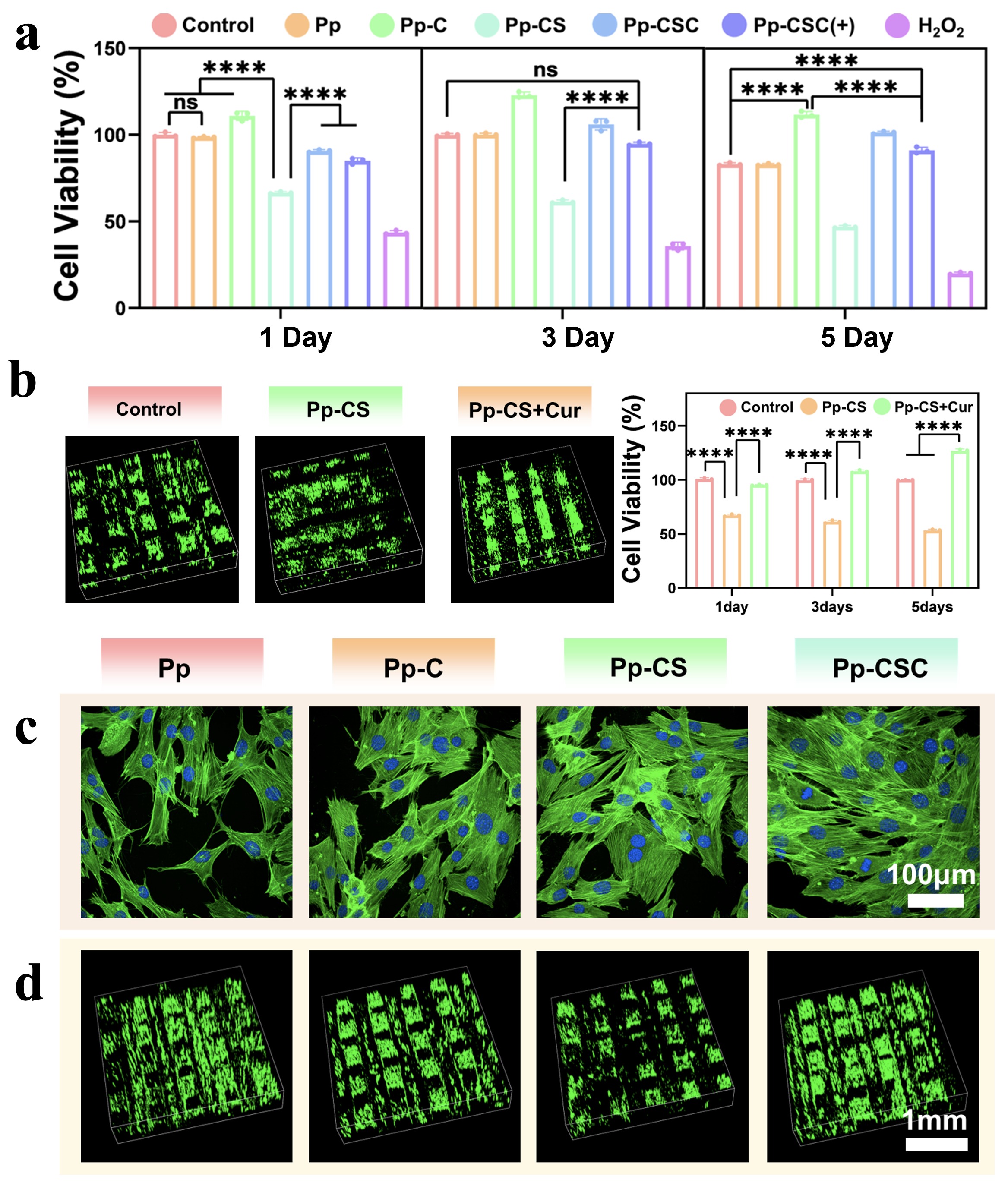


**Figure S16.** Cell viability. **(a)** MC3T3-E1 cell proliferation measured by CCK-8. **(b)** Live/DEAD staining and CCK-8 assay of MC3T3-E1 cell. **(c)** DAPI-FTIC fluorescent labeling of MC3T3 cells. **(d)** LIVE/DEAD sustainability images of MC3T3 cells. The significance of biologically independent samples (n=3) was calculated by ANOVA followed by Tukey’s multiple comparisons. Data were presented as means ± SDs. Significant differences between groups were indicated as *^*^p* < 0.05, *^**^p* < 0.01, *^***^p* < 0.001, *^****^p* < 0.0001, and ns: not significance.


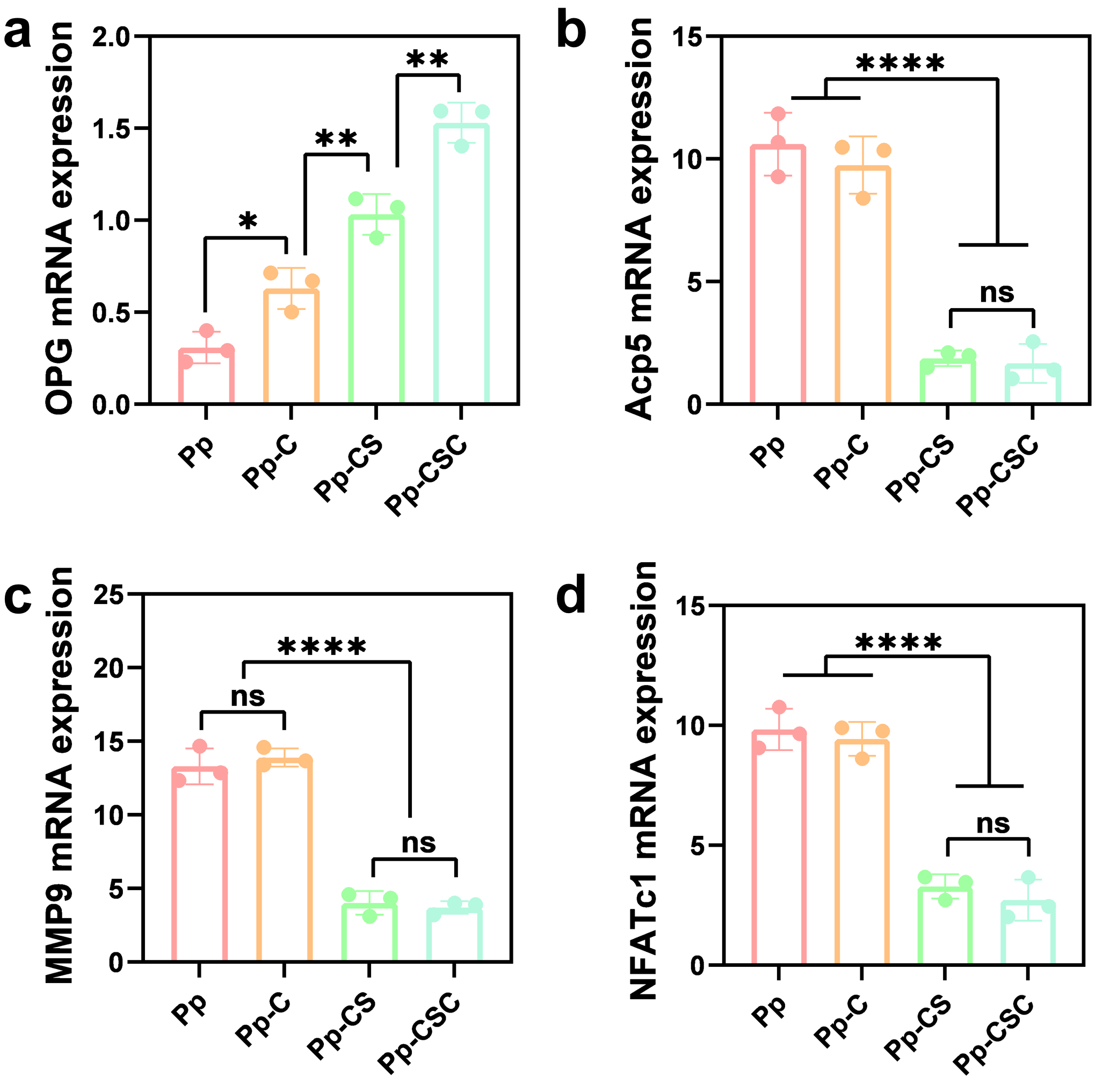


**Figure S17.** The relative mRNA expression levels of specific genes and transcription factors for osteoclast differentiation: **(a)** OPG, **(b)** Acp5, **(c)** MMP9, **(d)** NFATc1. The significance of biologically independent samples (n=3) was calculated by ANOVA followed by Tukey’s multiple comparisons. Data were presented as means ± SDs. Significant differences between groups were indicated as *^*^p* < 0.05, *^**^p* < 0.01, *^***^p* < 0.001, *^****^p* < 0.0001, and ns: not significance.


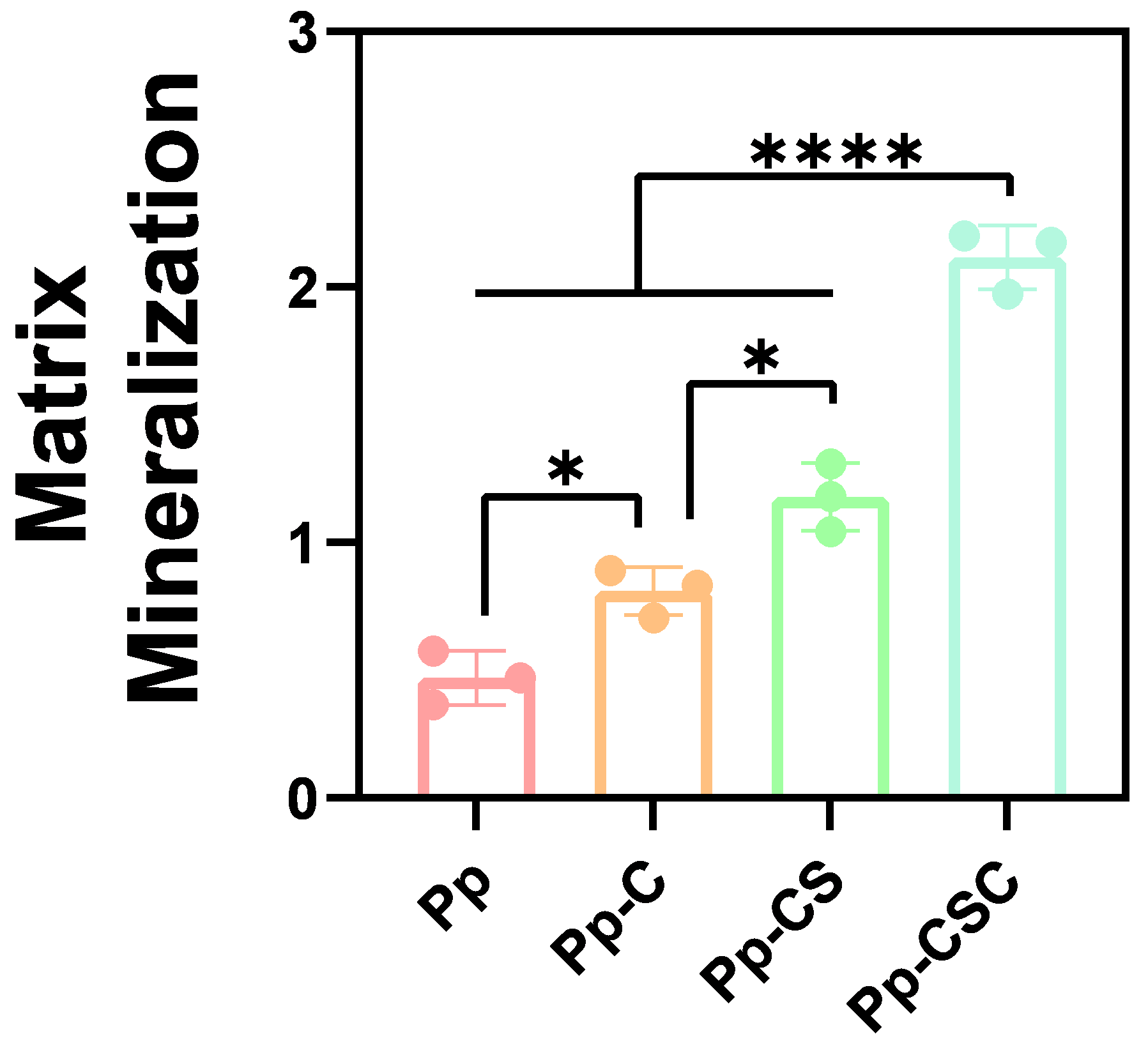


**Figure S18.** Quantiﬁcation of ARS staining after 21 days. The significance of biologically independent samples (n=3) was calculated by ANOVA followed by Tukey’s multiple comparisons. Data were presented as means ± SDs. Significant differences between groups were indicated as *^*^p* < 0.05, *^**^p* < 0.01, *^***^p* < 0.001, *^****^p* < 0.0001, and ns: not significance.


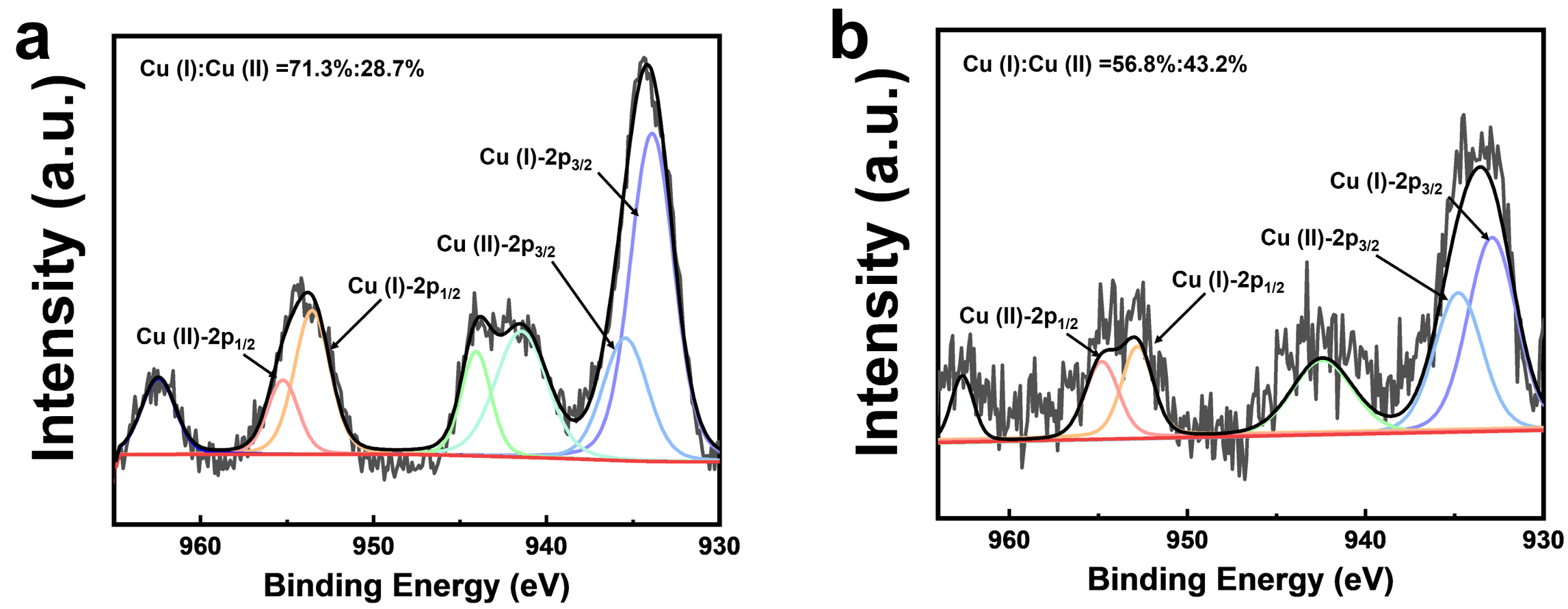


**Figure S19.** The ratio of Cu (I) to Cu (II). **(a)** The ratio of Cu (I) to Cu (II) in Cu_2_O-Sr/Cur, Cu (I): Cu (II)= 71.3%: 28.7%. **(b)** The ratio of Cu (I) to Cu (II) in Cu_2_O-Sr/Cur after reaction with 0.2mM H_2_O_2_, Cu (I): Cu (II)= 56.8%: 43.2%.


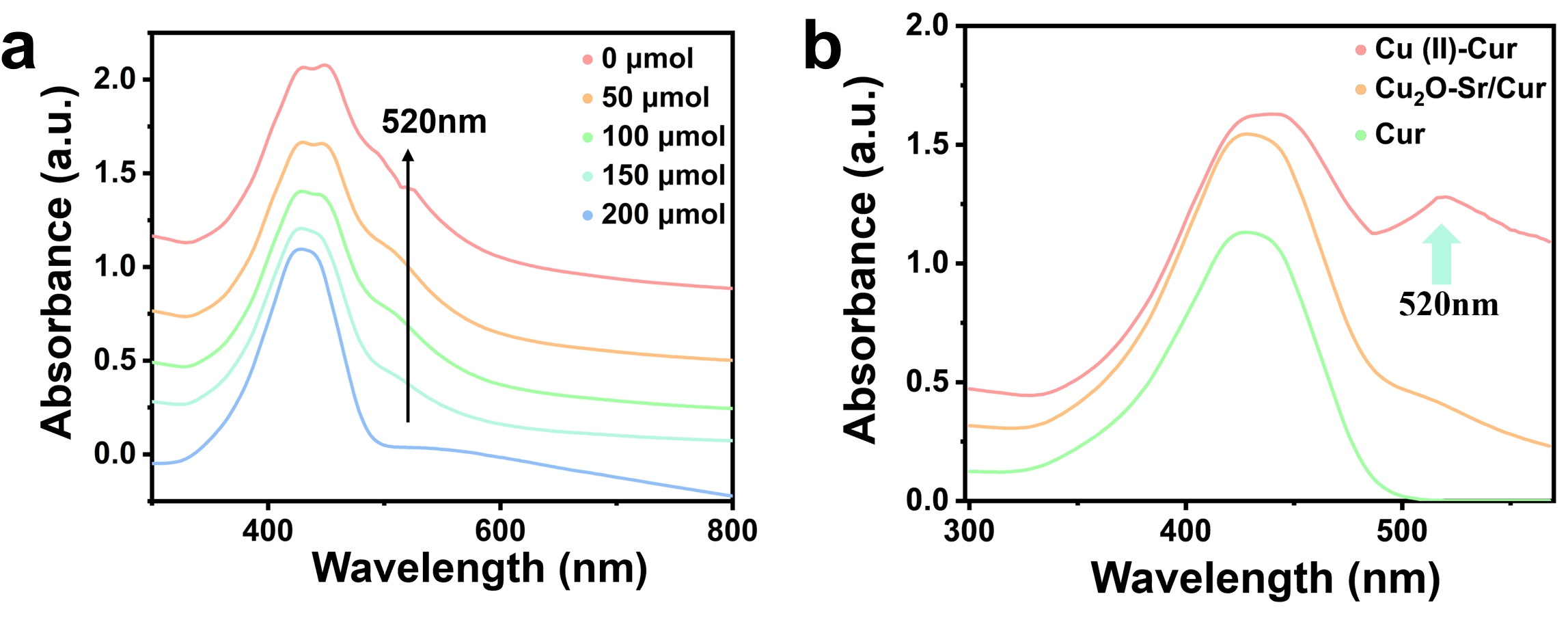


**Figure S20.** Formation of Cu (II)-Cur. **(a)** UV-Vis absorption spectrum of Cu_2_O-Sr/Cur and Cu_2_O-Sr/Cur reacting with different concentrations of hydrogen peroxide. **(b)** UV-Vis absorption spectra of Cur, Cu_2_O-Sr/Cur, and Cu (II)-Cur.


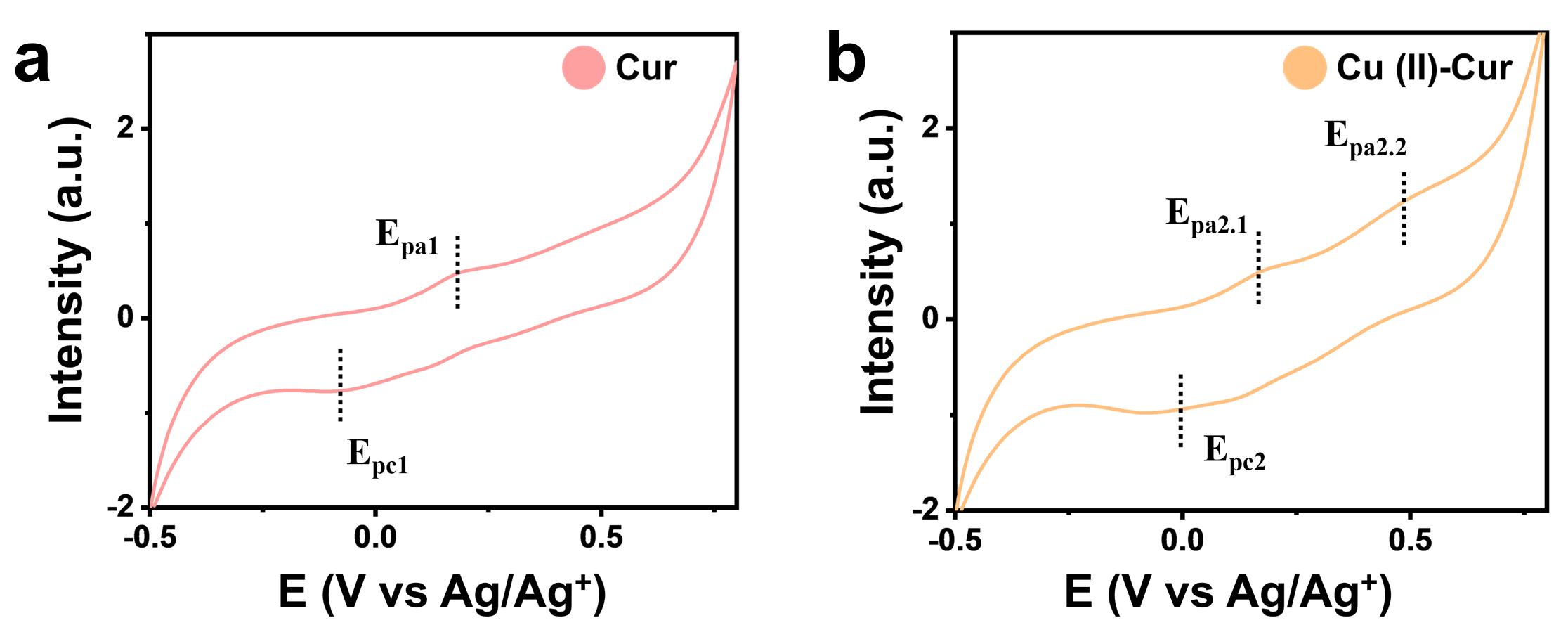


**Figure S21.** CV curves evaluating the electrochemical behaviors of Cur **(a)** and Cu (II)-Cur **(b)** in an acidic PBS solution (pH = 6.5).


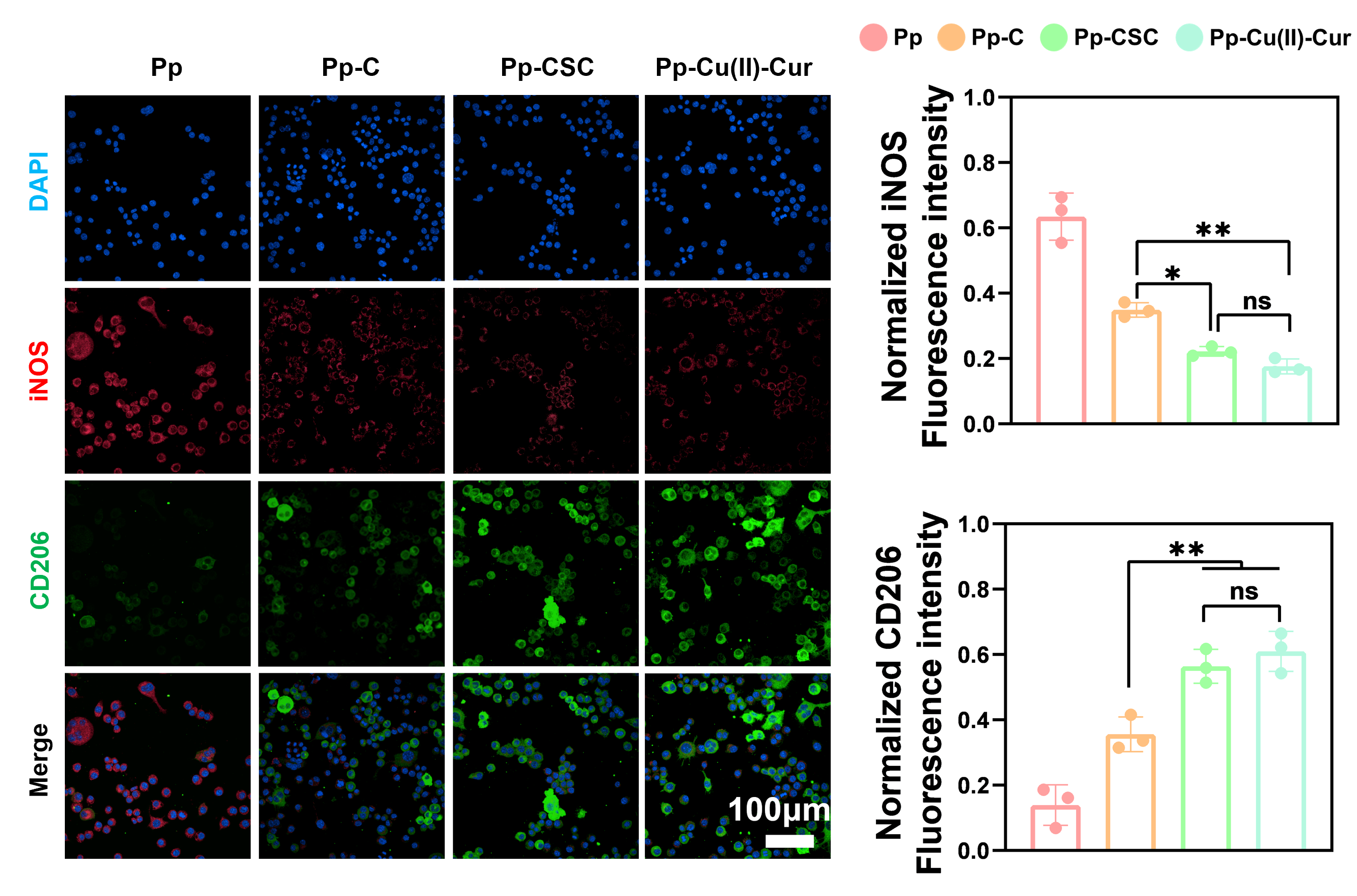


**Figure S22.** Cellular anti-inflammatory effects. The significance of biologically independent samples (n=3) was calculated by ANOVA followed by Tukey’s multiple comparisons. Data were presented as means ± SDs. Significant differences between groups were indicated as *^*^p* < 0.05, *^**^p* < 0.01, *^***^p* < 0.001, *^****^p* < 0.0001, and ns: not significance.


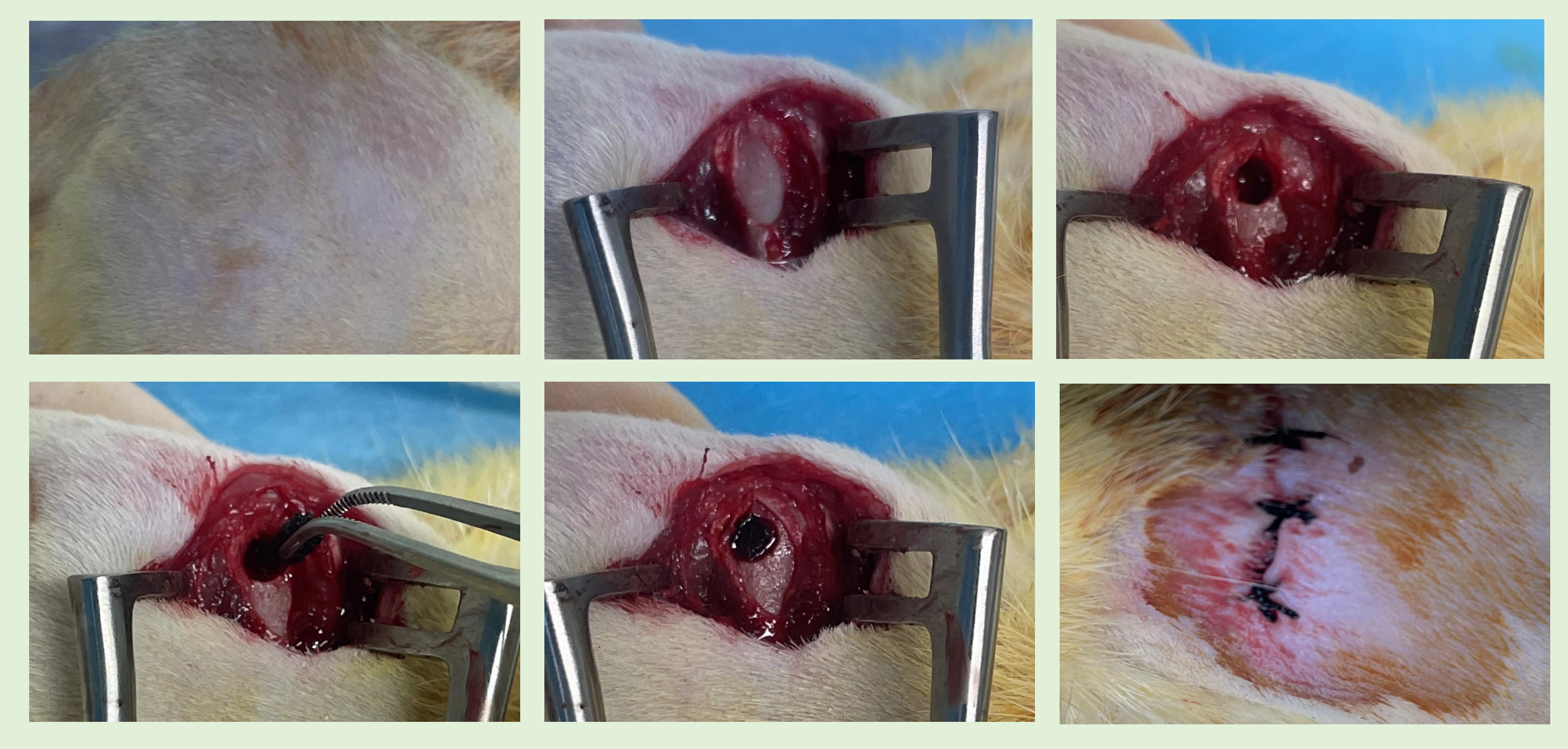


**Figure S23.** Construction of animal models.


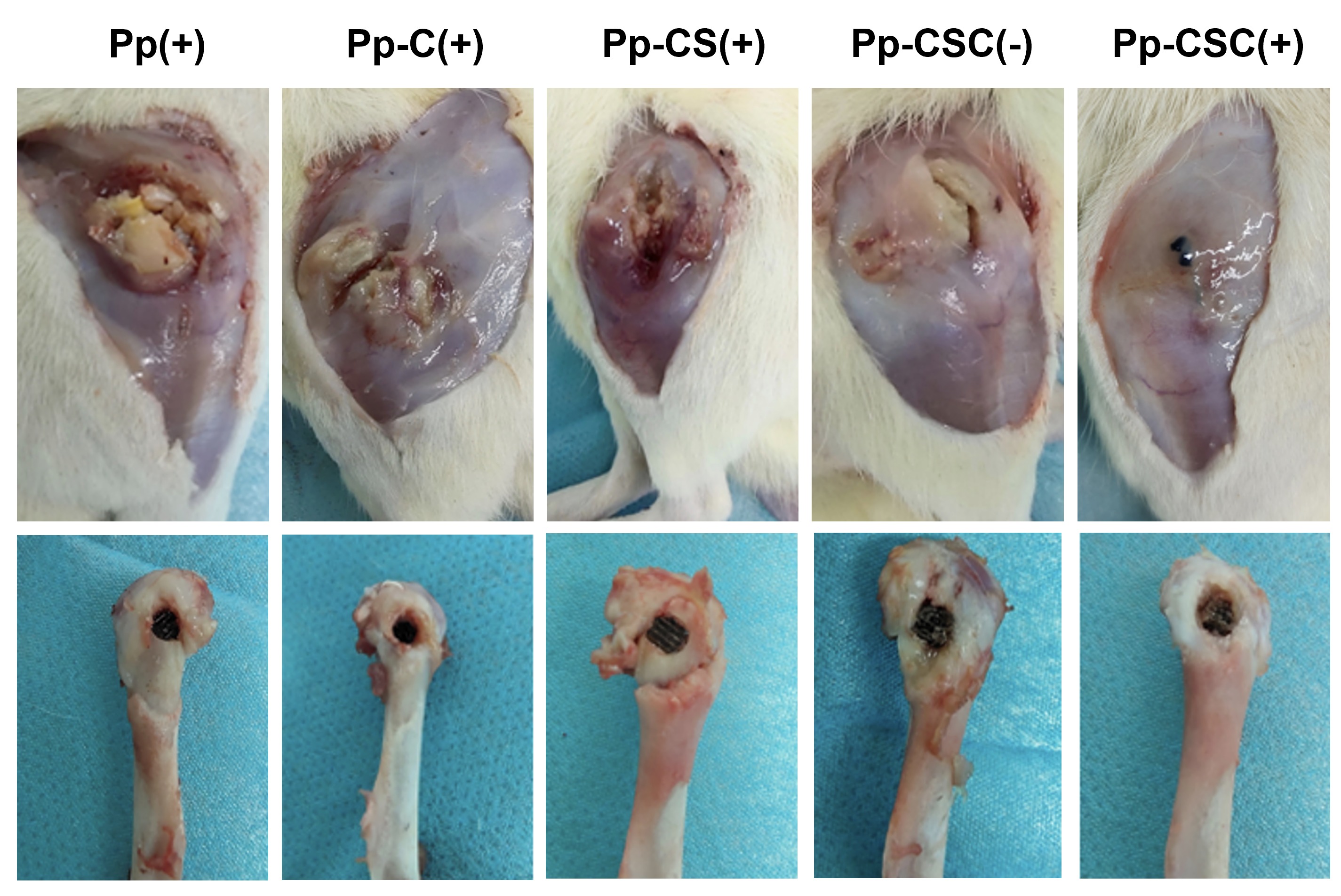


**Figure S24.** Appearance of the femoral condyle in rats after 7 days.


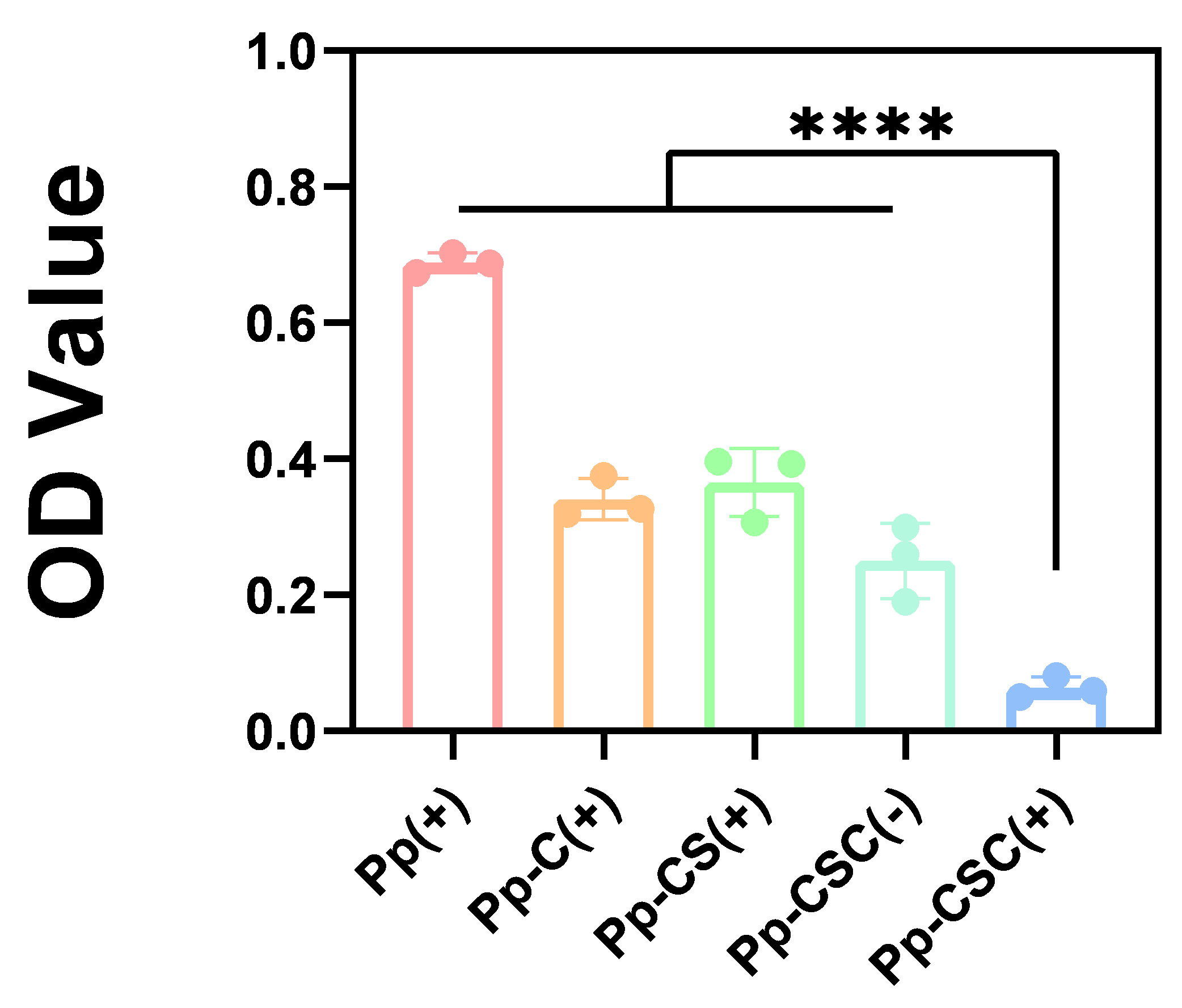


**Figure S25.** OD_600_ value of Bacterial liquid medium soaked in scaffolds each treatment in vivo. The significance of biologically independent samples (n=3) was calculated by ANOVA followed by Tukey’s multiple comparisons. Data were presented as means ± SDs. Significant differences between groups were indicated as *^*^p* < 0.05, *^**^p* < 0.01, *^***^p* < 0.001, *^****^p* < 0.0001, and ns: not significance.


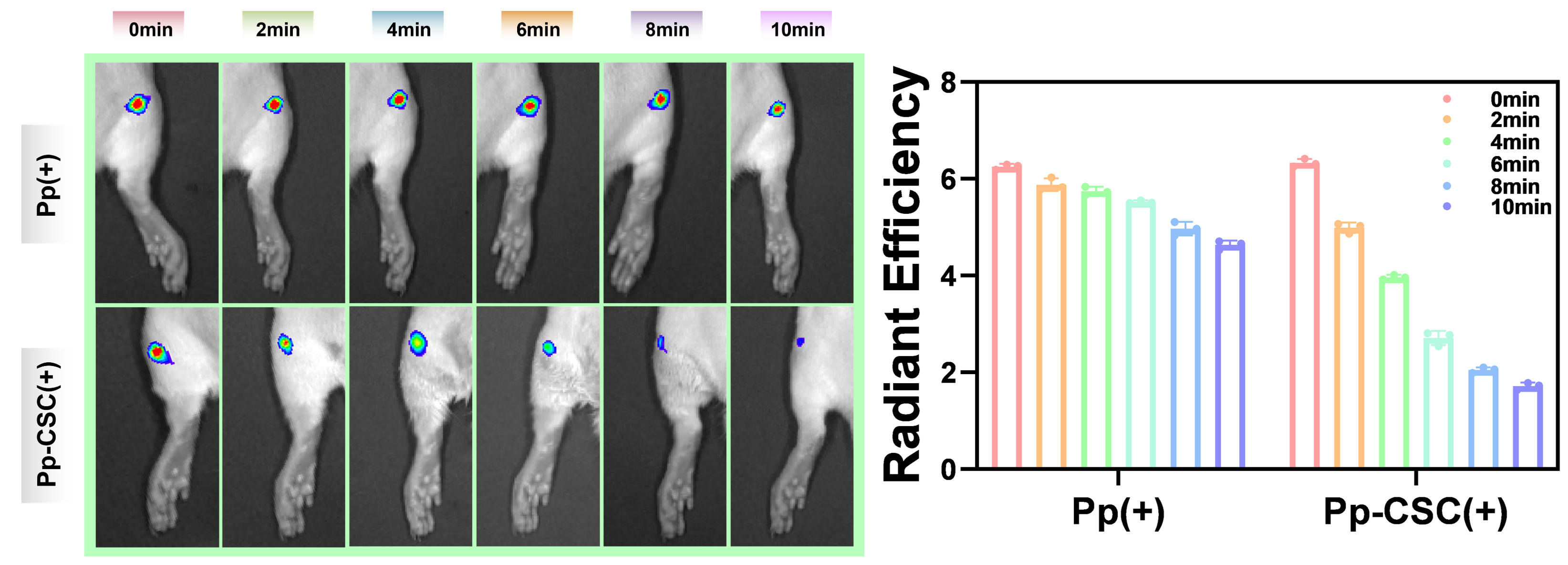


**Figure S26.** Optical imaging of mice in vivo for real-time detection of effects of different treatments on DiR iodide-labeled *S. aureus.*

**
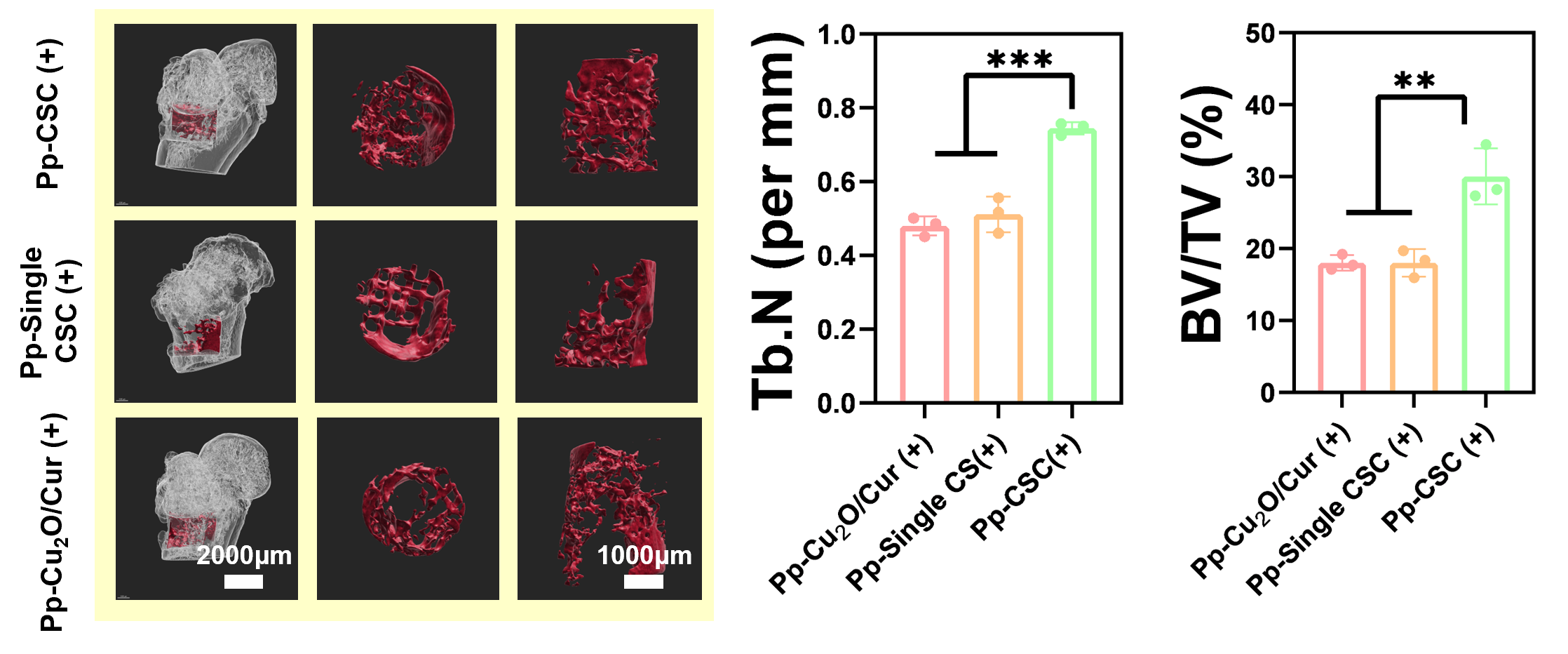
**

**Figure S27.** 3D reconstruction images of Micro-CT by IMARIS regarding new bone formation at week 4 and quantiﬁcation of BV/TV, Tb.N. The significance of biologically independent samples (n=3) was calculated by ANOVA followed by Tukey’s multiple comparisons. Data were presented as means ± SDs. Significant differences between groups were indicated as *^*^p* < 0.05, *^**^p* < 0.01, *^***^p* < 0.001, *^****^p* < 0.0001, and ns: not significance.


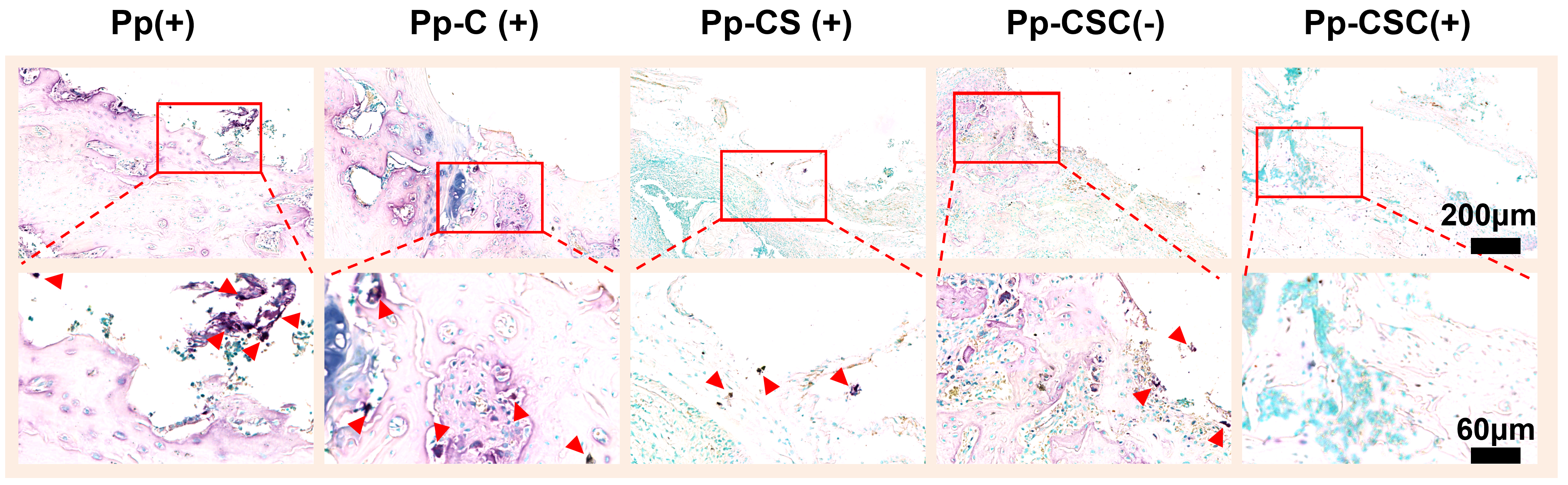


**Figure S28.** Immunohistochemistry staining of TRAP proteins surrounding the implants at week 8.


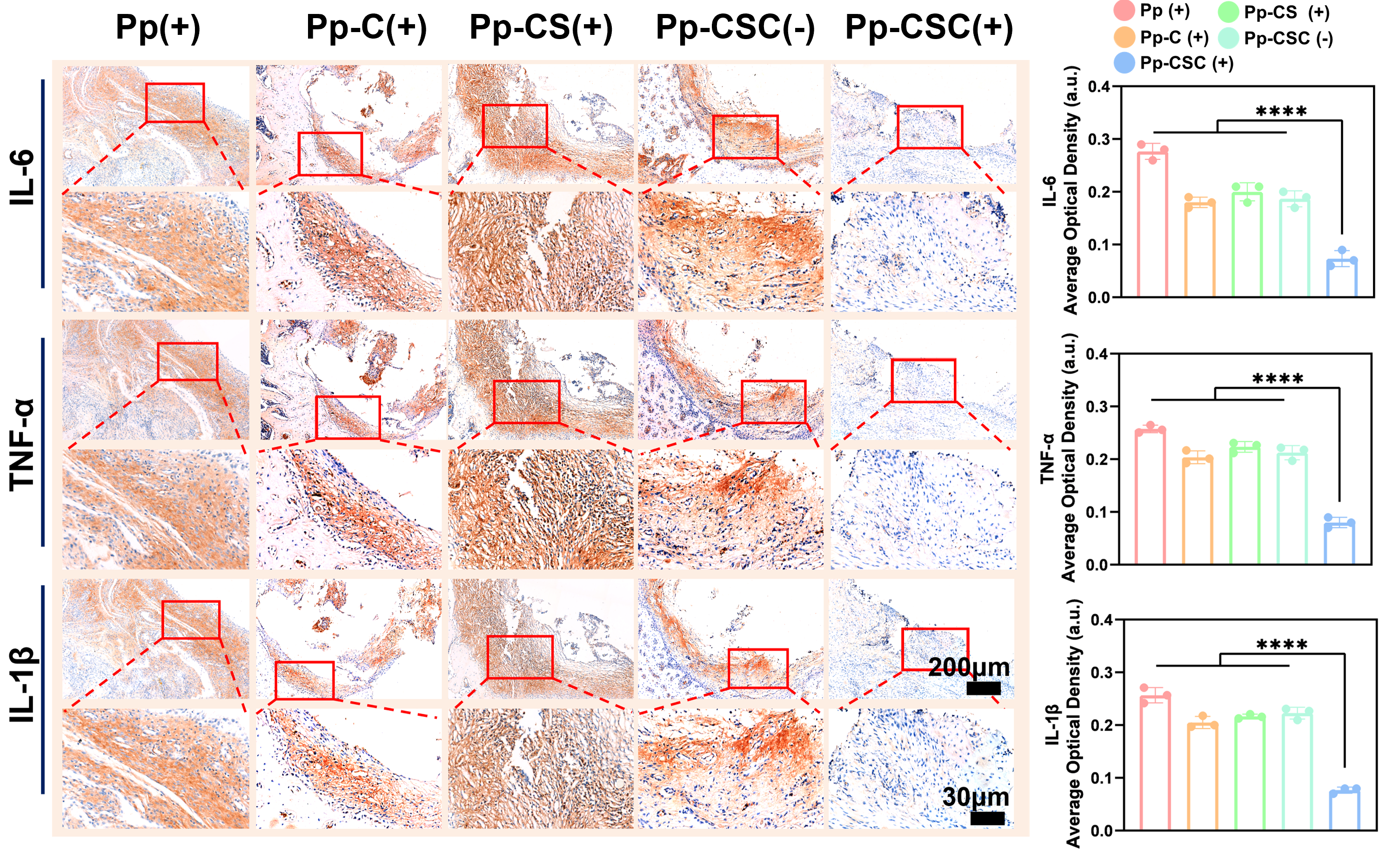


**Figure S29.** Immunohistochemistry staining of TNF-α, IL-6 and IL-1β proteins surrounding the implants at week 8. The significance of biologically independent samples (n=3) was calculated by ANOVA followed by Tukey’s multiple comparisons. Data were presented as means ± SDs. Significant differences between groups were indicated as *^*^p* < 0.05, *^**^p* < 0.01, *^***^p* < 0.001, *^****^p* < 0.0001, and ns: not significance.


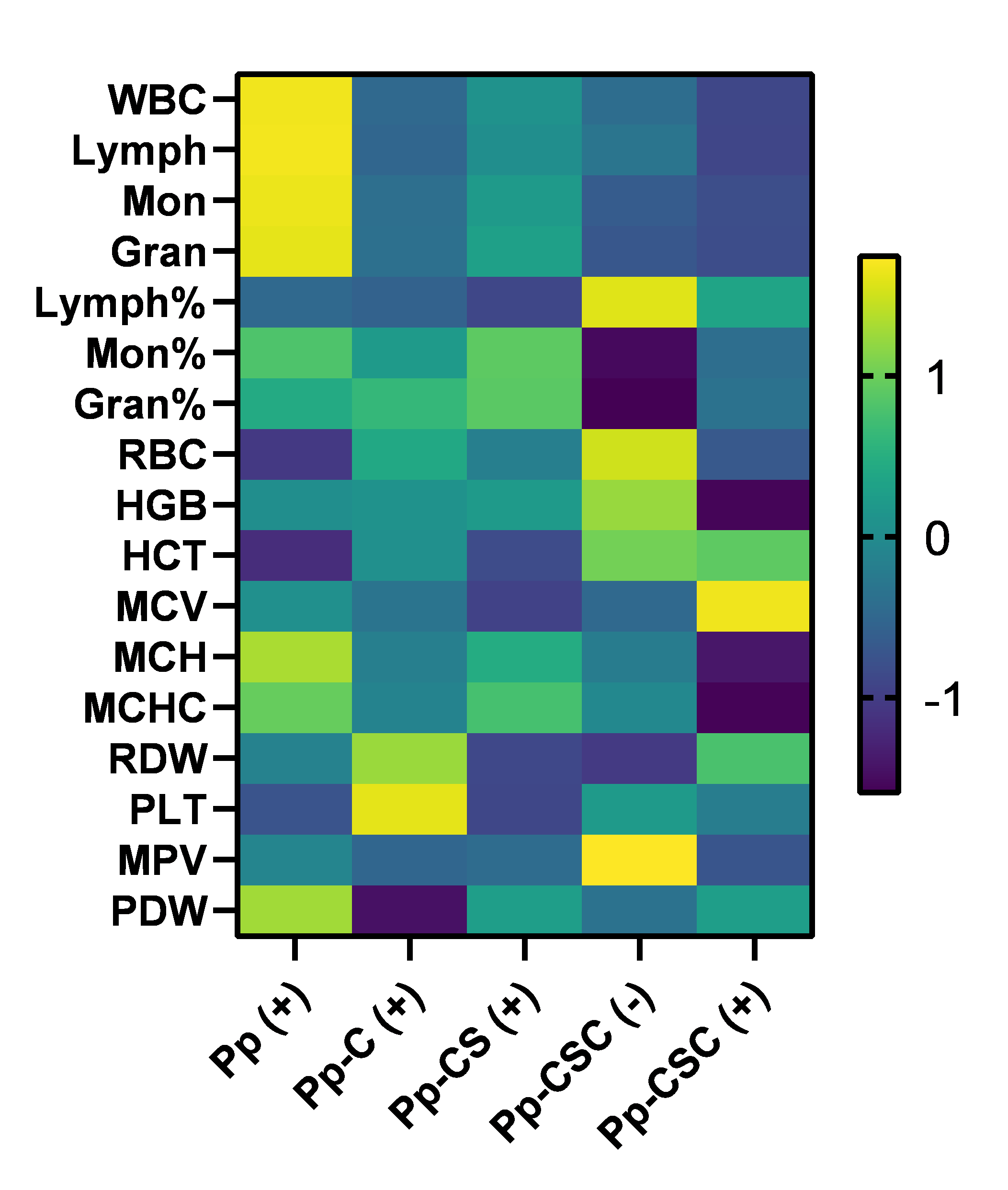


**Figure S30.** The blood routine of different groups (mean, n = 3).

**
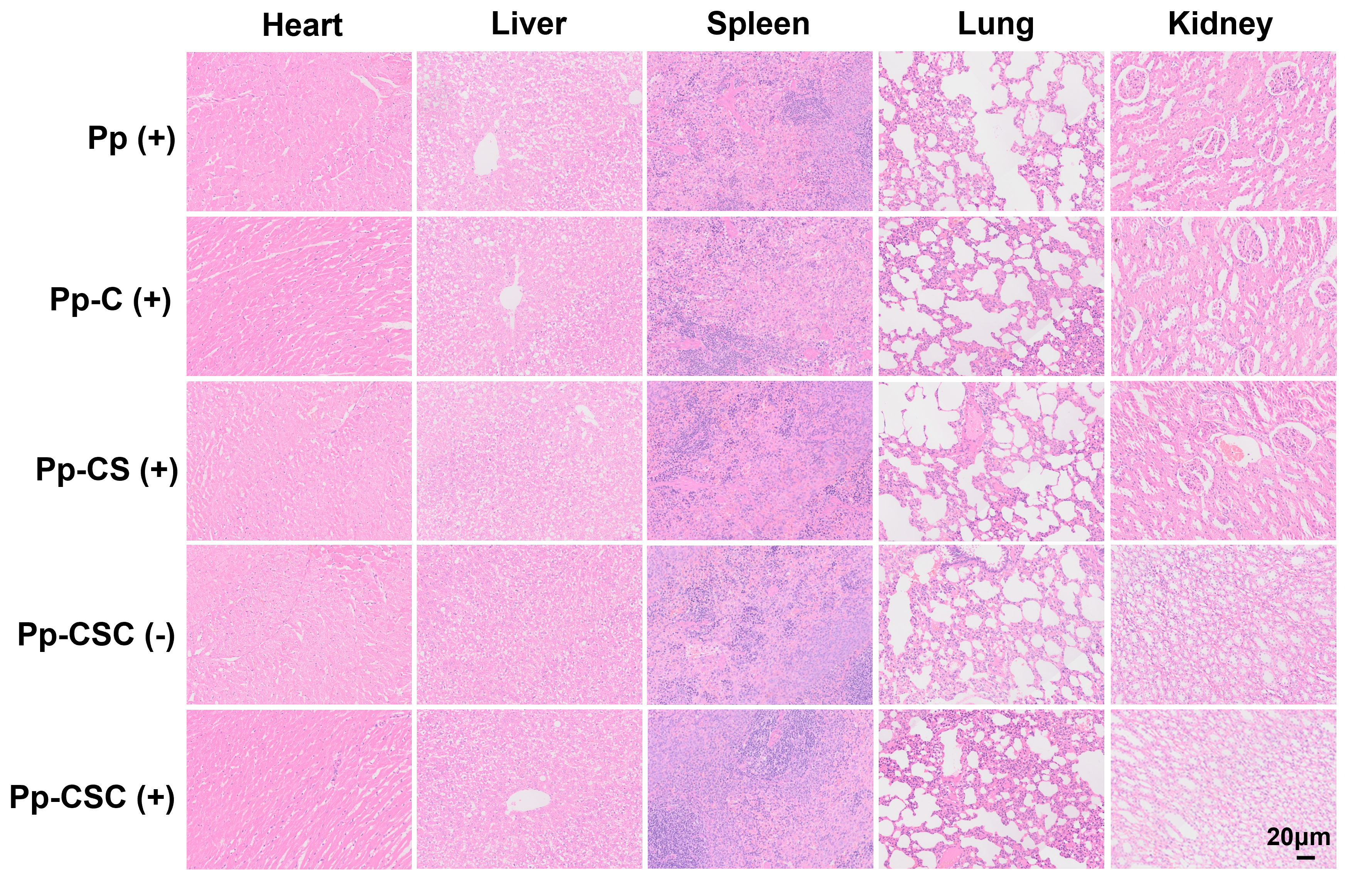
**

**Figure S31.** The H&E staining of major organs including heart, liver, spleen, lung and kidney in different groups.

**
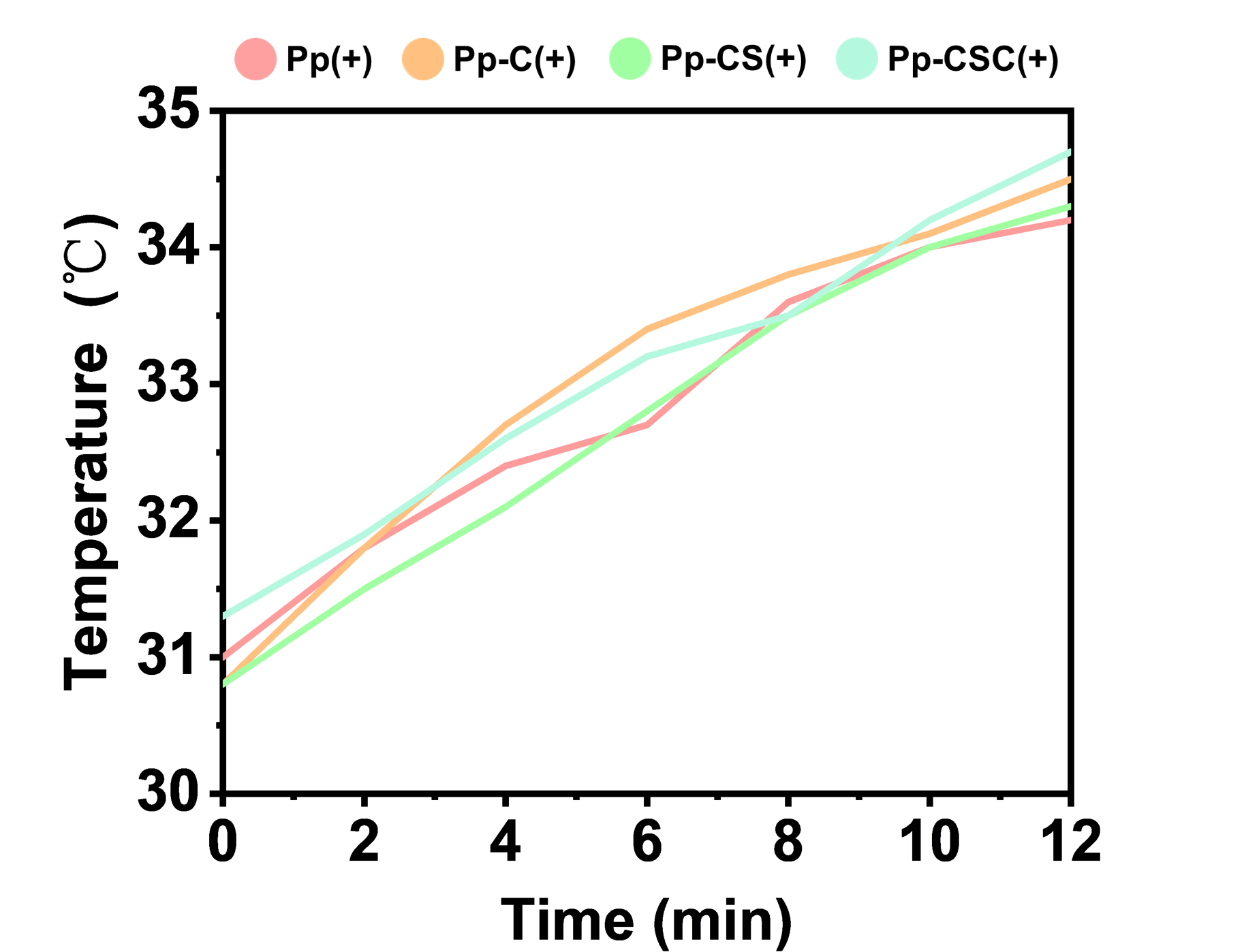
**

**Figure S32.** The temperature of different groups after ultrasound.

**2. Tables of supporting information**

| **Bond length (Å)** | | | **Bond angle (°)** | | |
| --- | --- | --- | --- | --- | --- |
| Cu•••O | Before | After | Cu•••O | Before | After |
| 1 | 1.84881 | 1.83208 | 1 | 107.70430 | 109.46680 |
| 2 | 1.84895 | 1.87979 | 2 | 109.47330 | 105.44620 |
| 3 | 1.83936 | 1.84699 | 3 | 109.46840 | 104.67310 |
| 4 | 1.83290 | 1.84571 | 4 | 108.46360 | 106.99660 |
| 5 | 1.89943 | 1.89913 |  |  |  |
| 6 | 1.84059 | 1.84664 |  |  |  |
| 7 | 1.87490 | 1.88310 |  |  |  |

**Table S1.** The changes of bond lengths and bond angles of before and after contact.

|  | **H•••O** | | **O•••Cu** | |
| --- | --- | --- | --- | --- |
| Bond length (Å) | 1 | 2 | 1 | 2 |
|  | 2.52773 | 2.65090 | 2.90433 | 2.88081 |

**Table. S2.** The information of bond lengths after contact.

| Genes |  | Primers (5'-3') |
| --- | --- | --- |
| OPG | Forward | GAGGAGTCTGGTAGTGGTTCC |
| OPG | Reverse | GGGCGTTTCGTTGAATATGCG |
| Acp5 | Forward | TTACTACCGTTTGCGCTTC |
| Acp5 | Reverse | CATTTTGGGCTGCTGACT |
| MMP-9 | Forward | CGACTTTTGTGGTCTTCCCC |
| MMP-9 | Reverse | CTTCTCTCCCATCATCTGGGC |
| NFATc1 | Forward | CCTTCAGAGAGACCTTGGC |
| NFATc1 | Reverse | CACAGGAGCTGGGGTTC |
| β-actin | Forward | TGCTGTCCCTGTATGCCTCTG |
| β-actin | Reverse | TGATGTCACGCACGATTTCC |

**Table S3.** Gene sequences of landmark genes and transcription factors for osteoclast differentiation and osteogenic differentiation.
